# Optimal Head-of-Bed Positioning Before Thrombectomy in Large Vessel Occlusion Stroke: A Randomized Clinical Trial

**DOI:** 10.1001/jamaneurol.2025.2253

**Published:** 2025-06-04

**Authors:** Anne W. Alexandrov, Anne J. Shearin, Pitchaiah Mandava, Gabriel Torrealba-Acosta, Cheran Elangovan, Balaji Krishnaiah, Katherine Nearing, Elizabeth Robinson, Cara Guthrie-Chu, Matthew Holzmann, Bryan Fill, Dharti R. Trivedi, Alicia Richardson, Sandy Middleton, Barbara B. Brewer, David S. Liebeskind, Nitin Goyal, James C. Grotta, Andrei V. Alexandrov

**Affiliations:** 1 University of Tennessee Health Science Center, Memphis; 2 Department of Neurology, Baylor College of Medicine, Houston, Texas; 3 Department of Neurology, Duke University, Durham, North Carolina; 4 Baptist Memorial Hospital, Memphis, Tennessee; 5 Medical City, Fort Worth, Texas; 6 St Francis Health System, Tulsa, Oklahoma; 7 Northwestern Medicine Central DuPage Hospital, Winfield, Illinois; 8 Doctors Medical Center of Modesto Inc, Modesto, California; 9 Penn State Health Milton S. Hershey Medical Center, Hershey, Pennsylvania; 10 School of Nursing, Midwifery, and Paramedicine, Australian Catholic University, Sydney, New South Wales, Australia; 11 Nursing Research Institute, St Vincent’s Health Network Sydney and St Vincent’s Hospital, Melbourne, Victoria, Australia; 12 College of Nursing, University of Arizona, Tucson; 13 Department of Neurology, University of California, Los Angeles; 14 Semmes-Murphy Neurosurgery, Memphis, Tennessee; 15 Memorial Hermann Medical Center, Houston, Texas; 16 Department of Neurology, University of Arizona College of Medicine-Phoenix and Banner University Medical Center, Phoenix

## Abstract

**Importance:**

Small studies show that 0° head positioning of patients with large vessel occlusion (LVO) stroke improves penumbral blood flow and clinical stability. Understanding whether 0° head position maintains clinical stability would allow for optimal patient positioning before thrombectomy.

**Objective:**

To determine superiority of 0° over 30° head positioning at maintaining clinical stability in patients with LVO before thrombectomy.

**Design, Setting, and Participants:**

This was a prospective randomized clinical trial with blinding to study enrollment/end points conducted from May 2018 to November 2023. There were 3 planned interim analyses, and the study was conducted at certified thrombectomy hospitals in the US. Included in this analysis were consecutive consenting individuals with computed tomography (CT) angiography–positive anterior or posterior LVO who were candidates for thrombectomy (baseline mRS 0-1) and had viable penumbra (CT perfusion or Alberta Stroke Program Early Computed Tomography Score ≥6) within 24 hours of stroke onset. Enrollment of systemic thrombolysis more than 15 minutes from consent was discouraged to prevent confounding of head position effects; in addition, patients with disabilities who lacked a legal representative could not participate due to lack of consent.

**Interventions:**

Randomization to 0° or 30° head positioning with monitoring every 10 minutes using the National Institutes of Health Stroke Scale (NIHSS) until movement to a catheterization table.

**Main Outcome and Measures:**

The primary outcome was worsening of 2 or more NIHSS points before thrombectomy. Safety outcomes included severe neurologic deterioration (worsening ≥4 NIHSS points) before thrombectomy, hospital-acquired pneumonia (HAP) during hospitalization, and all-cause death within 3 months.

**Results:**

Planned enrollment included 182 patients. Before data and safety monitoring board study closure, a total of 92 patients (mean [SD] age, 66.6 [14.4] years; 48 male [52.2%]) were randomized: 45 patients to the group with 0° head positioning and 47 patients to the group with 30° head positioning. Patient characteristics were similar between groups; however, patients with head position at 30° experienced worsening on the NIHSS of 2 points or more, whereas patients with head position at 0° showed score stability (hazard ratio [HR], 34.40; 95% CI, 4.65-254.37; *P* < .001). One patient with 0° head positioning and 20 patients with 30° head positioning experienced worsening on the NIHSS of 4 points or more during positioning (HR, 23.57; 95% CI, 3.16-175.99; *P* = .002). No patients developed HAP; all-cause death occurred in 2 patients (4.4%) in the 0° group, compared with 10 patients (21.7%; *P* = .03) in the 30° group.

**Conclusions and Relevance:**

Results suggest that 0° head positioning for patients with acute LVO was a protective maneuver to maintain clinical stability in the prethrombectomy phase while awaiting definitive treatment.

**Trial Registration:**

ClinicalTrials.gov Identifier: NCT03728738

## Key Points

**Question:**

Is use of a 0° head position superior to a 30° head position at maintaining clinical stability in individuals with large vessel occlusion (LVO) stroke who are candidates for thrombectomy and are awaiting definitive thrombectomy treatment?

**Findings:**

In this randomized clinical trial including 92 patients, use of a 0° head position was safe and prevented clinical worsening, whereas a 30° head position resulted in a 34.4-fold increased hazard of clinical worsening before thrombectomy treatment.

**Meaning:**

Results suggest that patients awaiting thrombectomy treatment for LVO stroke should be positioned with the head at 0° to ensure clinical stability and prevent worsening.

## Teaser

This randomized clinical trial investigates if use of a 0° head position is superior to a 30° head position for maintaining clinical stability in individuals with large vessel occlusion stroke who are candidates for thrombectomy and are awaiting definitive thrombectomy treatment.

## Introduction

Whether 0° or 30° head position is important to maintain clinical stability in patients with large vessel occlusion (LVO) before thrombectomy has never, to our knowledge, been explored outside small observational studies^1,2,3,4,5,6,7,8,9^ in the prethrombectomy era, suggesting benefit. Toole^10^ identified clinical change with head positioning in 1968, noting that some patients with stroke deteriorated markedly when the head was elevated; these findings were again identified in 1976 by Caplan and Sergay,^11^ who coined the term *positional cerebral ischemia*. During the NINDS rt-PA Stroke Study enrollment, investigators were perplexed by the opposite effect, with neurological improvement after 0° positioning for computed tomography (CT), leading to deferment of deficit scoring until after CT completion (J.C. Grotta, MD, verbal communication, December 21, 2023).

Our transcranial Doppler (TCD) pilot work showed increased LVO residual blood flow^3,4^ with neurological improvement when heads were lowered from 30°to 0°.^4^ Similar findings have been documented by others^5,6,7^ as well as clinical worsening with LVO head elevation.^8^ However, the Head Positioning in Stroke Trial showed that head position alone should not be considered a treatment for stroke,^12,13^ and given thrombectomy’s effect size,^14,15,16,17,18,19,20,21^ attempts to amplify 3-month outcomes with head positioning would be challenging, especially in patients arriving directly to thrombectomy hospitals.

Delays to thrombectomy treatment can be substantial.^22,23^ In patients transferred between facilities, door-in/door-out times average over 2 hours nationally,^22^ and treatment delays worsen thrombectomy outcomes.^24,25^ Given our pilot findings, we sought to determine whether 0° head positioning of patients with LVO who were candidates for thrombectomy was associated with greater clinical stability/improvement compared with 30°, making it an important protective maneuver before thrombectomy.

## Methods

### Trial Design and Oversight

The ZODIAC trial was a National Institutes of Health–funded, multisite, prospective, outcome-blinded randomized clinical trial examining clinical stability in patients with LVO who were randomized to either a 0° or 30° head position before planned thrombectomy. The protocol was approved by participating site institutional review boards and affiliated statistical, fidelity review, and neuroimaging core laboratory centers (Supplement 1 and Supplement 2). Written informed consent was obtained before study enrollment by patients or legal representatives with subsequent patient assent as possible. An independent data and safety monitoring board (DSMB) oversaw trial conduct and remained blinded to patient assignment. This study followed the Consolidated Standards of Reporting Trials (CONSORT) reporting guidelines.

The first author (A.W.A.) oversaw the project with research staff. The first author and coinvestigators remained blinded to data submitted from other sites throughout trial conduct. Emergency physicians, nurses, neurology residents, endovascular laboratory personnel, and neurointerventionalists were not informed of study and patient enrollment. Statistical analyses were conducted by 2 investigators (P.M., G.T.A.) from centers external to trial enrollment who managed the study database and performed planned interim and final analyses. Protocol fidelity was monitored by 2 investigators (S.M., B.B.B.) external to trial enrollment who attest to the internal validity of project methods and blinded/naive end point collection. Neuroimaging was deidentified and routed from each site to an external core laboratory for blinded interpretation by a single nonenrolling investigator (D.S.L.); core laboratory results were routed to external statisticians for categorization by random assignment and analysis. One amendment was added to the protocol (eTable 1 in Supplement 3).

### Patients

Eligible patients with ischemic stroke were 18 years or older with baseline head CT negative for hemorrhage or mass effect, an Alberta Stroke Program Early Computed Tomography Score (ASPECTS) of 6 or higher for anterior circulation stroke, LVO on CT angiography (CTA), and a prestroke baseline modified Rankin Score (mRS) of 0 to 1 point. ASPECTS was not recorded in patients with posterior circulation stroke. The original protocol allowed enrollment of patients within 6 hours of symptom onset; this was amended, allowing enrollment of patients with ASPECTS of 6 or higher and/or favorable CT perfusion per local standard up to 24 hours from symptom onset (eTable 1 in Supplement 3). Systemic thrombolysis (intravenous tissue plasminogen activator [IVtPA]) was allowed, but enrollment was discouraged when IVtPA was given more than 15 minutes before randomization to prevent confounding of intervention effects on clinical change. Vomiting before consent was an exclusion to prevent confounding of protocol safety. Emergent intubation was an exclusion; however, thrombectomy procedural intubation was allowed. Patients with conditions challenging tolerance of 0° head positioning were evaluated on a case-by-case basis for inclusion. Chest radiography was not required; however, patients with known pneumonia, pleural effusion, pulmonary edema, or concerning breath sounds were excluded to prevent confounding of protocol safety.

Patient race and ethnicity data were included in this study to show generalizability of findings across different racial or ethnic groups. Patients self-reported the following races: Asian, Black, Hawaiian or Other Pacific Islander, multiracial, Native American or Alaska Native, and White.

### Randomization and Treatment

All patients underwent standard of care CT and CTA with or without CT perfusion per local standard. Consent processes were initiated on confirmation of favorable parenchymal imaging and LVO. Patients meeting standard of care criteria received IVtPA with care taken not to delay treatment. A baseline National Institutes of Health Stroke Scale (NIHSS) score was obtained after neuroimaging completion while patients in both groups were at a 0° position on the CT scanning bed; this NIHSS score served as the time 0 starting NIHSS score.

Patients were randomized 1:1 to 0° or 30° head positioning and positioned using height-graded electrical or hydraulic stretchers. Investigators stayed with patients until thrombectomy commenced to ensure assigned positions were maintained.

### End Points

The primary end point was early neurological deterioration (NIHSS worsening ≥2 points) before thrombectomy. The directional superiority hypothesis was as follows: patients with LVO placed in a 0° head position (*V_1_*) will experience less early neurological deterioration before thrombectomy than those with 30° head elevation (*V_c_*), or *H_0_*: *V_1_* = *V_c_* vs *H_A_*: *V_1 _*< *V_c_*_._

Serial NIHSS scores were obtained every 10 minutes by an NIHSS-certified practitioner naive to the study, patient enrollment, and end points, with the final NIHSS measure scored on arrival to the laboratory or immediately before procedural intubation while the patient remained in the assigned position. Assigned position duration/NIHSS monitoring depended on thrombectomy start time, with last NIHSS scored before moving to the catheterization table. Safety outcomes included severe neurological deterioration (worsening of ≥4 NIHSS points) before thrombectomy, hospital-acquired pneumonia,^25,26^ and all-cause death during the 3-month enrollment period. Serious adverse events were adjudicated by the DSMB.

Prespecified exploratory outcomes included 24-hour NIHSS score, discharge or day 7 NIHSS score, and 3-month telephone mRS. The imaging core laboratory assigned ASPECTS, LVO location, and expanded Thrombolysis in Cerebral Infarction (eTICI) scores. In cases where planned thrombectomy did not occur, the first angiographic run eTICI score was carried forward as the postthrombectomy score.

### Data Collection

Serial NIHSS scores, vital signs, and oxygen saturation were obtained every 10 minutes until thrombectomy started. At 24 hours, the NIHSS score and vital signs were recorded, along with postthrombectomy activity. On discharge or day 7, the NIHSS score, vital signs, and mRS were recorded. At 3 months, a telephone mRS was completed by a certified investigator using previously described validated methods.^27^

### Statistical Analyses

From our pilot work,^4,28^ we expected to see, at most, 5% of patients with 0° head positioning have worsening of the NIHSS score by 2 points or higher, compared with 20% of patients with 30° head positioning. We allowed early stopping due to futility or efficacy based on a 2-sample 2-sided proportion test as implemented in East, version 6.0 (Cytel) with type I error α = .05 and 80% power; it incorporated 3 preplanned interim analyses and 1 final analysis of data. The Lan-Demets α and β spending approach with O’Brien-Fleming boundaries^29^ were used both for futility and efficacy interim monitoring. Absence of primary end point evaluation was counted as worsening of the NIHSS score by 2 points or higher, allowing for an intention-to-treat analysis with no primary end point attrition. We applied sequential testing in both patient groups at each interim analysis. The first interim analysis was performed (*z* value intervals >4.333 or <−4.333 = efficacy; *z* value intervals >−0.007 or <0.007 = futility), and the second interim analysis was performed (*z* value intervals >2.963 or <−2.963 = efficacy; *z* value intervals >−0.374 or <0.374 = futility). A third interim analysis was planned but not conducted due to protocol termination by the DSMB.

We used unadjusted Cox proportional hazards modeling to compare and Kaplan-Meier curves to visualize at time 0 the rate of change greater than or equal to 2 NIHSS points and the rate of change greater than or equal to 4 NIHSS points. We included NIHSS score at time 0 and site-adjusted models for worsening of the NIHSS score by 2 or 4 points or higher, although these were not prespecified in the statistical analysis plan. A linear regression model was used to evaluate the association between the treatment group and the change in NIHSS while adjusting for the NIHSS score at time 0. Secondary and exploratory outcomes were compared between groups using Fisher Exact test with odds ratios (ORs) and corresponding 95% CIs and *P* values from unadjusted logistic regression models with randomization assignment as the predictor. Exploratory 90-day mRS was analyzed as follows: dichotomized (mRS 0-2 vs 3-6) and modeled as a dependent variable by unadjusted logistic regression, analyzed as separate categories and modeled by ordinal logistic regression where verification of the proportional odds assumption was done by likelihood ratio testing, and transformed to a utility-weighted mRS (UW-mRS) where each of the mRS categories (from 0-6) was assigned a weight (1.00, 0.91, 0.76, 0.65, 0.33, 0.00, and 0.00)^30^ and modeled by unadjusted linear regression. Carry-forward imputation was only used for eTICI scores in patients who did not undergo thrombectomy after angiography. All statistical analyses were performed using R, version 4.3.1 (R Foundation for Statistical Computing).

## Results

### Patients

**Figure 1.**
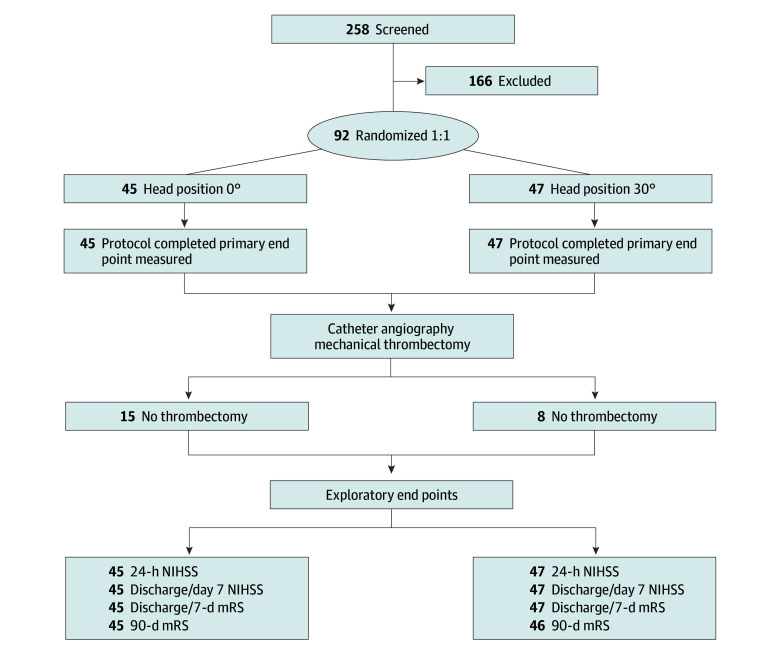
Study Consolidated Standards of Reporting Trials (CONSORT) Diagram Primary end point measured in 100% of enrolled individuals. Exploratory 3-month modified Rankin Scale (mRS) score end point had 1 patient lost to follow-up in the 30° group. NIHSS indicates National Institutes of Health Stroke Scale.

Planned enrollment included 182 patients. Between May 23, 2018, and November 1, 2023, when the DSMB halted enrollment, a total of 92 patients (mean [SD] age, 66.6 [14.4] years; 44 female [47.8%]; 48 male [52.2%]) were enrolled and randomized to either a 0° or 30° head position until DSMB study closure at the second interim analysis. Patients self-reported the following races: 2 Asian (2.2%), 38 Black (52.2%), 1 Native American or Alaska Native (1.1%), and 51 White (55.4%). The trial was conducted at 12 hospitals across the continental US (10 certified comprehensive and 2 certified thrombectomy-capable hospitals). Beginning April 2020 through November 2021, screening and enrollment were placed on hold at 8 sites and were reduced at the 4 remaining sites due to the COVID-19 pandemic. There was no imbalance by site of enrollment. Forty-six patients were randomized to 0° head positioning; however, 1 patient, for whom consent was provided by a relative, was removed from the study when assent was withdrawn 24 hours after thrombectomy, leaving 45 patients in the 0° arm. In the 30° arm, 47 patients were enrolled and randomized (Figure 1). Forty-four patients with disabilities could not be consented because no legal representative was available, 51 were excluded with IVtPA greater than 15 minutes, and 6 declined participation.

Fidelity analyses found all patients to be positioned correctly in assigned head positions, with all primary end point NIHSS assessments performed while head position was maintained as assigned before placement on the catheterization table. One patient with 0° head positioning attempted to pull himself up as his weakness improved and was out of position for a reported 15 seconds when the investigator intervened with repositioning. One patient with 30° head positioning had the NIHSS score at time 0 taken with the head positioned at 30° instead of 0°. No other protocol fidelity issues were identified.

On hospital arrival, 77.2% of subsequently enrolled patients (71 of 92 patients) were received from transport teams with their heads positioned at 30°. Baseline patient characteristics were similar in both groups (Table 1). A total of 90 of 92 patients (97.8%) had a prestroke mRS score of 0 to 1. Intravenous thrombolysis before thrombectomy was administered in 23 of 45 patients (51.1%) with a 0° head position and 24 of 47 patients (51.1%) with a 30° head position. Baseline median ASPECTS were similar at 8 (IQR, 6-8) in patients with a 0° head position and 8 (IQR, 7-8) in patients with a 30° head position. Starting median NIHSS scores at time 0 were similar between both groups, at 10 (IQR, 8-15) in patients with a 0° head position and 10 (IQR, 6-15.5) in patients with a 30° head position (Table 1).

**Table 1.** Patient Baseline Characteristics

Characteristic	Cohort		Overall (N = 92)
	0° (n = 45)	30° (n = 47)	
Age, mean (SD), y	65.27 (14.02)	67.79 (14.85)	66.55 (14.43)
Sex, No. (%)			
Female	20 (44.4)	24 (51.1)	44 (47.8)
Male	25 (55.6)	23 (48.9)	48 (52.2)
Race, No. (%)			
Asian	1 (2.22)	1 (2.13)	2 (2.17)
Black	18 (40.00)	20 (42.55)	38 (41.30)
Hawaiian or Other Pacific Islander	0	0	0
Multiracial	0	0	0
Native American or Alaska Native	0	1 (2.13)	1 (1.10)
White	26 (57.78)	25 (53.19)	51 (55.43)
Arrival or encounter mode, No. (%)			
Ambulance	29 (64.44)	29 (61.70)	58 (63.04)
Hospital transfer	10 (22.22)	10 (21.27)	20 (21.74)
Private auto	4 (8.90)	3 (6.38)	7 (7.61)
Mobile stroke unit	1 (2.22)	2 (4.26)	3 (3.26)
Inpatient stroke alert	1 (2.22)	2 (4.26)	3 (3.26)
Unknown	0	1 (2.13)	1 (1.09)
Head position when first encountered by study team, No. (%)			
0°	11 (24.44)	10 (21.28)	21 (22.83)
30°	34 (75.56)	37 (78.72)	71 (77.17)
Past medical history, No. (%)			
Hypertension	32 (71.11)	38 (80.85)	70 (76.09)
TIA or stroke	9 (20.00)	6 (12.77)	15 (16.30)
Diabetes	10 (22.22)	11 (23.40)	21 (22.83)
Coronary artery disease	4 (8.89)	8 (17.02)	12 (13.04)
Heart failure	6 (13.33)	8 (17.02)	14 (15.22)
Cardiac valvulopathy	1 (2.22)	3 (6.38)	4 (4.35)
Peripheral vascular disease	1 (2.22)	1 (2.13)	2 (2.17)
Carotid atheroma	0	1 (2.13)	1 (1.09)
Carotid revascularization	0	1 (2.13)	1 (1.09)
Thyroid disease	2 (4.44)	2 (4.26)	4 (4.35)
COPD	3 (6.66)	5 (10.64)	8 (8.70)
Active smoker	13 (28.89)	9 (19.15)	22 (23.91)
Cancer	8 (17.78)	5 (10.64)	13 (14.13)
Sleep disordered breathing	4 (8.89)	1 (2.13)	5 (5.43)
Alcohol abuse	4 (8.89)	1 (2.13)	5 (5.43)
SBP, mean (SD), mm Hg	149.53 (30.25)	157.94 (26.06)	153.83 (28.35)
DBP, mean (SD), mm Hg	85.27 (16.60)	89.13 (16.01)	87.24 (16.33)
Glucose, mean (SD), mg/dL	133.99 (74.64)	148.83 (65.82)	141.52 (70.28)
NIHSS score at time 0, median (IQR)	10 (8-15.0)	10 (6-15.5)	10 (7-15.25)
ASPECTS, median (IQR)	8 (6-8)	8 (7-8)	8 (7-8)
Prestroke baseline mRS, No. (%)			
0	34 (75.56)	35 (74.47)	69 (75.00)
1	10 (22.22)	11 (23.40)	21 (22.82)
2	1 (2.22)	0	1 (1.09)
3	0	1 (2.13)	1 (1.09)
Side of occlusion, No. (%)			
Right	28 (62.22)	21 (44.68)	49 (53.26)
Left	16 (35.56)	23 (48.93)	39 (42.39)
Vertebrobasilar	1 (2.22)	2 (4.26)	3 (3.26)
Bilateral hemispheres	0	1 (2.13)	1 (1.09)
Stroke vascular territory, No. (%)			
Middle cerebral artery	37 (82.22)	37 (78.72)	74 (80.43)
Internal carotid artery	6 (13.34)	7 (14.89)	13 (14.14)
Anterior cerebral artery	0	0	0
Basilar	1 (2.22)	2 (4.26)	3 (3.26)
Multiterritory	1 (2.22)	1 (2.13)	2 (2.17)
Systemic thrombolysis, No. (%)			
Alteplase	22 (48.89)	22 (46.80)	44 (47.83)
Tenecteplase	1 (2.22)	2 (4.26)	3 (3.26)
Any systemic thrombolytic agent	23 (51.11)	24 (51.06)	47 (51.09)
Head position interrupted after randomization, No. (%)	1 (2.22)	0	1 (1.09)
Stroke mechanism, No. (%)			
Large artery atheroma	13 (28.90)	7 (14.89)	20 (21.74)
Cardioembolism	24 (53.33)	28 (59.57)	52 (56.52)
Stroke of unusual etiology	2 (4.44)	2 (4.26)	4 (4.35)
Cryptogenic	6 (13.33)	9 (19.15)	15 (16.30)
Unknown	0	1 (2.13)	1 (1.09)
Positive COVID-19 diagnosis, No. (%)	1 (2.22)	3 (6.38)	4 (4.35)
Stroke onset to arrival, mean (SD), min	161.61 (179.82)	71.35 (76.07)	119.85 (147.76)
Stroke onset to enrollment [consent], mean (SD), min	221.27 (175.46)	124.57 (85.67)	174.26 (146.61)
Stroke onset to enrollment [consent] in only patients receiving thrombolysis, mean (SD), min	143.30 (84.23)	96.30 (45.99)	119.80 (71.19)
Stroke onset to intervention positioning, mean (SD), min	227.16 (210.08)	125.09 (82.36)	177.54 (168.13)
Arrival to intervention positioning, mean (SD), min	41.66 (24.60)	47.66 (24.45)	44.66 (24.57)
Stroke onset to thrombectomy, mean (SD), h	4.47 (3.55)	2.67 (1.37)	3.59 (2.83)

Abbreviations: ASPECTS, Alberta Stroke Program Early Computed Tomography score; COPD, chronic obstructive pulmonary disease; DBP, diastolic blood pressure; mRS, modified Rankin Scale; NIHSS, National Institutes of Health Stroke scale; SBP, systolic blood pressure; TIA, transient ischemic attack.

SI conversion factor: To convert glucose to millimoles per liter, multiply by 0.0555.

### Intervention Timing

**Figure 2.**
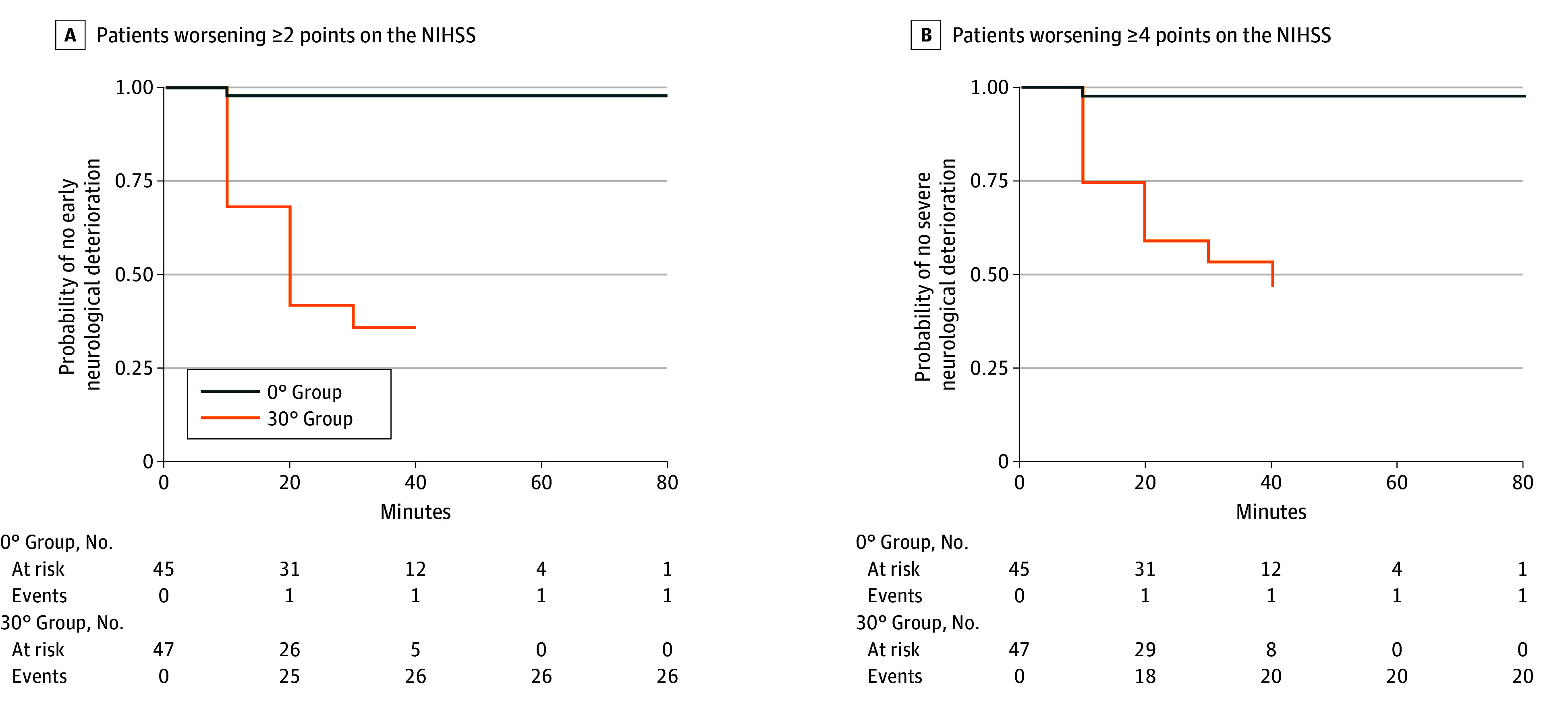
Change in Clinical Stability Over Time A, Kaplan-Meier curves of the patients in the 0° and 30° groups for worsening 2 or more points on the National Institutes of Health Stroke Scale (NIHSS). B, Kaplan-Meier curves of the patients in the 0° and 30° groups for worsening 4 or more points on the NIHSS.

Excluding patients with unknown time of onset, those with a head position at 0° were consented later after developing stroke symptoms than those with a head position at 30° (mean [SD] time, 221.27 [175.46] minutes vs 124.57 [85.67] minutes; 95% CI, 31.96-161.44 minutes); patients with a head position at 0° who underwent thrombolysis were also consented later after developing symptoms than those with a head position at 30° (mean [SD] time, 143.30 [84.23] minutes vs 96.30 [45.99] minutes; 95% CI, 6.33-87.67 minutes). Time from hospital arrival to assigned position was balanced between the groups (Table 1). Head position protocol termination for catheterization table movement was fastest in patients with a head position at 30° (Figure 2), with all patients in this group positioned for thrombectomy by the 40-minute NIHSS assessment. At the direction of the local neurointerventional surgeon, 2 patients in the 30° group never underwent catheter angiography/treatment. At 50 minutes, 7 patients in 0° group had not moved to catheterization, and 4 of them did not undergo catheter angiography/treatment at the direction of the local neurointerventional surgeon.

### Primary Outcome

Overall, worsening NIHSS score of 2 or more points occurred in 1 patient in the 0° group and in 26 patients in the 30° group (hazard ratio [HR], 34.40; 95% CI, 4.65-254.37; *P* < .001) (Table 2); adjusted analyses produced similar results (eTable 2 in Supplement 3). Figure 2A shows Kaplan-Meier curves representing the probability of worsening NIHSS score of 2 or more points for the 2 groups over time (measured in minutes) until protocol termination at the start of catheter angiography. Although both groups had similar NIHSS scores at time 0, over time, patients with a 30° head position experienced greater NIHSS score worsening, whereas those with a 0° head position showed score stability (Figure 2A). After adjusting for NIHSS score at time 0, patients in the 30° group experienced significantly greater NIHSS worsening compared with those in the 0° group (adjusted mean difference, 1.85 points; 95% CI, 1.13-2.58; *P* < .001).

**Table 2.** Primary and Safety Outcomes

Outcome	Cohort		Unadjusted HR/OR (95% CI)	*P* value
	0° (n = 45)	30° (n = 47)		
Primary outcome				
Early neurological deterioration [≥2 points worsening from NIHSS score at time 0], No. of events (person-time in minutes)	1 (1230)	26 (850)	34.40 (4.65-254.37)	<.001
Safety outcomes				
Severe neurological deterioration [≥4 points worsening from NIHSS score at time 0], No. of events (person-time in minutes)	1 (1230)	20 (940)	23.57 (3.16-175.99)	.002
Hospital acquired pneumonia, No./total No. (%)	0	0	NA	NA
Death at discharge/day 7, No./total No. (%)	1/45 (2.22)	1/47 (2.13)	0.96 (0.05-15.78)	.98
90 d All-cause mortality, No./total No. (%)	2/45 (4.44)	10/46 (21.74)	5.81 (1.20-28.22)	.03
Postdischarge stroke, No./total No. (%)	1/45 (2.22)	0	NA	.49
Symptomatic intracerebral hemorrhage, No./total No. (%)	0	1/47 (2.13)	NA	>.99
Prespecified exploratory outcomes, No./total No. (%)				
Improvement at 24 h	39/45 (86.67)	28/47 (59.57)	0.24 (0.08-0.68)	.01
Improvement at discharge/day 7	39/45 (86.67)	31/47 (65.96)	0.32 (0.11-0.92)	.03
90-d mRS 0-1	24/45 (53.33)	21/46 (45.65)	0.74 (0.32-1.68)	.46
90-d mRS 0-2	31/45 (68.89)	26/46 (56.52)	0.59 (0.25-1.39)	.22
90-d mRS, median (IQR)	1 (1-4)	2 (1-5)	1.85 (0.89-3.89)	.10
UW-mRS, mean (SD)	0.71 (0.34)	0.57 (0.42)	NA	.09

Abbreviations: HR, hazard ratio; mRS, modified Rankin Scale; NA, not applicable; NIHSS, National Institutes of Health Stroke Scale; OR, odds ratio; UW-mRS, utility-weighted mRS.

At the discretion of local neurointerventionalists, 15 patients (33.3%) in the 0° group and 8 patients (17.0%) in the 30° group (*P* = .09) did not undergo thrombectomy. Baseline eTICI scores were similar between groups on first angiographic runs (eTable 3 in Supplement 3); similarly, there was no difference in eTICI scores between groups on final postprocedure angiographic runs (eTable 3 in Supplement 3). However, all 30 patients undergoing thrombectomy in the 0° group achieved eTICI 2b50 to 3 scores on the final postprocedure angiographic runs, compared with 34 of 39 patients (87.2%) in the 30° group (*P* = .06).

### Secondary Safety Outcomes

One patient in the 0° group, compared with 20 in the 30° group, had worsening NIHSS score of 4 or more points during positioning (HR, 23.57; 95% CI, 3.16-175.99; *P* = .002) (Table 2 and Figure 2B); adjusted analyses produced similar results (eTable 2 in Supplement 3). No patients developed hospital-acquired pneumonia or any form of pulmonary deterioration during hospitalization. One patient in each group died during hospitalization after failed thrombectomy with evolution of stroke/withdrawal of care. All-cause death over the 90-day study period occurred in 2 patients (4.4%) in the 0° group, compared with 10 patients (21.7%; *P* = .03) in the 30° group (Table 2).

### Exploratory Outcomes

At 24 hours after thrombectomy, 39 of 45 patients (86.7%) in the 0° group had improved from their NIHSS score at time 0, compared with 28 of 47 patients (59.6%) in the 30° group (OR 0.24; 95% CI, 0.08-0.68; *P* = .01) (Table 2). At discharge or day 7, 39 of 45 patients (86.7%) in the 0° group demonstrated sustained improvement from their NIHSS score at time 0, compared with 31 of 47 patients (66.0%) in the 30° group (OR, 0.32; 95% CI, 0.11-0.92, *P* = .03). At 3 months, with 1 patient lost to follow-up, 31 of 45 patients (68.9%) in the 0° group, compared with 26 of 46 patients (56.5%) in the 30° group, had an mRS score less than or equal to 2 (OR, 0.59; 95% CI, 0.25-1.39; *P* = .22; mean [SD] UW-mRS, 0° group, 0.71 [0.34] vs 30° group, 0.57 [0.42]; 95% CI, 0.02-0.30; *P* = .09) (eFigure in Supplement 3).

## Discussion

In patients with LVO with viable penumbral tissue, 0° head positioning resulted in lower rates of worsening NIHSS score of 2 or more points compared with 30° head elevation before thrombectomy. Furthermore, 0° positioning was safe, with fewer deaths and no risk of hospital-acquired pneumonia. Conversely, 30° positioning resulted in high numbers of worsening NIHSS score of 2 or more points.

This was the first study, to our knowledge, to collect serial NIHSS scores in patients with LVO before thrombectomy during head positioning demonstrating the dynamic nature of clinical fluctuation. Although TCD blood flow positional changes supported our pilot work,^3,4,28^ we excluded TCD due to limited generalizability. Instead, we chose a clinical primary end point, the NIHSS score, which is used universally by stroke centers and as an end point in numerous clinical trials. Given thrombectomy’s significant functional outcome effects,^14,15,16,17,18,19,20,21^ we timed primary end point attainment before thrombectomy.

Our primary end point of worsening NIHSS score of 2 or more points is consistent with that of other studies.^28,31,32,33^ The importance of this change is supported by the significant benefit of 0° head positioning on preventing worsening, including worsening NIHSS score of 4 or more points before thrombectomy; furthermore, 0° head positioning improved 24-hour and discharge NIHSS scores. Although our data might be used to calculate effect size to power future trials measuring 3-month outcomes, the need for further studies subjecting patients with LVO to head elevation should be balanced against our findings showing harm.

We posit that 0° head positioning is a bridging maneuver to thrombectomy, protecting ischemic tissue and possibly reducing infarct progression before definitive treatment. Our pilot demonstrated an 18% improvement in clinical findings after 0° head positioning.^4^ More recently, others have shown that intermittent use of −20° head position in patients lacking reperfusion treatment for LVO^34^ or large artery atherosclerotic stroke^9^ may be beneficial. However, no studies, to our knowledge, have attempted to further the impact of thrombectomy on 3-month outcomes using head positioning. Regardless, ensuring clinical stability and brain parenchymal viability before thrombectomy is important, especially given that delays to thrombectomy treatment may produce worse postprocedural outcomes.^24,25^

Our findings may be important for future LVO trials enabling standardized head positioning before thrombectomy. We have shown previously^35^ and in this trial that 0° positioning is low risk, favoring arguments for widespread adoption to minimize neurologic deterioration before thrombectomy. Because most patients arrive with heads elevated, training of emergency personnel, helicopter, and ground transport teams should accompany implementation to ensure compliance with this simple, protective maneuver.

### Limitations

Our study has limitations. First, the small sample size must be considered; however, we powered our study from pilots demonstrating a large effect size for clinical improvement, and because our pilot work was completed prior to current penumbral viability confirmation methods, it is not surprising that patients with a 0° head position experienced an even greater effect. Second, we used study and outcome-blinded practitioners to collect NIHSS measures because disguising patient head position was impossible. Prospective randomized outcome blinded evaluation (PROBE) designs were used for several key endovascular trials,^14,15,16,17,18,20,21^ and similar to our methods, these studies could also be criticized for bias risk. However, our fidelity analyses found that practitioners scoring the NIHSS and other key personnel were unaware of the study. This was likely supported by the hectic emergency prethrombectomy environment, as well as lack of consensus among practitioners about best head position. Third, because this was not a thrombectomy study, we did not interrogate interventionalists about treatment decisions to avoid contamination of blinding; however, clinical improvement may have played a role in decisions not to treat patients with a 0° head position. Lastly, we did not enroll patients transferred between facilities, as our pilot observations showed that even when ordered to transport at 0°, patients arrived with heads elevated to accommodate cardiac monitors. Had these patients been enrolled and guaranteed head position fidelity, we may have seen greater differences in 3-month outcomes because of interfacility transfer delays.

## Conclusions

In this randomized clinical trial, 0° head positioning for patients with acute LVO and viable brain parenchyma was protective, maintaining clinical stability before thrombectomy. Future implementation studies should explore fidelity and outcomes of 0° positioning, particularly in patients requiring interfacility transfer.

## Notes

Supplement 1.
Trial Protocol.

Supplement 2.
Statistical Analysis Plan.

Supplement 3. **eTable 1.** Protocol Amendment **eTable 2.** Adjusted Hazard Ratios for the Primary Outcome >2 Points NIHSS Worsening (END) and the Safety End Point >4 Points NIHSS Worsening (SND) **eTable 3.** Thrombectomy Treatment Characteristics **eFigure.** Exploratory 90-Day Modified Rankin Score (mRS) Analyses

Supplement 4. **Nonauthor Collaborators.** ZODIAC Investigators.

Supplement 5.
Data Sharing Statement.
