# Supplementary material for: Optimal Head-of-Bed Positioning Before Thrombectomy in Large Vessel Occlusion Stroke: A Randomized Clinical Trial
Source: JAMA Neurol. 2025 Jun 4;82(9):905–14. doi: 10.1001/jamaneurol.2025.2253 (PMC12138796; doi:10.1001/jamaneurol.2025.2253)
Supplement: Supplement 1. — Trial Protocol. [file jamaneurol-e252253-s001.pdf]

ZODIAC PROTOCOL (version 1.0)

Protocol Title: Zero Degree Head Positioning in Hyperacute Large Artery Ischemic Stroke (ZODIAC)

Trial registration: Pending grant funding

Sponsor:

University of Tennessee

Pending Grant Funding:

1R01NR017850-01

Principal Investigator: Anne W. Alexandrov **Date:** May 10, 2018

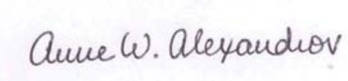

TABLE OF CONTENTS

Version 1.0

Specific Aims	3
Research Protocol	
Site Eligibility	4
Inclusion/Exclusion Criteria	4
Blinding	5
Subject Screening, Enrollment, and Serial Monitoring	
Standard of Care	5
Enrollment and Serial Monitoring	6
Decision Not to Treat	6
Post Mechanical Thrombectomy Care	6
Standard of Care Neuroimaging	6
Protocol Fidelity	7
Randomization and Power/Sample Size	7
Endpoints	9
Analysis Plan for Aim 1	10
Analysis Plan for Process Evaluation	10
Analysis Plan for Aim 2	10
Pre-specified Exploratory Endpoints	11
Data Safety Monitoring Plan	13
References	21

Version 2.0

Specific Aims	25
Research Protocol	26
Site Eligibility	26
Inclusion/Exclusion Criteria	26
Blinding	27
Subject Screening, Enrollment, and Serial Monitoring	
Standard of Care	27
Enrollment and Serial Monitoring	28
Decision Not to Treat	28
Post Mechanical Thrombectomy Care	28
Standard of Care Neuroimaging	28
Protocol Fidelity	29
Randomization and Power/Sample Size	29
Endpoints	31
Analysis Plan for Aim 1	32
Analysis Plan for Process Evaluation	32
Analysis Plan for Aim 2	32
Pre-specified Exploratory Endpoints	33
Data Safety Monitoring Plan	35
References	43

Summary of Protocol Changes 46

SPECIFIC AIMS

Positioning of the patient with hyperacute large artery ischemic stroke is an important, yet understudied aspect of care that could impact the course of treatment and ultimately clinical outcome for this most severe type of stroke patient. Positioning with the head of bed (HOB) at 0° has been shown in small studies to increase blood flow across points of arterial stenosis in hyperacute large artery ischemic stroke, leading to clinical improvement in stroke symptoms.[1-5] Since 1968, small studies have also documented clinical symptom worsening in ischemic stroke patients when the HOB has been elevated to 30° or higher.[6-10] A recent large cluster randomized trial (HeadPoST) attempted to determine what head position was best in stroke, but failed to enroll the patient cohort for which 0° head positioning has been shown to benefit.[11] Findings from HeadPoST have been highly criticized by the leadership of all international stroke societies due to significant design flaws, enrollment of a subacute small vessel and intracerebral hemorrhage sample for whom the damage of stroke had already occurred, and questionable intervention fidelity.[12-15] In fact, HeadPoST has become a catalyst for a now urgent call to conduct high quality head positioning research in hyperacute large artery ischemic strokes to definitively answer the question of how best to manage these highly vulnerable patients to ensure stability and prevent symptom worsening.[12]

Early neurologic deterioration is common in hyperacute ischemic stroke, ranging from 15%[16] to 37.5%[17] in large clinical trials, and selection of HOB positioning may play an important role in its occurrence. However, to date, clinical trial protocols exploring numerous drug and device interventions have remained silent on HOB positioning. Mechanisms proposed for clinical improvement at 0°-HOB include favorable gravitational arterial flow conditions,[2,3] recruitment of collateral arterial blood flow routes,[2-4] and in the case of intravenous thrombolysis, improved clot-lytic interactions augmenting arterial recanalization.[2] Collectively, these mechanisms have caused many to argue that 0°-HOB positioning should be among the first steps taken in large artery hyperacute ischemic stroke patient management. The goal of this efficacy study is to determine if 0°-HOB positioning in hyperacute ischemic stroke prevents neurological symptom worsening in large artery occlusion patients. The study will achieve these goals through use of a novel protocol enabling enrollment of consecutive large artery occlusion patients while maintaining compliance with management specified by national guidelines,[18-20] stroke center certification requirements,[21] and U.S. core measures.[18-21] We will enroll hyperacute large artery patients with salvageable brain early into stroke symptoms, that are candidates for mechanical thrombectomy (MT) (n=182). Patients will be randomized to one of two groups: 1) 0°-HOB positioning; or, 2) 30° HOB positioning. The primary endpoint will be early neurological deterioration (stroke symptom worsening) defined as an increase of 2 or more points from baseline (pre-intervention) National Institutes of Health Stroke Scale (NIHSS) score during the positioning period. These data will be employed to meet the following specific aims:

<u>Aim 1 (primary efficacy endpoint):</u> To identify if use of 0° HOB positioning is associated with clinical stability in hyperacute ischemic stroke. <u>*Hypothesis*</u>: *Patients with large artery occlusions placed in a 0° HOB position (V_1), (superiority hypothesis), will experience less early neurologic deterioration within the time prior to thrombectomy, than those in the 30° HOB elevation group (V_c)*, or H_0: $V_1 = V_c$ <u>versus</u> H_A: $V_1 < V_c$.

Aim 1 will be supported by a process evaluation plan that identifies key components (context; reach and recruitment; implementation dose delivered and received; and, overall fidelity), related questions, and associated process stability indicators.[22-26] Implementation methods are proposed to support, continuously monitor, report, and improve process stability and overall protocol fidelity.

<u>Aim 2 (secondary aim assessing safety):</u> To confirm the safety of 0°-HOB positioning in a large, generalizable sample of hyperacute large artery ischemic stroke patients.

RESEARCH PROTOCOL

This phase III study will utilize a multicenter, prospective randomized outcome-blinded evaluation (PROBE) approach enrolling consecutive hyperacute large artery ischemic stroke patients to determine if use of 0°-head positioning is associated with greater clinical stability than 30°-head positioning. PROBE designs are among the most well-respected design options within the stroke scientific community when blinding of both subjects and enrolling clinicians is impossible and have been used to support all recent large artery ischemic stroke clinical trials.[22-27]

Site Eligibility. Sites eligible for participation in ZODIAC must meet the following criteria:

- Research infrastructure to support a clinical trial
- Certification as a comprehensive stroke center or thrombectomy-capable stroke center
- Rapid response stroke team capable of STAT emergency in-person presence at the time of acute stroke arrival
- Stroke team leadership by fellowship trained vascular neurologists and neurovascular fellowship trained ANVP board certified advanced practice providers
- Rapid response stroke team certification in both the NIHSS and the modified Rankin Score (mRS); team member inter-rater reliability of scores consistently between 97-100% for both NIHSS and mRS
- Compliance with stroke quality core measures consistently documented at $\geq$ 90% over the previous 6 months

Inclusion and Exclusion Criteria. Adult ($\geq$18 years) hyperacute ischemic stroke patients will be screened for enrollment. Because large artery ischemic stroke is not unique to any one sex, we will enroll both women and men.

Inclusion Criteria-

- Ischemic stroke symptoms consistent with large vessel occlusion
- Baseline standard of care non-contrast head CT (or MRI) negative for hemorrhage or mass-effect
- Evidence of arterial occlusion on standard of care CT angiography or MR angiography
- Favorable neuroimaging (Alberta Stroke Program Early Computed Tomography Score [ASPECTS] $\geq$ 6 in anterior circulation stroke; not applicable in posterior circulation stroke)
- Anticipated treatment with mechanical thrombectomy
- Pre-stroke baseline modified Rankin Score (mRS) $\leq$ 1
- Symptom onset within 6-hours of start time for catheter angiography/ planned thrombectomy procedure

Exclusion Criteria-

- Pregnancy or suspicion of pregnancy
- Evidence or suspicion of vomiting any time prior to consent which could predispose to aspiration pneumonia and therefore confound determination of protocol safety
- Anticipated palliative care referral
- Evidence of evolving malignant infarction on admission noncontrast CT (or MRI)
- Need for emergent intubation with mechanical ventilation, or non-invasive ventilatory support with either bi-level positive airway pressure (BiPAP) or continuous positive airway pressure (CPAP) due to pending or actual respiratory failure prior to or at the time of emergency department

admission. (Note: Elective intubation for the thrombectomy procedure is not an exclusion criterion.)

- Inability to tolerate zero-degree positioning due to congestive heart failure, preexisting pneumonia, chronic obstructive pulmonary disease, or other medical condition. (Note: A diagnosis of heart failure or chronic obstructive pulmonary disease does not automatically exclude enrollment; each patient should be assessed individually for positional intolerance.)
- Admission chest radiograph positive for pleural effusion, pulmonary edema, pneumonia, or other pulmonary condition that may confound determination of protocol safety. (Note: An admission chest x-ray is not required, but may be obtained in patients with concerning pulmonary findings.)
- Abnormal breath sounds on admission assessment that may confound determination of protocol safety
- Lack of a telephone and/or permanent address predisposing patients to be lost to follow up
- Enrollment in another clinical trial that may affect our primary or secondary endpoints
- In the absence of a consenting legal next of kin, any medical, psychological, cognitive, social or legal condition that would interfere with informed consent and/or capacity to comply with all study requirements, including the necessary time commitment
- Note: Enrollment of patients receiving systemic thrombolysis more than 15 minutes prior to randomization is discouraged as this may confound ability to understand the impact of head positioning on clinical stability.

Blinding. Site principal and co-investigators will hold ZODIAC aims, methods, and enrollment in confidence throughout conduct of the trial from other members of the stroke team working in the emergency department, catheterization lab, and stroke unit, including stroke coordinators, rapid response team members, emergency physicians and nurses, neurointerventionalist physicians, and physician residents. Because emergency stroke care is delivered in a hectic fast-paced environment where each responding team member is consumed with their own specific responsibilities, our pilot work has demonstrated that masking of enrollment procedures is easily accomplished. Additionally, because head positioning is often overlooked and commonly varies between providers and patient conditions, pilots of this protocol have shown that practitioners do not notice that some patients are positioned with the head up, and others are positioned with the head down. Therefore, blinded serial measurement of the NIHSS can be accomplished using protocol naïve certified practitioners for collection of endpoints.

Subject Screening, Enrollment, and Serial Monitoring (Figure)

Standard of Care: Suspected acute stroke patients will undergo guideline-supported standard of care stroke team emergency response procedures, including assessment/stabilization of airway, breathing, circulation, placement of a continuous pulse oximetry sensor, with immediate transport for imaging. The admission NIHSS will be scored on route to the imaging suite while the patient is in whatever position prehospital personnel have selected (this assessment is not utilized in the ZODIAC protocol but is a stroke center certification agency requirement). Continuous portable ECG monitoring with nasal cannula oxygen (if necessary, based on pulse oximetry) will be established in CT (or MRI), and IV access will be obtained with STAT blood draw; point of care testing will be conducted immediately before STAT non-contrast CT (or MRI) with CTA (or MRA). A rapid/detailed history/physical exam will be completed concurrently and patients eligible for treatment with tissue plasminogen activator (tPA) will have drug administered in CT (or MRI).

<u>*Enrollment and Serial Monitoring*</u>*:* Subjects meeting inclusions without exclusions will be consented and randomized. All subjects (0-degree and 30-degree) will be maintained at 0-degrees immediately after completion of neuroimaging. An NIHSS-certified practitioner naïve to the research protocol will measure the baseline/time 0 NIHSS score while the patient is at 0-degrees. The local investigator will then position the patient in the randomly assigned position and the serial NIHSS (every 10 minutes) monitoring phase of the intervention will begin. The site investigator must stay with the patient until positioned on the catheterization lab table to ensure assigned head position is maintained. The serial NIHSS monitoring phase ends immediately prior to placement on the catheterization lab table, with the primary endpoint measured during handoff by the same protocol naïve stroke team member to a protocol naïve catheterization lab practitioner certified in the NIHSS; in the case of elective procedural intubation, serial NIHSS monitoring will end immediately preceding the time of induction/intubation for thrombectomy. Of note, throughout execution of this protocol investigators must ensure that procedures do not delay time to tPA or time to catheterization for mechanical thrombectomy.

<u>*Decision Not to Treat*</u>: Should a decision not to perform catheter angiography/mechanical thrombectomy occur at the direction of the local neurointerventional surgeon, enrolled patients will complete the positioning intervention phase of the protocol preferably after the time 8 serial NIHSS assessment or earlier if deemed necessary by the attending physician managing the patient. The case report form should be clearly marked with the time of protocol completion, with all NIHSS scores documented out to this terminal time point.

<u>*Post-MT Standard of Care*</u>: Following the thrombectomy, patients will resume standard of care management for post-thrombectomy patients and can assume whatever head position the stroke team deems appropriate. Standard of care measures typically include serial post-procedural neurological assessments with the NIHSS and strict blood pressure monitoring, however these data will not be analyzed under the ZODIAC protocol. Other standard procedures include obtaining an MRI and other work-up to determine stroke mechanism with assignment of TOAST classification, swallow assessment and advancing diet, application of complication avoidance measures such as venous thromboembolism prophylaxis, and after 24 hours, mobilization procedures. The ZODIAC protocol includes an exploratory 24-hour NIHSS score and a 7 day/discharge NIHSS exploratory assessment by a protocol naïve practitioner certified in the NIHSS. A telephone 3-month mRS exploratory outcome will also be obtained as a standard of care requirement per stroke center certification agencies by a protocol naïve certified practitioner. <u>Note</u>: In enrolled patients not undergoing catheter angiography/mechanical thrombectomy at the direction of the local neurointerventional surgeon, standard of care management will commence once the serial NIHSS monitoring phase of the protocol is complete as described above.

<u>*Standard of Care Neuroimaging*</u>: All routine imaging (e.g. CT, CTA, MRI, MRA, CXR) occurring throughout the hospitalization must be fully de-identified, blinded to group assignment, copied onto CD, and couriered to the UCLA Core Lab for analysis and archiving independent from the clinical data.

Thrombectomy patients are extremely likely to benefit from 0° positioning because the procedure is only performed in patients with viable yet vulnerable penumbral brain tissue. ZODIAC's novel protocol will capture serial NIHSS over the period before thrombectomy begins, allowing us to better understand how to maintain stability in this high-risk vulnerable population.

Figure: Study Protocol

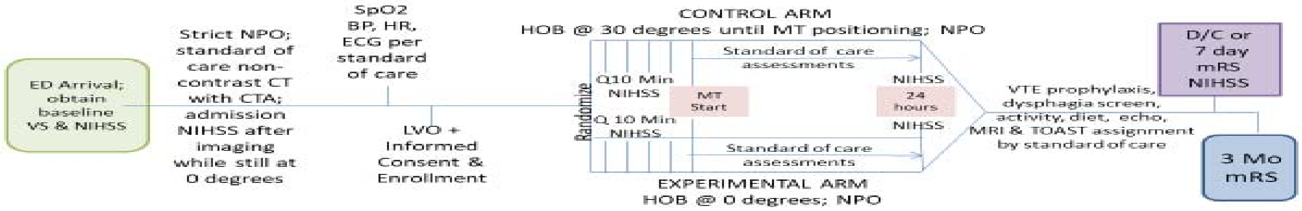

Protocol Fidelity: Table 1 describes the elements of our process evaluation plan.[28-32] This protocol is supported by use of sites led by both fellowship trained vascular neurologists and neurovascular fellowship trained advanced practice nurses that have attained ANVP board certification; collectively, these individuals will oversee the day-to-day management of the protocol. Training manuals and protocol laminated pocket cards include the full protocol, IRB approved/stamped consents, FAQ, troubleshooting, sample case report forms, and PI contact numbers. Site training includes orientation, virtual national meetings, and a 3-month staged pilot protocol implementation with performance feedback/dialogue which will remain ongoing as sites "go live." Data entry hard-stops and text alerts will notify users of documentation errors, and prohibits enrollment of subjects failing to meet inclusion/exclusion criteria. The coordinating center's research coordinator will serve as the clinical site monitor for the trial, independently assessing site performance, protocol compliance, and data entry; reports will be used to generate individual site performance improvement plans as necessary. Study newsletters will disseminate best practices that emerge, share enrollment milestones and highlight sites/providers.

Randomization and power/sample size. Our protocol utilizes two groups, 0°-HOB and 30°-HOB, and allows us to offer enrollment to consecutive eligible patients. To assure balance in the treatment group throughout the course of enrollment, we will use block randomization with a block size of 4 and an allocation ratio of 1:1. This scheme has been implemented in the *Unity* computerized enrollment procedure that only shows the random assignment for a single given participant to the user and may scramble subject enrollment numbers to ensure randomization balance. Future assignments cannot be predicted by study personnel because of the block assignment made together with assignments at all sites, and this effectively mitigates the risk for selection bias that might occur if anticipated group assignment were able to influence enrollment decisions.[33]

Table 1: Process Evaluation Components, Questions, and Indicators[22-26]

Component	Questions	Indicators
Context	Has the standard of care changed to influence adherence or surveillance?<br>Have changes occurred that affect organizational capacity for performance?<br>Have users been exposed to biased messaging that may influence recruitment and/or protocol adherence?	• Consistency of guidelines<br>• CRF structural consistency:<br>o Staff & unit management consistency; staff ratios<br>• Change in screening/enrollment<br>• Feedback from sites
Reach & Recruitment	To what extent is the program reaching intended subjects?<br><br>Are unintended groups enrolled?	• Screening records<br>• Inclusion/exclusion compliance<br>• Differences in intention-to-treat vs. per-protocol treatment of enrolled patients
Implementation:<br><br>Dose Delivered<br><br>Dose Received	To what extent is the program implemented and received?<br><br>How much of the protocol is being administered?<br>What – if anything – is omitted or performed inconsistently?<br><br>What is the average dose received?	• CRF process consistency: Positioning assignment and maintenance; serial monitoring; and, 24-hour, discharge (or 7-day) and 90-day assessments<br>• Neuroimaging enrollment fidelity<br>• Protocol leader oversight records<br>• Feedback from sites
Overall Fidelity (Goal > 90%)	How well does project execution maintain fidelity of the original design?<br>- Aggregate structural compliance<br>- Aggregate process compliance	• CRF structural consistency<br>• CRF process consistency<br>• Blinding of endpoint measurers

We selected individual randomization as opposed to cluster randomization on the hospital/site level for three reasons. First, as emphasized by Murray, "the single most important factor in the power of a group-randomized trial is usually the number of groups."[34] Had we chosen cluster randomization, our sample size would need to increase from n=182, to <u>n=5800</u>, and this would demand <u>at least 29 study sites</u> to efficiently complete the study within 5 years. Given that the largest individually randomized thrombectomy trial included a maximum of 500 patients, and took over 3 years to complete at 16 sites,[32] the conduct of a cluster randomized trial with an even larger thrombectomy sample would require considerable manpower and monetary support, much greater than can be provided. In addition, our 12 geographically widespread sites contribute diverse patients while delivering a consistent standard of care strictly managed by national guidelines, stroke center certification, and core measure requirements.[18-21] Subject diversity is problematic in a cluster randomized design due to an inability to balance important patient traits. In trials involving a small number of clusters (≤10 pairs), the loss of degrees of freedom resulting from pair matching becomes a particularly critical factor.[35] Detailed investigation of this problem has led to the conclusion that for studies with a small number of clusters it is unlikely that effective matching can be possible and that "matching may be overused as a design tool.[36] In contrast, our use of individual (block) randomization will effectively balance patient traits and tPA treatment across protocols, considerably strengthening the evidence derived from this trial. Furthermore, if randomization would be determined on the hospital level, the attending physicians and nurses would know the protocol that would be applied to a patient before that patient is asked to give informed consent. Thus, group-randomization would open our study to enrollment selection bias due to differentially approaching patients that are deemed more/less likely to benefit from the pre-determined protocol for each hospital. This may, in fact, have contributed to enrollment of only small vessel strokes in HeadPoST.[11] Group randomization would also challenge NIHSS/mRS score blinding. In summary,

cluster randomization would be a poor choice due to significant cost, the need for many more enrollment sites, and the oversight manpower required for optimal trial conduct, whereas use of individual randomization provides an economic, feasible and impactful approach to this study.

Data Capture and Control. The UTHSC Center for Biomedical Informatics (CBMI) provides the *RedCap* customized biomedical clinical-research informatics application for data entry. *RedCap* is a fully customizable integrated web-service for data collection with project alerts linked to an encrypted PostgreSQL database and is accessed via the Internet though secure web-applications. Site PIs are required to undergo UTHSC security clearance and training to receive a unique UTHSC ID for access to the *RedCap* system; each site PI will only be able to access data entered directly by them from their site. The study's clinical monitor will verify all data entered from each site against case report forms and electronic health record source data for accuracy; site investigators will be expected to resolve queries about missing data or suspected inaccuracies within 72 hours of notification. At each pre-specified interim analysis milestone, and at the time of the final analysis, research data will be bulk-exported to the ZODIAC biostatisticians who are fully independent from clinical trial enrollment. The national PI will not have access to the study database at any time during or after completion of the trial until an agreed upon post-publication time point.

UTHSC network and computing facilities are owned and managed by UTHSC-ITS; all CBMI systems are housed in the ITS computer center, which has electric power conditioning, UPS battery backup for short-term outages, and a backup diesel generator for long-term outages. The computer center also has fire suppression, temperature and humidity control, card key controlled access and video monitoring. Network traffic crossing the UTHSC network boundary is examined by a Cisco firewall against its Access Control Lists.

Neuroimaging files will be analyzed and archived by the UCLA Neuroimaging Core Lab under the direction of Dr. David Liebeskind. Dr. Liebeskind and his staff will remain fully blinded to patient group assignment; the UCLA team will be responsible for sending all data directly to the ZODIAC statisticians for analysis. While ZODIAC does not incorporate formal imaging-based endpoints, imaging analyses will support protocol fidelity assessment (patient enrollments were consistent with inclusion/exclusion criteria) and will also aid in understanding differences found between groups once the study is complete.

Endpoints.

Primary Endpoint: The percentage of patients with early neurologic deterioration (END) is our primary outcome. END is defined as a two or more point worsening (≥ 2) in the NIHSS score in comparison to the time-0 NIHSS score at the time of positioning intervention termination (placement of the patient on the catheterization table or immediately prior to the time of elective induction/intubation for thrombectomy in centers utilizing general anesthesia). Not deteriorating (being stable) is defined as less than 2-point NIHSS worsening (< 2) or NIHSS score improvement in comparison to the time-0 NIHSS score during the head position intervention.

We expect to see at most 5% of END participants in the 0° head position group, compared to 20% of END patients in the 30° head position group (15% lower deterioration). Our group-sequential design allows for early stopping due to futility or efficacy and is based on a two-sample two-sided proportion test as implemented in East 6.0 (Cytel, Cambridge, MA) with type I error alpha=0.05 and 80% power; it incorporates 3 interim analyses and 1 final look at the data which results in a sample size of n = 182. Interim analyses are planned with equal spacing after cases have been enrolled/observed for the primary endpoint (Table 2). The Lan-Demets alpha and beta spending approach with O'Brien-Fleming

boundaries[37] are used both for futility and efficacy interim monitoring. Absence of primary endpoint evaluation will be counted as an END event so that all randomized participants will have a determined primary endpoint available for analysis, allowing for an intent-to-treat analysis with no primary endpoint attrition.

<u>Analysis Plan for Aim 1 (primary efficacy endpoint); *Hypothesis*</u>: *Patients with large artery occlusions placed in a 0°-HOB position will experience less early neurological deterioration than those in the 30°-HOB elevation group.* We will apply sequential testing in both patient groups (Table 2). As an example, the first look in the protocol is performed when 46 participants are enrolled/have their primary outcome evaluated; if the z-value associated with the test of equality of proportions in both arms is above 4.333 or below -4.333 the trial will be stopped early for efficacy (one group is clearly superior with respect to the primary outcome), whereas if that z-value is in the interval (-0.007, 0.007), the trial will be stopped early for futility (groups are essentially identical in outcome). If the z-value falls within the intervals (-4.333, -0.007) or (0.007, 4.333), the trial will continue until the next look at the data. Note that for the primary endpoint, absence of evaluation is counted as deterioration and that, consequently, all randomized participants will have a determined primary endpoint available for the analysis; therefore, intent-to-treat principles will support our primary data analysis. We will use unadjusted Cox proportional-hazards modeling to compare and Kaplan-Meier curves to visualize the rate of early neurological deterioration (> 2 NIHSS points change from baseline) in the two groups. The number at risk and censored in each group will be reported in the Kaplan-Meier survival curves every 10 minutes. Overall rates of END and according to group will be reported as frequencies and percentages and compared between groups with Fisher's Exact test. The number needed to harm will be estimated as the inverse of the absolute risk increase (ARI) = 1/ARI.

Table 2: Stopping Boundaries for the Sequential Design.

	Sample Size	Stopping Boundaries (Extended Protocol)			
Look #	MT Subjects	Efficacy Z (lower, upper)		Futility Z (lower, upper)	
1	46	4.333	-4.333	0.007	-0.007
2	92	2.963	-2.963	0.374	-0.374
3	138	2.359	-2.359	1.261	-1.261
4	182	2.014	-2.014	2.014	-2.014

<u>Analysis Plan for Process Evaluation</u>. Most of the process evaluation components (Table 1) naturally render themselves to description and categorization. Noteworthy exceptions are aspects relating to intent-to-treat vs. per-protocol received treatment, and control of head positioning assignment. The data collected will enable generation of statistical quality control charts[38-39] if necessary, to monitor the proportion of non-conforming patients (delivered treatment different from assigned protocol) over time. Should control charting be necessary, they will be assembled by ZODIAC independent biostatisticians and provided to Drs. Brewer and Middleton (the study's protocol fidelity and implementation science experts) as an ongoing monitoring effort. The upper and lower control levels of these charts will allow a ready assessment of whether deviations over time or between hospitals are coincidental or driven by some real difference or shift in hospital procedures.

<u>Analysis Plan for Aim 2 (secondary aim assessing safety)</u>. Serious adverse events (SAE) that will be monitored closely in this trial include: 1) Severe neurological deterioration; 2) hospital acquired pneumonia; and, 3) death. Analyses will be descriptive and include rates for SAEs by study cohort.

Severe neurological deterioration (SND) is defined as at least a 4-point increase (≥4) in the NIHSS from the time 0 NIHSS baseline. Our NIHSS serial assessments will allow for early detection of neurologic change in our subjects should it occur. SND is a severe form of deterioration, and consequently, our provision for early trial stopping is a safety measure. Because SND can be due to a number of different factors that may or may not be associated with this protocol, incidents of SND will also be reviewed by the Data Safety Monitoring Board (DSMB)[40] as described below, and adjudicated to the intervention or other factors as indicated by their findings.

Hospital acquired pneumonia (HAP). The American Thoracic Society/Infectious Diseases Society of America (ATS/IDSA) guidelines[41] will be used, defining pneumonia as requiring onset of a new or progressive infiltrate on pulmonary imaging within 72 hours, along with at least two of the following: 1) Fever ≥38° C; 2) purulent sputum; 3) leukocytosis or leukopenia; and/or, 4) decline in oxygenation. Based on our pilots, we are excluding cases from enrollment that are high risk for pneumonia; because these cases are kept NPO for thrombectomy, this will further reduce aspiration risk. We therefore believe that pneumonias will be rare and because of this, we believe that exposure to unnecessary additional chest imaging would add both increased cost and unnecessary radiation exposure, especially since stroke patients often require repeat neuroimaging. Instead, excellent nursing assessments will serve as triggers to inform the need for additional chest imaging in the case of pulmonary deterioration. Because pneumonia may be due to a number of different unassociated factors, pneumonia events will be DSMB reviewed and adjudicated to the intervention as indicated.

Death. Death occurring during the active protocol phase, throughout hospitalization, or within 3 months from enrollment will be monitored, and incidents adjudicated by the DSMB. Death may be associated with a number of unassociated factors, therefore the DSMB will carefully determine study association.

Other AEs detected will be DSMB reviewed and reported as related or unrelated to study procedures.

All the safety outcome analyses will be executed in the intention-to-treat population. We will use unadjusted Cox proportional-hazards modeling to compare and Kaplan-Meier curves to visualize the rate of severe early neurological deterioration (> 4 NIHSS points change from baseline) in the two groups. The number at risk and censored in each group will be reported in the Kaplan-Meier survival curves every 10 minutes. Overall and enrollment group rates of SND will be reported as frequencies and percentages and compared between groups with Fisher's Exact test. The number needed to harm for SND will be estimated as the inverse of the absolute risk increase (ARI) = 1/ARI. Rates of hospital-acquired pneumonia, discharge, 90-day participant's death, post-discharge stroke, and symptomatic intracerebral hemorrhage post-reperfusion treatment will be reported in frequencies and percentages; these will be compared between groups using Fisher's Exact test together with odds ratios and the corresponding 95% confidence intervals and p-values from unadjusted logistic regression models that include randomization assignment as the predictor.

Pre-Specified Exploratory Endpoints: As discussed, END is the primary endpoint for this clinical trial and is measured proximally, at the termination of the intervention monitoring period. Our rationale for not utilizing a post-thrombectomy 90-day outcome is twofold: First, head positioning should not be deemed a "treatment" for LVO stroke; instead, head positioning is a rescue procedure similar to other medical care that is utilized to stabilize, optimize, and ensure both patient safety and optimal outcomes. The impact of a rescue procedure such as head positioning is best measured during the time it is implemented to determine its efficacy in supporting and optimizing patient condition. Second, thrombectomy is a highly effective definitive treatment for large vessel occlusion stroke patients. The tremendous effect size of thrombectomy may likely override any benefit of other interventions, including

head positioning, however this remains unknown at this time. In summary, we aim to determine the efficacy of head positioning as a simple rescue procedure, making measurement of END at the time of positioning on the catheterization lab table for thrombectomy (or immediately prior to elective induction/intubation in sites using general anesthesia for thrombectomy) our choice as the study primary endpoint.

That said, we have identified several pre-specified exploratory endpoints that can be used to examine whether any continued benefit from head positioning occurs in combination with thrombectomy treatment. Specifically, the ZODIAC study will examine the following exploratory endpoints for between group differences:

- 24-hour NIHSS scores
- 7 day or discharge (whichever occurs first) NIHSS scores
- 7 day or discharge (whichever occurs first) mRS
- 90-day mRS

We will examine improvement rates at 24 hours and again at discharge/7 days (whichever comes first) in the NIHSS and will report this as frequencies and percentages; these will be compared between groups using Fisher's Exact test together with odds ratios and the corresponding 95% confidence intervals and p-values from unadjusted logistic regression models that include randomization assignment as the predictor. Changes from baseline NIHSS for individual subjects will be plotted by means of a linear plot according to their randomization group to visualize the changes in NIHSS after randomization in each group; a repeated measures ANOVA will be employed to compare the serial NIHSS measures between the two groups. The 90-day mRS will be analyzed in 3 ways: 1) Dichotomized as 0-2/3-6 and 0-3/4-6 and modeled as a dependent variable by unadjusted logistic regression with randomization assignment as the predictor. The 90-day mRS will be plotted using a paired horizontal bar graph (aka "Grotta bars") for visualization; 2) Analyzed as separate categories and modeled by unadjusted ordinal logistic regression as a dependent variable and with randomization assignment as the predictor. The proportional odds assumption will be verified by likelihood ratio testing; and 3) Transformed to a utility-weighted mRS (UW-mRS)[42] where each of the mRS categories (from 0 to 6) is assigned a weight (1.00, 0.91, 0.76, 0.65, 0.33, 0.00 and 0.00) and modeled as a dependent variable by unadjusted linear regression, with randomization assignment as the predictor.

Of note, ZODIAC is not powered for these exploratory analyses, so interpretation of any findings from these analyses much take this into account.

Data Safety Monitoring Plan

Monitoring Entity: Data Safety Monitoring Board (DSMB). A DSMB has been established to support safety monitoring for this study and will consist of 5 voting members (including the Chair) who are researchers with expertise in clinical trials, imaging, hemodynamics, neurovascular disease and ethics in relation to human subjects research that are external to and independent from any enrolling study team. The PI will work closely with the DSMB to facilitate their expressed needs for making important decisions about any progressively accumulated ethical and efficacy evidence.

Roles and Responsibilities of the DSMB. Dr. Tsivgoulis will serve as Chairman of the DSMB and will work directly with ZODIAC's independent biostatisticians to facilitate data access and obtain any information that may be required from collected data. Dr. Tsivgoulis has substantial expertise in biostatistics as well as vascular neurology, and has led numerous international studies overseeing data management and analyses, and therefore is well suited to lead our DSMB in its examination of intervention efficacy, futility, and safety. He will assume responsibility for the development and submission of all safety reporting to the PI, Institutional Review Boards (IRB), and the NIH, to ensure transparency of our work. Specifically, Dr. Tsivgoulis will develop each agenda for DSMB meetings, conduct meetings using Robert's Rules of Order, chair open, closed and executive sessions, and ensure that meeting summaries and final minutes are adequately prepared and approved as appropriate. Dr. Tsivgoulis will act as the primary contact person for the DSMB. He will be responsible for setting meeting dates and contacting new and, if indicated, ad hoc members to assess their content expertise and orient them to the DSMB process.

The other members of our DSMB will serve as expert objective reviewers of all findings presented to them, and work with Dr. Tsivgoulis to determine the need for additional data or protocol changes as indicated. Full DSMB membership is listed in Table 3. Ms. Erwin Davison, MBA will serve as an ex-officio member of the DSMB in the role of Executive Secretary; her role includes ensuring ongoing compliance with all DSMB members' Human Subjects training, Conflict of Interest (COI) trainings, and COI form completion, ensuring COI forms are approved, working with Dr. Tsivgoulis to establish meeting dates, and working with Dr. Tsivgoulis to procure and assemble required information for each meeting. Ms. Davison will ensure distribution of meeting materials in advance of each meeting to ensure there is adequate time to review materials. She will take notes during open, closed, and executive session of each meeting or during each conference call so that final draft minutes and written meeting summaries can be prepared. Ms. Davison will forward all meeting summaries for DSMB approval within 5 days of each meeting, and she will maintain all protocol documents, COI forms, data, final minutes and meeting summaries from each DSMB meeting in a locked file cabinet within her locked office.

Table 3: DSMB Membership and Roles

DSMB Member	Role	Expertise
Georgios Tsivgoulis, MD, PhD<br>Vascular Neurologist<br>National & Kapodistrian<br>University of Athens	DSMB Chairman<br>Voting Member	Vascular Neurology<br>Clinical Trials<br>Biostatistics<br>DSMB Expert
Erwin Davison, MBA<br>UTHSC Grants Manager	Executive Secretary<br>Ex-officio Member<br>Non-Voting Member	Clinical Trials<br>Research Ethics<br>Grant Reporting
James Rhudy, PhD, DNP, RN<br>Neurology Research Professor	DSMB Member<br>Voting Member	Human Subjects<br>Research Ethics<br>Clinical Trials
Aristeidis Katsanos, MD<br>Vascular Neurologist<br>McMaster University	DSMB Member<br>Voting Member	General Neurology<br>Vascular Neurology<br>Biostatistics<br>Clinical Trials
Joshua Lennon, MD<br>Private Practice Neurologist<br>Regional One Medical Center	DSMB Member<br>Voting Member	Vascular Neurology<br>Sleep Medicine<br>Clinical Trials
Vijay Sharma, MD<br>Vascular Neurologist<br>National University Hospital of Singapore	DSMB Member<br>Voting Member	DSMB Expert<br>General Neurology<br>Vascular Neurology<br>Clinical Trials

All members of ZODIAC's DSMB are independent of this study and not affiliated with any clinical institution that is enrolling in this clinical trial. Additionally, none of the above DSMB members participated in protocol development for this study, nor do they supervise persons who are involved in this study. All DSMB members have completed a COI form in relation to this proposed clinical trial which documents no conflicts of interest with this study. Overall responsibilities of the DSMB members include, 1) the protection of study participants from exposure to unreasonable or unnecessary research risks by monitoring trial data for effectiveness and safety, 2) review of interim data in the context of the most recent scientific literature with the authority to unmask data as deemed necessary, 3) ensuring clinical studies do not continue beyond the point when the objectives have been met and a clinically meaningful

answer of importance to the scientific community and the public has been obtained, and 4) the monitoring of study progress and conduct. Specifically, responsibilities and functions of the DSMB will include:

- Approval of the study protocol, review plans for data and safety monitoring, the informed consent, reporting templates for data presentations, and other items deemed important to review and approve prior to study commencement.
- Establishing guidelines for safety monitoring, including a list of events that should be reported immediately and the format for cumulative reporting at specific intervals.
- Review interim analyses of outcome data, including allowance of unmasking of blinded data for efficacy and futility assessment at pre-specified intervals.
- Review of "toxicity" data to include serious adverse events (SAE), and adverse events (AE), and making recommendations as indicated on whether the trial should continue as originally designed, be revised, suspended or terminated based on observed beneficial or adverse effects related to study interventions.
- Assess trial performance information including recruitment, retention, resource center performance, follow-up information, and listings of protocol violations.
- Review published reports of related studies submitted by the study investigators or DSMB members to determine whether the study needs to be revised or terminated.
- Review proposed modifications to the study prior to their implementation and make recommendations to the PI and/or IRB.
- Review proposed stopping guidelines as specified in the protocol and, at its discretion, recommend modification to the proposed plan or propose a plan if none has been proposed.
- Provide advice and feedback on data analysis to the study statistician or study monitor.
- Monitoring differences in site performance that may warrant site remediation. The DSMB will examine data by enrolling site to determine important trends in events tied to protocol completion and/or patient outcome; findings will be presented in formal reports when issues arise with any enrolling site that requires remediation. As soon as possible, but within 20 days following each DSMB meeting, a written summary will be provided along with justifications related to any recommendations for continuing, changing or terminating the trial. This will be provided along with a statement concerning the impact on the trial of individually observed or cumulative SAEs and AEs as indicated.
- Ensuring the confidentiality of all participant study data used to determine protocol safety.

Role of the PI and Research Coordinator in Safety Monitoring. The PI will hold the ultimate responsibility to report issues related to protocol safety events, as well as concerns about research protocol and data integrity to the DSMB, local and UTHSC IRBs, and NIH. Specifically, the PI will provide written reports to the DSMB on the current status of the trial, interim analyses, adverse events, and problems encountered. The PI's report may contain recommendations for consideration by the DSMB concerning clinical site performance, whether to continue accrual and/or follow up, whether to close the trial, and whether the results should be reported. The PI will also take responsibility for amending the protocol in accordance with DSMB recommendations and notifying the clinical site and IRBs as expeditiously as possible. The PI will provide the DSMB with any modifications to the study prior to their implementation, and she will forward DSMB recommendations and meeting minutes as appropriate to the IRB or other clinical research sites involved. The PI will also provide timely de-identified reports to NIH of unanticipated problems or unexpected SAEs, IRB-approved revisions to the study protocol that indicate a change in risk for participants, summarized recommendations made by the DSMB as appropriate along with an action plan for response, and she will also provide notice of any actions taken

by the IRB regarding the research and responses to these actions. The ZODIAC Research Coordinator will function as a site monitor, auditing 100% of enrolled patients' case report forms (CRFs) alongside source documents and examining informed consent procedures.

DSMB Meetings. DSMB meeting frequency will depend on scheduled meetings and also special called meetings that occur because of an SAE, AE, or other safety related event. The DSMB will meet at least once annually should no special called meetings be necessary. Additionally, meetings and/or conference calls may be held at the request of DSMB members, the study leadership, the NIH Program Director or designee, or the local IRB or UTHSC. All meeting materials provided to the DSMB are considered privileged and confidential and will be watermarked as such by the Executive Secretary. Confidentiality of DSMB materials will be maintained at all times to the extent permitted by law.

Meetings of the DSMB will be divided as follows:

1) Open Session – which may include members of the clinical trial team, NIH staff, and ad hoc members at the direction of the DSMB Chair for the purpose of general trial conduct and progress discussions including "toxicity" issues (SAEs, AEs), subject accrual, protocol compliance, site performance, quality control, follow-ups, or other general items. No confidential data will be shared during open sessions of the DSMB, and blinding will be maintained during open sessions.

2) Closed Session – during this session, attendance will be limited to voting members, any ex-officio members invited by the Chair, and the Executive Secretary; NIH staff may be invited to attend this portion of the meeting but will act strictly as observers and will not participate in deliberations or provide additional information that may influence recommendations. Information discussed during closed sessions includes aggregated safety data, efficacy, and futility data – including unmasking of blinded data presented by the statistician if necessary to assure participant safety. NIH staff are not privy to post-randomization data broken down by treatment groups that may be discussed.

3) Executive Session – Only voting members and the Executive Secretary may be present in an executive session. Members will discuss the general conduct of the trial, outcome results including SAEs and AEs and implications. Should the Chair ask the Executive Secretary to excuse herself, he will then assume responsibility for recording minutes and recommendations of this session. The Chair may break the blind if this is deemed necessary to make decisions about efficacy or futility. Recommendations coming out of executive session will include A) study continuation, B) study termination, C) study suspension, and, D) the need for study protocol revisions. At the Chair's discretion, NIH staff may be invited to attend, but may only be observers in the process and are not privy to unmasking of blinded data. The DSMB will vote on recommendations in executive session and efforts will be made to obtain a consensus. If consensus cannot be obtained, a majority vote will carry a recommendation. The Chair will participate in discussions and will vote. Should a minority opinion be present, this will be reported alongside the recommendation. The Executive Secretary (or Chair if the Executive Secretary is excused) will document the discussion and recommendations. The final recommendations must be summarized either as majority or minority positions or as actual vote tallies for the various divergent recommendations.

Written Meeting Summaries of DSMB Recommendations. A written meeting summary that identifies topics discussed by the DSMB describing individual findings, overall safety assessment and recommendations with justification related to continuing, changing, suspending, or terminating the trial and the impact on the trial of individually observed or cumulative adverse events will be signed by the Chair. This report should contain no mention of safety, efficacy or futility data by treatment group. Written meeting summaries must be submitted to NIH and the local IRB within 14 days of meeting occurrence.

In the absence of disagreement, the PI must act to implement the recommendations as expeditiously as possible by amending the protocol. Should the PI disagree with the DSMB, she will be responsible for reaching a mutually acceptable decision about the study with the IRB. Meeting minutes will be prepared, and a final draft version will be signed/approved by the DSMB Chair. Minutes should include: 1) General highlights of the discussion; 2) general recommendations; 3) actionable items; 4) suggested protocol/study changes and rationale for each; and, 5) the date for the next scheduled meeting of the DSMB. Minutes will not contain confidential data.

Release of Outcome Data. Confidential outcome data should not be made available to individuals outside of the DSMB. Any release of outcome data to individuals outside the DSMB must be reviewed and approved by the DSMB, the local IRB, and the PI.

Confidentiality Procedures. No communication, either written or oral, of the deliberations or recommendations of the DSMB will be made outside of the DSMB except as provided for in these guidelines. Outcome results are strictly confidential and must not be divulged to any non-member of the DSMB.

Procedures for Monitoring, Minimizing Risk, and Protecting Confidentiality of Participant Data

Procedures for Monitoring Study Safety. The DSMB will follow a standard monitoring schedule and also hold special called monitoring meetings based on the occurrence of an SAE or other safety, IRB compliance, or intervention performance concerns that may arise. The standard monitoring schedule for the DSMB is determined by enrollment milestones. A total of 4 “looks” at enrollment data will be scheduled with “look 1” occurring after the first 46 patients are enrolled, “look 2” after 92 patients, “look 3” after 138 patients, and “look 4” – the final look – after all 182 patients are enrolled (Table 2).

The PI will hold responsibility for notifying the DSMB Chair and IRB Chair of the occurrence of any SAE (as previously defined) and this will trigger a special called meeting of the DSMB. PI notification to the DSMB Chair, IRB and NIH will occur within 24 hours of SAE occurrence. The DSMB Chair will hold authority for assembling data for review by the full DSMB on all SAE cases. Each SAE case will be adjudicated as either “associated with the intervention” or “unassociated with the intervention” by the DSMB, and this determination will be reported by the DSMB Chair formally in writing to the PI, the IRB, and NIH. The FDA standards for “serious,” “anticipated” and “associated with the treatment” will be employed. The DSMB will follow SAE occurrences adjudicated to the intervention for trends suggestive of harm that may cause the study to be suspended, revised or terminated, and will independently hold the authority to stop the trial based on such a determination.

The DSMB will audit select cases to ensure compliance with IRB requirements. The ZODIAC research coordinator will work as the clinical monitor and will be authorized to audit all CRFs and source documents. All CRFs will be visible to the research coordinator online through the web-based enrollment system, with auto-notification sent from the system for each enrollment. Site visits will be triggered by enrollment numbers, so that for every 3 cases enrolled, the research coordinator will visit the site to further examine:

- Investigators’ compliance with protocol and IRB requirements;
- Sites conformance with informed consent requirements; and,
- Verification of source documents supporting the CRF data documented.

Sites determined to be out of compliance will be reported by the research coordinator to the PI. In turn, the PI will notify the local IRB and UTHSC IRB, as well as the Chair of the DSMB. Depending on the

infraction identified, the PI, the DSMB Chair and the UTHSC IRB Chair will determine the appropriate course of action which may include site termination.

Procedures for Minimizing Research-Associated Risk. The following procedures will be employed within the study protocol and by the DSMB to ensure study safety:

- Study protocol exclusions to ensure safety. Preliminary work supporting this study has examined protocol safety for head positioning interventions in detail to determine patients that should be included, as well as those that should be excluded because of safety risks. Our inclusion and exclusion criteria reflect this information. Specifically, our exclusions include patients who may be at undue risk due to laying flat, as well as those with concurrent diagnoses or clinical findings that may confound our ability to understand protocol safety.

- DSMB procedures to ensure safety. Procedures to ensure safety utilized by the DSMB will include approval of the study protocol and the informed consent, establishing a guideline for safety monitoring that includes a list of events that should be reported immediately, revieweing data from planned interim analyses, reviewing toxicity data that includes SAEs and AEs to determine if these events are intervention and trial related, and deciding on the need for study method revisions, study suspension and study termination based on findings. As mentioned previously, the PI will alert the DSMB of safety incidents warranting further evaluation. The research coordinator will monitor protocol compliance and notify the PI of sites with compliance concerns which the PI will in turn report to the DSMB Chair, the IRB and NIH. The DSMB Chair will convene special called meetings to address safety incidents and protocol compliance. The DSMB will hold planned meetings to review findings from efficacy and futility analyses when enrollment milestones have been met. The DSMB will also assess overall trial performance information including recruitment, retention, resource center performance, follow-up information, and listings of protocol violations. The DSMB will also be charged with reviewing proposed modifications to the study prior to their implementation and make recommendations about proposed changes to the PI and/or IRB as indicated. The DSMB and study investigators will also stay abreast of new findings in practice that may call for a different approach. Collectively, these methods will minimize any undue risk in our enrolled patients. Safety decisions involving the need to suspend or terminate the study will be communicated immediately (within no more than 24 hours) by the DSMB to the PI, the IRB, and NIH.

Procedures for Protecting the Confidentiality of Patient Data. The work of the DSMB will be supported by strict confidentiality. All DSMB members will complete COI forms and be vetted for inclusion on the monitoring board. Confidential outcome data reviewed by the trial will not be made available to individuals outside of the DSMB, and any release of outcome data to individuals outside the DSMB must be reviewed and approved by the DSMB, the local IRB, and the PI. Additionally, no communication, either written or oral form, of the deliberations or recommendations of the DSMB will be made outside of the DSMB except as provided for in these guidelines. Outcome results are strictly confidential and will not be divulged to any non-member of the DSMB without the expressed approval of the PI, the IRB and NIH.

In accordance with UTHSC IRB and NIH requirements, the ZODIAC protocol will utilize methods that ensure the confidentiality of our data. The UTHSC Center for Biomedical Informatics (CBMI) has

provided the web-based customized biomedical clinical-research informatics application for enrollment, randomization, and data entry. Our web-based data system is a fully customizable integrated service for data collection and project alerts linked to an encrypted PostgreSQL database, and is accessed via the Internet though secure web-applications. The UTHSC network and computing facilities are owned and managed by UTHSC-ITS; all CBMI systems are housed in the ITS computer center, which has electric power conditioning, UPS battery backup for short-term outages, and a backup diesel generator for long-term outages. The computer center also has fire suppression, temperature and humidity control, card key controlled access and video monitoring. Network traffic crossing the UTHSC network boundary is examined by a Cisco firewall against its Access Control Lists. Only the research coordinator and ZODIAC's independent biostatisticians are able to review data within the system. Authority for use will be strictly monitored to ensure that system use is appropriate.

<u>Procedures for Identifying, Reviewing, and Reporting Adverse Events and Unanticipated Problems to IRB and NIH</u>

Safety is a secondary aim of this study and will be examined in this larger, more generalizable sample of patients. Specifically, SAEs that will be monitored closely in this trial include: 1) Severe neurological deterioration (SND) defined as a deterioration of $\geq$ 4 points on the NIHSS from the time-0 NIHSS; 2) hospital acquired pneumonia (HAP) as defined by the American Thoracic Society/Infectious Disease Society of America;[41] and, 3) death. As SAEs occur, the DSMB, IRB and NIH will be notified by the PI. The DSMB Chair will convene a special called meeting (as described previously) to determine whether the SAE should be adjudicated to the intervention or other unrelated cause. The DSMB will follow the methods for meeting conduct as described previously in this document, along with the written report methods specified. Beyond individual SAE review, the DSMB will examine analyses as directed by the statistical plan. The DSMB will formally report their findings to the PI, IRB and NIH.

<u>Procedures to Ensure Monitoring Plan Compliance and Reporting Requirements for Study Sites</u>

All regular meetings of the DSMB will include discussion of individual and aggregate site performance, including adherence to all aspects of the study protocol, completeness of CRFs, accuracy of source data in relation to CRF completeness, compliance of informed consent procedures, and trends noted in any of these areas. As described previously, the research coordinator will monitor 100% of CRFs submitted on patients. The web-based CRF system automatically flags required fields so that deficiencies are easily detected. After every 3 cases, the research coordinator will travel to a site to examine source documents in relation to CRFs. All deficiencies noted by the research coordinator will be reported to the PI, and the PI in turn will notify the DSMB when protocol compliance concerns arise. The DSMB will evaluate individual site compliance but also will aggregate site compliance issues as needed, so that they may identify if multiple sites are non-compliant with certain aspects of the protocol, as such findings may indicate an unrealistic approach to study methods that may require protocol revision. The DSMB will notify the PI, IRB and NIH of compliance findings in individual sites and by aggregate, and will recommend actionable measures to improve compliance that may include site remediation, or site termination as indicated by the infraction.

<u>Procedures for Assessment of External Factors or Relevant Information that may Influence Study Participant Safety</u>

All investigators and members of the DSMB hold responsibility for scanning the practice environment, keeping current with standards of care, possessing knowledge of evolving care standards, and reviewing related research findings that may impact the safe enrollment of subjects in this study. In particular, factors that may impact the receipt of timely standard of care reperfusion treatments will be evaluated in

an ongoing manner to ensure that study participants are not disadvantaged by participating in this study protocol.

Plans for Interim Efficacy and Futility Analyses

ZODIAC is a efficacy study (Aim 1) that will determine superiority of one of two head positions in patients with large vessel occlusion hyperacute ischemic stroke prior to commencement of thrombectomy. Because the primary endpoint of clinical stability in and of itself constitutes a safety endpoint, we have developed a plan to examine both protocol efficacy and protocol futility with defined stopping boundaries (Table 2 repeated below). Using sequential testing, the DSMB will take a first look at data when 46 participants are enrolled and have had their primary outcome evaluated. For example, the DSMB will stop the trial for efficacy (with one group clearly superior) if the z-value associated with the test of equality of proportions is above 4.333 or below -4.333, whereas if that z-value is in the interval (-0.007, 0.007), the DSMB will stop the trial early for futility. However, if the z-value falls within the intervals (-4.333, -0.007) or (0.007, 4.333), the DSMB will continue the trial until the next look at the data when 92 participants are available. As described previously under the meetings section of this plan, the DSMB will hold regularly scheduled meetings based on achievement of these Table 2 enrollment milestones, and continue to assess efficacy and futility at each of these meetings until the point of trial completion is determined. The DSMB will formally report these findings to the PI, IRB, and NIH using the written report methods described above.

Table 2: Stopping Boundaries for ZODIAC's Sequential Design.

Look #	Sample Size<br>MT Subjects	Stopping Boundaries<br>Efficacy Z (lower, upper)		Futility Z (lower, upper)	
1	46	4.333	-4.333	0.007	-0.007
2	92	2.963	-2.963	0.374	-0.374
3	138	2.359	-2.359	1.261	-1.261
4	182	2.014	-2.014	2.014	-2.014

Lastly, the PI will collaborate with the DSMB, the IRB and NIH to modify this DSMP to address additional concerns or issues that may arise. Therefore, this plan may be subject to change as the protocol is implemented; any changes to this plan will be immediately submitted to NIH and our IRBs.

REFERENCES

1. Wojner AW, El-Mitwalli A, Alexandrov AV. Effect of head positioning on intracranial blood flow velocities in acute ischemic stroke: A pilot study. *Critical Care Nursing Quarterly*. 2002;24(4):57-66.
2. Wojner-Alexandrov AW, Garami Z, Chernyshev OY, Alexandrov AV. Heads down: Flat positioning improves blood flow velocity in acute ischemic stroke. *Neurology*. 2005;64(8):1354-1357.
3. Hunter AJ, Snodgrass SJ, Quain D, Parsons MW, Levi CR. HOBOE (Head-of-Bed Optimization of Elevation) Study. *Physical Therapy*. 2011;91(10):1503-1512.
4. Ali LK, Weng JK, Starkman S, Saver JL, Kim D, Ovbiagele B, Buck BH, Sanossian N, Vespa P, Bang OY, Jahan R, Duckwiler GR, Vinuela F, Liebeskind, DS. Heads Up! A novel provocative maneuver to guide acute ischemic stroke management. *Interventional Neurology*. 2017;6(1-2):8-15.
5. Durduran T, Zhou C, Yu G, Edlow B, Choe R, Shah Q, Kasner SE, Cucchiara BL, Yodh AG, Greenberg JH, Detra JA. Bedside monitoring of cerebral blood flow in acute stroke patients during changes in head of bed position. *Stroke*. 2007; 38(2):494.
6. Toole, JF. Effects of change of head, limb and body position on cephalic circulation. *New England Journal of Medicine*. 1968;279(6):307-311.
7. Caplan LR, Sergay S. Positional cerebral ischaemia. *Journal of Neurology, Neurosurgery and Psychiatry*. 1976;39(4):385-391.
8. Hayashida K, Hirose Y, Kaminaga T, Ishida Y, Imakita S, Takamiya M, Yokota I, Nishimura T. Detection of postural cerebral hypoperfusion with technetium-99m-HMPAO brain SPECT in patients with cerebrovascular disease. *Journal of Nuclear Medicine*. 1993;34(11):1931-5.
9. Ouchi Y, Nobezawa S, Yoshikawa E, Futatsubashi M, Kanno T, Okada H, Torizuka T, Nakayama T, Tanaka K. Postural effects on brain hemodynamics in unilateral cerebral artery occlusive disease: A positron emission tomography study. *Journal of Cerebral Blood Flow and Metabolism*. 2001;21(9):1058-1066.
10. Hargroves D, Tallis R, Pomeroy V, Bhalla A. The influence of positioning upon cerebral oxygenation after acute stroke: A pilot study. *Age and Ageing*. 2008;37(5):581-585.
11. Anderson CS, Arima H, Lavados P, Billot L, Hackett, ML, Olavarria VV, Munoz Venturelli P, Brunser A, Peng B, Cui L, Song L, Rogers, K, Middleton S, Lim JY, Forshaw D, Lightbody CE, Woodward M, Pontes-Neto, O, De Silva, HA, Lin R-T, Lee, TH, Pandian JD, Mead GE, Robinson T, Watkins C, for the HeadPoST Investigators and Coordinators. Cluster-randomized, crossover trial of head positioning in acute stroke. *NEJM*. 2017;376:2437-47.
12. Alexandrov AW, Tsivgoulis G, Hill MD, Liebeskind DS, Schellinger P, Ovbiagele B, Arthur A, Caso V, Nogueira R, Hemphill JC, Grotta JC, Hacke W, Alexandrov AV. HeadPoST: Rightly positioned, or flat out wrong? *Neurology.* 2018;90(18):885-889.
13. American Heart Association News. Head position after stroke: Up or down? *American Heart Association*, International Stroke Conference News Stories. Blog, International Stroke Conference 2017, February 22, 2017.
14. Fiore K. Lying flat, sitting up equal for mild stroke recovery. *Medpage Today*. www.medpagetoday.com/MeetingCoverage/ISC/63345?xid=nl_mpt_cardiodaily_2017-02-22&eun=g1084220d0r. Accessed September 15, 2017.
15. Diener H-C. Flawed studies define this year's International Stroke Conference. *Medscape Neurology*. March 7, 2017.
16. Grotta JC, Welch KM, Fagan SC, Lu M, Frankel MR, Brott T, Levine SR, Lyden PD. Clinical deterioration following improvement in the NINDS rt-PA Stroke Trial. *Stroke*. 2001;32(3):661-668.
17. Davalos A, Toni D, Iweins F, Lesaffre E, Bastianello S, Castillo J. Neurological deterioration in acute ischemic stroke: Potential predictors and associated factors in the European Cooperative Acute Stroke Study (ECASS) I. *Stroke*. 1999;30(12):2631-2636.

18. Jauch EC, Saver JL, Adams HP Jr, Bruno A, Connors JJ, Demaerschalk BM, Khatri P, McMullan PW Jr, Qureshi AI, Rosenfield K, Scott PA, Summers DR, Wang DZ, Wintermark M, Yonas H; American Heart Association Stroke Council; Council on Cardiovascular Nursing; Council on Peripheral Vascular Disease; Council on Clinical Cardiology. Guidelines for the early management of patients with acute ischemic stroke: a guideline for healthcare professionals from the American Heart Association/American Stroke Association. *Stroke*. 2013 Mar;44(3):870-947
19. Powers WJ, Derdeyn CP, Biller J, et al. 2015 AHA/ ASA Focused Update of the 2013 Guidelines for the Early Management of Patients With Acute Ischemic Stroke Regarding Endovascular Treatment: a Guideline for Healthcare Professionals From the American Heart Association/American Stroke Association. *Stroke*. 2015;46(10):3020-3035.
20. Summers D, Leonard A, Wentworth D, Saver JL, Simpson J, Spilker JA, Hock N, Miller E, Mitchell PH. American Heart Association Council on Cardiovascular Nursing and Stroke Council. Comprehensive overview of nursing and interdisciplinary care of the acute ischemic stroke patient: A scientific statement from the American Heart Association. *Stroke*. 2009;40(8):2911-2944.
21. *The Joint Commission*. www.jointcommission.org/certification/certification_main.aspx. Accessed September 15, 2017.
22. Berkhemer OA, Fransen PS, Beumer D, van den Berg LA, Lingsma HF, Yoo AJ, Schonewille WJ, Vos JA, Nederkoorn PJ, Wermer MJ, van Walderveen MA, Staals J, Hofmeijer J, van Oostayen JA, Lycklama à Nijeholt GJ, Boiten J, Brouwer PA, Emmer BJ, de Bruijn SF, van Dijk LC, Kappelle LJ, Lo RH, van Dijk EJ, de Vries J, de Kort PL, van Rooij WJ, van den Berg JS, van Hasselt BA, Aerden LA, Dallinga RJ, Visser MC, Bot JC, Vroomen PC, Eshghi O, Schreuder TH, Heijboer RJ, Keizer K, Tielbeek AV, den Hertog HM, Gerrits DG, van den Berg-Vos RM, Karas GB, Steyerberg EW, Flach HZ, Marquering HA, Sprengers ME, Jenniskens SF, Beenen LF, van den Berg R, Koudstaal PJ, van Zwam WH, Roos YB, van der Lugt A, van Oostenbrugge RJ, Majoie CB, Dippel DW; MR CLEAN Investigators. A randomized trial of intraarterial treatment for acute ischemic stroke. *New England Journal of Medicine*. 2015;372:11–20.
23. Goyal M, Demchuk AM, Menon BK, Eesa M, Rempel JL, Thornton J, Roy D, Jovin TG, Willinsky RA, Sapkota BL, Dowlatshahi D, Frei DF, Kamal NR, Montanera WJ, Poppe AY, Ryckborst KJ, Silver FL, Shuaib A, Tampieri D, Williams D, Bang OY, Baxter BW, Burns PA, Choe H, Heo JH, Holmstedt CA, Jankowitz B, Kelly M, Linares G, Mandzia JL, Shankar J, Sohn SI, Swartz RH, Barber PA, Coutts SB, Smith EE, Morrish WF, Weill A, Subramaniam S, Mitha AP, Wong JH, Lowerison MW, Sajobi TT, Hill MD; ESCAPE Trial Investigators. Randomized assessment of rapid endovascular treatment of ischemic stroke. *New England Journal of Medicine*. 2015;372:1019–30.
24. Campbell BC, Mitchell PJ, Kleinig TJ, Dewey HM, Churiloy L, Yassi N, Yan B, Dowling RJ, Parsons MW, Oxley TJ, Wu TY, Brooks M, Simpson MA, Miteff F, Levi CR, Krause M, Harrington TJ, Faulder KC, Steinfort BS, Priglinger M, Ang T, Scroop R, Baraber PA, McGuinness B, Wijeratne T, Phan TG, Chong W, Chandra RV, Bladin CF, Badve M, Rice H, de Villiers L, Ma H, Desmond PM, Donnon GA, Davis SM; EXTEND-IA Investigators. Endovascular therapy for ischemic stroke with perfusion-imaging selection. *New England Journal of Medicine*. 2015;372:1009–18.
25. Saver JL, Goyal M, Bonafe A, Diener HC, Levy EI, Pereira VM, Albers GW, Cognard C, Cohen DJ, Hacke W, Jansen O, Jovin TG, Mattle HP, Nogueira RG, Siddiqui AH, Yavagal DR, Baxter BW, Devlin TG, Lopes DK, Reddy VK, du Mesnil de Rochemont R, Singer OC, Jahan R; SWIFT PRIME Investigators. Stent-retriever thrombectomy after intravenous t-PA vs. t-PA alone in stroke. *New England Journal of Medicine*. 2015;372:2285–95.
26. Jovin TG, Chamorro A, Cobo E, de Miquel MA, Molina CA, Rovira A, San Román L, Serena J, Abilleira S, Ribó M, Millán M, Urra X, Cardona P, López-Cancio E, Tomasello A, Castaño C, Blasco J, Aja L, Dorado L, Quesada H, Rubiera M, Hernandez-Pérez M, Goyal M, Demchuk AM,

von Kummer R, Gallofré M, Dávalos A; REVASCAT Trial Investigators. Thrombectomy within 8 hours after symptom onset in ischemic stroke. *New England Journal of Medicine*. 2015; 372:2296–306.

27. Derdeyn CP, Chimowitz MI, Lynn MJ, Fiorella D, Turan TN, Janis LS, Montgomery J, Nizam A, Lane BF, Lutsep HL, Barnwell SL, Waters MF, Hoh BL, Hourihane JM, Levy EI, Alexandrov, AV, Harrigan MR, Chiu D, Klucznik RP, Clark JM, McDouglall CG, Johnson MD, Pride GL Jr, Lynch JR, Zaidat OO, Rumboldt Z, Cloft HJ; Stenting and Aggressive Medical management for Preventing Recurrent Stroke in Intracranial Stenosis Trial Investigators. Aggressive medical treatment with or without stenting in highrisk patients with intracranial artery stenosis (SAMMPRIS): The final results of a randomised trial. Lancet. 2014;383(9914):333-341.
28. Bartholomew Eldridge LK, Markham CM, Ruiter RAC, Fernandez ME, Kok G, Parcel GS. Chapter 9: Intervention mapping, step 6. In, *Planning Health Promotion Programs: An Intervention Mapping Approach*, 4th ed. 2016: John Willey & Sons. Published by Jossey-Bass.
29. Moore GF, Audrey S, Barker M, Bond L, Bonnell C, Hardeman W, Moore L, O'Cathain A, Tinati T, Wight D, Baird J. Process evaluation of complex interventions: Medical research council guidance. *British Medical Journal*. 2015;350:h1258 1 doi:10.1136/bmjh1258.
30. Campbell M, Fitzpatrick R, Haines A, Kinmonth AL, Sandercock P, Spiegelhalter D, Tyrer P. Framework for design and evaluation of complex interventions to improve health. *British Medical Journal*. 2000;321:694-696.
31. Carroll C, Patterson M, Wood S, Booth A, Rick J, Balain S. A conceptual framework for implementation fidelity. *Implementation Science*. 2007;2:40. Doi:10.1186/1748-5908-2-40.
32. Santacroce SJ, Maccarelli LM, Grey M. Intervention fidelity. *Nursing Research*. 2004:53(1):63-66.
33. Meinert CL, Tonascia S. Clinical Trials: Design, Conduct, and Analysis. New York: Oxford University Press; 1986.
34. Murray DM. *Design and Analysis of Group-Randomized Trials*. New York: Oxford University Press. 1998; p. 352.
35. Donner A, Klar N. Pitfalls of and controversies in cluster randomization trials. Am J Public Health. 2004 Mar;94(3):416-22.
36. Martin DC, Diehr P, Perrin EB, et al. The effect of matching on the power of randomized community intervention studies. *Stat Med.* 1993;12:329–338.
37. Jennison, C., and B. W. Turnbull: Group Sequential Methods with Applications to Clinical Trials. Chapman & Hall/CRC, 2000.
38. Montgomery D. *Introduction to Statistical Quality Control*. Hoboken, New Jersey: John Wiley & Sons, Inc. 2005.
39. Piantadosi S. Clinical Trials - A Methodologic Perspective. Second ed. Hoboken, New Jersey: Wiley; 2005.
40. Herson J. Data and Safety Monitoring Committees in Clinical Trials. Boca Raton, FL: Chapman & Hall/CRC; 2009.
41. American Thoracic Society; Infectious Diseases Society of America. Guidelines for the management of adults with hospital-acquired, ventilator-associated, and healthcare-associated pneumonia. *Am J Respir Crit Care Med*. 2005 Feb 15;171(4):388-416.
42. Chaisinanunkul N, Starkman S, Gornbein J, Hamilton S, Chatfield F, Conwit R, Saver JL. Staged use of ordinal and linear disability scales: a practical approach to granular assessment of acute stroke outcome. Front Neurol. 2023 Jun 28;14:1174686. Erratum in: Front Neurol. 2023 Dec 12;14:1331276.

ZODIAC PROTOCOL (version 2.0)

Protocol Title: Zero Degree Head Positioning in Hyperacute Large Artery Ischemic Stroke (ZODIAC)

Trial registration: ClinicalTrials.gov ID - NCT03728738

Sponsor:

University of Tennessee

1R01NR017850-01 (National Institutes of Health)

Principal Investigator: Anne W. Alexandrov **Date:** September 16, 2019

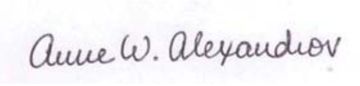

SPECIFIC AIMS

Positioning of the patient with hyperacute large artery ischemic stroke is an important, yet understudied aspect of care that could impact the course of treatment and ultimately clinical outcome for this most severe type of stroke patient. Positioning with the head of bed (HOB) at 0° has been shown in small studies to increase blood flow across points of arterial stenosis in hyperacute large artery ischemic stroke, leading to clinical improvement in stroke symptoms.[1-5] Since 1968, small studies have also documented clinical symptom worsening in ischemic stroke patients when the HOB has been elevated to 30° or higher.[6-10] A recent large cluster randomized trial (HeadPoST) attempted to determine what head position was best in stroke, but failed to enroll the patient cohort for which 0° head positioning has been shown to benefit.[11] Findings from HeadPoST have been highly criticized by the leadership of all international stroke societies due to significant design flaws, enrollment of a subacute small vessel and intracerebral hemorrhage sample for whom the damage of stroke had already occurred, and questionable intervention fidelity.[12-15] In fact, HeadPoST has become a catalyst for a now urgent call to conduct high quality head positioning research in hyperacute large artery ischemic strokes to definitively answer the question of how best to manage these highly vulnerable patients to ensure stability and prevent symptom worsening.[12]

Early neurologic deterioration is common in hyperacute ischemic stroke, ranging from 15%[16] to 37.5%[17] in large clinical trials, and selection of HOB positioning may play an important role in its occurrence. However, to date, clinical trial protocols exploring numerous drug and device interventions have remained silent on HOB positioning. Mechanisms proposed for clinical improvement at 0°-HOB include favorable gravitational arterial flow conditions,[2,3] recruitment of collateral arterial blood flow routes,[2-4] and in the case of intravenous thrombolysis, improved clot-lytic interactions augmenting arterial recanalization.[2] Collectively, these mechanisms have caused many to argue that 0°-HOB positioning should be among the first steps taken in large artery hyperacute ischemic stroke patient management. The goal of this efficacy study is to determine if 0°-HOB positioning in hyperacute ischemic stroke prevents neurological symptom worsening in large artery occlusion patients. The study will achieve these goals through use of a novel protocol enabling enrollment of consecutive large artery occlusion patients while maintaining compliance with management specified by national guidelines,[18-20] stroke center certification requirements,[21] and U.S. core measures.[18-21] We will enroll hyperacute large artery patients with salvageable brain early into stroke symptoms, that are candidates for mechanical thrombectomy (MT) (n=182). Patients will be randomized to one of two groups: 1) 0°-HOB positioning; or, 2) 30° HOB positioning. The primary endpoint will be early neurological deterioration (stroke symptom worsening) defined as an increase of 2 or more points from baseline (pre-intervention) National Institutes of Health Stroke Scale (NIHSS) score during the positioning period. These data will be employed to meet the following specific aims:

Aim 1 (primary efficacy endpoint): To identify if use of 0° HOB positioning is associated with clinical stability in hyperacute ischemic stroke. *Hypothesis*: *Patients with large artery occlusions placed in a 0° HOB position (V_1), (superiority hypothesis), will experience less early neurologic deterioration within the time prior to thrombectomy, than those in the 30° HOB elevation group (V_c)*, or H_0: $V_1 = V_c$ versus H_A: $V_1 < V_c$.

Aim 1 will be supported by a process evaluation plan that identifies key components (context; reach and recruitment; implementation dose delivered and received; and, overall fidelity), related questions, and associated process stability indicators.[22-26] Implementation methods are proposed to support, continuously monitor, report, and improve process stability and overall protocol fidelity.

Aim 2 (secondary aim assessing safety): To confirm the safety of 0°-HOB positioning in a large, generalizable sample of hyperacute large artery ischemic stroke patients.

RESEARCH PROTOCOL

This phase III study will utilize a multicenter, prospective randomized outcome-blinded evaluation (PROBE) approach enrolling consecutive hyperacute large artery ischemic stroke patients to determine if use of 0°-HOB positioning is associated with greater clinical stability. PROBE designs are among the most well-respected design options within the stroke scientific community when blinding of both subjects and enrolling clinicians is impossible and have been used to support all recent large artery ischemic stroke clinical trials.[22-27]

Site Eligibility. Sites eligible for participation in ZODIAC must meet the following criteria:

- Research infrastructure to support a clinical trial
- Certification as a comprehensive stroke center or thrombectomy-capable stroke center
- Rapid response stroke team capable of STAT emergency in-person presence at the time of acute stroke arrival
- Stroke team leadership by fellowship trained vascular neurologists and neurovascular fellowship trained ANVP board certified advanced practice providers
- Rapid response stroke team certification in both the NIHSS and the modified Rankin Score (mRS); team member inter-rater reliability of scores consistently between 97-100% for both NIHSS and mRS
- Compliance with stroke quality core measures consistently documented at ≥ 90% over the previous 6 months

Inclusion and Exclusion Criteria. Adult (≥18 years) hyperacute ischemic stroke patients will be screened for enrollment. Because large artery ischemic stroke is not unique to any one sex, we will enroll both women and men.

Inclusion Criteria-

- Ischemic stroke symptoms consistent with large artery occlusion
- Baseline standard of care non-contrast head CT (or MRI) negative for hemorrhage or mass-effect
- Evidence of arterial occlusion on standard of care CT angiography or MR angiography
- Favorable neuroimaging (Alberta Stroke Program Early Computed Tomography Score [ASPECTS] ≥ 6 in anterior circulation stroke; not applicable in posterior circulation stroke)
- Anticipated treatment with mechanical thrombectomy
- Pre-stroke baseline modified Rankin Score (mRS) ≤ 1
- Favorable imaging with ASPECTS ≥ 6 and/or favorable CT perfusion scan (according to local site standard of care for thrombectomy patient selection) up to 24-hours from time of symptom onset

Exclusion Criteria-

- Pregnancy or suspicion of pregnancy
- Evidence or suspicion of vomiting any time prior to consent which could predispose to aspiration pneumonia and therefore confound determination of protocol safety
- Anticipated palliative care referral
- Evidence of evolving malignant infarction on admission noncontrast CT (or MRI)
- Need for emergent intubation with mechanical ventilation, or non-invasive ventilatory support with either bi-level positive airway pressure (BiPAP) or continuous positive airway pressure (CPAP) due to pending or actual respiratory failure prior to or at the time of emergency department

admission. (Note: Elective intubation for the thrombectomy procedure is not an exclusion criterion.)

- Inability to tolerate zero-degree positioning due to congestive heart failure, preexisting pneumonia, chronic obstructive pulmonary disease, or other medical condition. (Note: A diagnosis of heart failure or chronic obstructive pulmonary disease does not automatically exclude enrollment; each patient should be assessed individually for positional intolerance.)
- Admission chest radiograph positive for pleural effusion, pulmonary edema, pneumonia, or other pulmonary condition that may confound determination of protocol safety. (Note: An admission chest x-ray is not required, but may be obtained in patients with concerning pulmonary findings.)
- Abnormal breath sounds on admission assessment that may confound determination of protocol safety
- Lack of a telephone and/or permanent address predisposing patients to be lost to follow up
- Enrollment in another clinical trial that may affect our primary or secondary endpoints
- In the absence of a consenting legal next of kin, any medical, psychological, cognitive, social or legal condition that would interfere with informed consent and/or capacity to comply with all study requirements, including the necessary time commitment
- Note: Enrollment of patients receiving systemic thrombolysis more than 15 minutes prior to randomization is discouraged as this may confound ability to understand the impact of head positioning on clinical stability.

Blinding. Site principal and co-investigators will hold ZODIAC aims, methods, and enrollment in confidence throughout conduct of the trial from other members of the stroke team working in the emergency department, catheterization lab, and stroke unit, including stroke coordinators, rapid response team members, emergency physicians and nurses, neurointerventionalist physicians, and physician residents. Because emergency stroke care is delivered in a hectic fast-paced environment where each responding team member is consumed with their own specific responsibilities, our pilot work has demonstrated that masking of enrollment procedures is easily accomplished. Additionally, because head positioning is often overlooked and commonly varies between providers and patient conditions, pilots of this protocol have shown that practitioners do not notice that some patients are positioned with the head up, and others are positioned with the head down. Therefore, blinded serial measurement of the NIHSS can be accomplished using protocol naïve certified practitioners for collection of endpoints.

Subject Screening, Enrollment, and Serial Monitoring (Figure)

Standard of Care: Suspected acute stroke patients will undergo guideline-supported standard of care stroke team emergency response procedures, including assessment/stabilization of airway, breathing, circulation, placement of a continuous pulse oximetry sensor, with immediate transport for imaging. The admission NIHSS will be scored on route to the imaging suite while the patient is in whatever position prehospital personnel have selected (this assessment is not utilized in the ZODIAC protocol but is a stroke center certification agency requirement). Continuous portable ECG monitoring with nasal cannula oxygen (if necessary, based on pulse oximetry) will be established in CT (or MRI), and IV access will be obtained with STAT blood draw; point of care testing will be conducted immediately before STAT non-contrast CT (or MRI) with CTA (or MRA). A rapid/detailed history/physical exam will be completed concurrently and patients eligible for treatment with tissue plasminogen activator (tPA) will have drug administered in CT (or MRI).

Enrollment and Serial Monitoring: Subjects meeting inclusions without exclusions will be consented and randomized. All subjects (0-degree and 30-degree) will be maintained at 0-degrees immediately after completion of neuroimaging. An NIHSS-certified practitioner naïve to the research protocol will measure the baseline/time 0 NIHSS score while the patient is at 0-degrees. The local investigator will then position the patient in the randomly assigned position and the serial NIHSS (every 10 minutes) monitoring phase of the intervention will begin. The site investigator must stay with the patient until positioned on the catheterization lab table to ensure assigned head position is maintained. The serial NIHSS monitoring phase ends immediately prior to placement on the catheterization lab table, with the primary endpoint measured during handoff by the same protocol naïve stroke team member to a protocol naïve catheterization lab practitioner certified in the NIHSS; in the case of elective procedural intubation, serial NIHSS monitoring will end immediately preceding the time of induction/intubation for thrombectomy. Of note, throughout execution of this protocol investigators must ensure that procedures do not delay time to tPA or time to catheterization for mechanical thrombectomy.

Decision Not to Treat: Should a decision not to perform catheter angiography/mechanical thrombectomy occur at the direction of the local neurointerventional surgeon, enrolled patients will complete the positioning intervention phase of the protocol preferably after the time 8 serial NIHSS assessment or earlier if deemed necessary by the attending physician managing the patient. The case report form should be clearly marked with the time of protocol completion, with all NIHSS scores documented out to this terminal time point.

Post-MT Standard of Care: Following the thrombectomy, patients will resume standard of care management for post-thrombectomy patients and can assume whatever head position the stroke team deems appropriate. Standard of care measures typically include serial post-procedural neurological assessments with the NIHSS and strict blood pressure monitoring, however these data will not be analyzed under the ZODIAC protocol. Other standard procedures include obtaining an MRI and other work-up to determine stroke mechanism with assignment of TOAST classification, swallow assessment and advancing diet, application of complication avoidance measures such as venous thromboembolism prophylaxis, and after 24 hours, mobilization procedures. The ZODIAC protocol includes an exploratory 24-hour NIHSS score and a 7 day/discharge NIHSS exploratory assessment by a protocol naïve practitioner certified in the NIHSS. A telephone 3-month mRS exploratory outcome will also be obtained as a standard of care requirement per stroke center certification agencies by a protocol naïve certified practitioner. Note: In enrolled patients not undergoing catheter angiography/mechanical thrombectomy at the direction of the local neurointerventional surgeon, standard of care management will commence once the serial NIHSS monitoring phase of the protocol is complete as described above.

Standard of Care Neuroimaging: All routine imaging (e.g. CT, CTA, MRI, MRA, CXR) occurring throughout the hospitalization must be fully de-identified, blinded to group assignment, copied onto CD, and couriered to the UCLA Core Lab for analysis and archiving independent from the clinical data.

Thrombectomy patients are extremely likely to benefit from 0° positioning because the procedure is only performed in patients with viable yet vulnerable penumbral brain tissue. ZODIAC's novel protocol will capture serial NIHSS over the period before thrombectomy begins, allowing us to better understand how to maintain stability in this high-risk vulnerable population.

Figure: Study Protocol

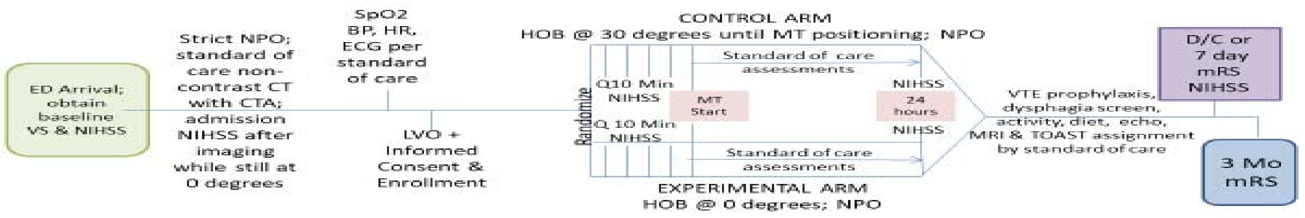

Protocol Fidelity: Table 1 describes the elements of our process evaluation plan.[28-32] This protocol is supported by use of sites led by both fellowship trained vascular neurologists and neurovascular fellowship trained advanced practice nurses that have attained ANVP board certification; collectively, these individuals will oversee the day-to-day management of the protocol. Training manuals and protocol laminated pocket cards include the full protocol, IRB approved/stamped consents, FAQ, troubleshooting, sample case report forms, and PI contact numbers. Site training includes orientation, virtual national meetings, and a 3-month staged pilot protocol implementation with performance feedback/dialogue which will remain ongoing as sites "go live." Data entry hard-stops and text alerts will notify users of documentation errors, and prohibits enrollment of subjects failing to meet inclusion/exclusion criteria. The coordinating center's research coordinator will serve as the clinical site monitor for the trial, independently assessing site performance, protocol compliance, and data entry; reports will be used to generate individual site performance improvement plans as necessary. Study newsletters will disseminate best practices that emerge, share enrollment milestones and highlight sites/providers.

Randomization and power/sample size. Our protocol utilizes two groups, 0°-HOB and 30°-HOB, and allows us to offer enrollment to consecutive eligible patients. To assure balance in the treatment group throughout the course of enrollment, we will use block randomization with a block size of 4 and an allocation ratio of 1:1. This scheme has been implemented in the *Unity* computerized enrollment procedure that only shows the random assignment for a single given participant to the user and may scramble subject enrollment numbers to ensure randomization balance. Future assignments cannot be predicted by study personnel because of the block assignment made together with assignments at all sites, and this effectively mitigates the risk for selection bias that might occur if anticipated group assignment were able to influence enrollment decisions.[33]

Table 1: Process Evaluation Components, Questions, and Indicators[22-26]

Component	Questions	Indicators
Context	Has the standard of care changed to influence adherence or surveillance?<br>Have changes occurred that affect organizational capacity for performance?<br>Have users been exposed to biased messaging that may influence recruitment and/or protocol adherence?	• Consistency of guidelines<br>• CRF structural consistency:<br>o Staff & unit management consistency; staff ratios<br>• Change in screening/enrollment<br>• Feedback from sites
Reach & Recruitment	To what extent is the program reaching intended subjects?<br><br>Are unintended groups enrolled?	• Screening records<br>• Inclusion/exclusion compliance<br>• Differences in intention-to-treat vs. per-protocol treatment of enrolled patients
Implementation:<br><br>Dose Delivered<br><br>Dose Received	To what extent is the program implemented and received?<br><br>How much of the protocol is being administered? What – if anything – is omitted or performed inconsistently?<br><br>What is the average dose received?	• CRF process consistency: Positioning assignment and maintenance; serial monitoring; and, 24-hour, discharge (or 7-day) and 90-day assessments<br>• Neuroimaging enrollment fidelity<br>• Protocol leader oversight records<br>• Feedback from sites
Overall Fidelity (Goal > 90%)	How well does project execution maintain fidelity of the original design?<br>- Aggregate structural compliance<br>- Aggregate process compliance	• CRF structural consistency<br>• CRF process consistency<br>• Blinding of endpoint measurers

We selected individual randomization as opposed to cluster randomization on the hospital/site level for three reasons. First, as emphasized by Murray, "the single most important factor in the power of a group-randomized trial is usually the number of groups."[34] Had we chosen cluster randomization, our sample size would need to increase from n=182, to <u>n=5800</u>, and this would demand <u>at least 29 study sites</u> to efficiently complete the study within 5 years. Given that the largest individually randomized thrombectomy trial included a maximum of 500 patients, and took over 3 years to complete at 16 sites,[32] the conduct of a cluster randomized trial with an even larger thrombectomy sample would require considerable manpower and monetary support, much greater than can be provided. In addition, our 12 geographically widespread sites contribute diverse patients while delivering a consistent standard of care strictly managed by national guidelines, stroke center certification, and core measure requirements.[18-21] Subject diversity is problematic in a cluster randomized design due to an inability to balance important patient traits. In trials involving a small number of clusters (≤10 pairs), the loss of degrees of freedom resulting from pair matching becomes a particularly critical factor.[35] Detailed investigation of this problem has led to the conclusion that for studies with a small number of clusters it is unlikely that effective matching can be possible and that "matching may be overused as a design tool.[36] In contrast, our use of individual (block) randomization will effectively balance patient traits and tPA treatment across protocols, considerably strengthening the evidence derived from this trial. Furthermore, if randomization would be determined on the hospital level, the attending physicians and nurses would know the protocol that would be applied to a patient before that patient is asked to give informed consent. Thus, group-randomization would open our study to enrollment selection bias due to differentially approaching patients that are deemed more/less likely to benefit from the pre-determined protocol for each hospital. This may, in fact, have contributed to enrollment of only small vessel strokes in HeadPoST.[11] Group randomization would also challenge NIHSS/mRS score blinding. In summary,

cluster randomization would be a poor choice due to significant cost, the need for many more enrollment sites, and the oversight manpower required for optimal trial conduct, whereas use of individual randomization provides an economic, feasible and impactful approach to this study.

Data Capture and Control. The UTHSC Center for Biomedical Informatics (CBMI) provides the *RedCap* customized biomedical clinical-research informatics application for data entry. *RedCap* is a fully customizable integrated web-service for data collection with project alerts linked to an encrypted PostgreSQL database and is accessed via the Internet though secure web-applications. Site PIs are required to undergo UTHSC security clearance and training to receive a unique UTHSC ID for access to the *RedCap* system; each site PI will only be able to access data entered directly by them from their site. The study's clinical monitor will verify all data entered from each site against case report forms and electronic health record source data for accuracy; site investigators will be expected to resolve queries about missing data or suspected inaccuracies within 72 hours of notification. At each pre-specified interim analysis milestone, and at the time of the final analysis, research data will be bulk-exported to the ZODIAC biostatisticians who are fully independent from clinical trial enrollment. The national PI will not have access to the study database at any time during or after completion of the trial until an agreed upon post-publication time point.

UTHSC network and computing facilities are owned and managed by UTHSC-ITS; all CBMI systems are housed in the ITS computer center, which has electric power conditioning, UPS battery backup for short-term outages, and a backup diesel generator for long-term outages. The computer center also has fire suppression, temperature and humidity control, card key controlled access and video monitoring. Network traffic crossing the UTHSC network boundary is examined by a Cisco firewall against its Access Control Lists.

Neuroimaging files will be analyzed and archived by the UCLA Neuroimaging Core Lab under the direction of Dr. David Liebeskind. Dr. Liebeskind and his staff will remain fully blinded to patient group assignment; the UCLA team will be responsible for sending all data directly to the ZODIAC statisticians for analysis. While ZODIAC does not incorporate formal imaging-based endpoints, imaging analyses will support protocol fidelity assessment (patient enrollments were consistent with inclusion/exclusion criteria) and will also aid in understanding differences found between groups once the study is complete.

Endpoints.

Primary Endpoint: The percentage of patients with early neurologic deterioration (END) is our primary outcome. END is defined as a two or more point worsening (≥ 2) in the NIHSS score in comparison to the time-0 NIHSS score at the time of positioning intervention termination (placement of the patient on the catheterization table or immediately prior to the time of elective induction/intubation for thrombectomy in centers utilizing general anesthesia). Not deteriorating (being stable) is defined as less than 2-point NIHSS worsening (< 2) or NIHSS score improvement in comparison to the time-0 NIHSS score during the head position intervention.

We expect to see at most 5% of END participants in the 0° head position group, compared to 20% of END patients in the 30° head position group (15% lower deterioration). Our group-sequential design allows for early stopping due to futility or efficacy and is based on a two-sample two-sided proportion test as implemented in East 6.0 (Cytel, Cambridge, MA) with type I error alpha=0.05 and 80% power; it incorporates 3 interim analyses and 1 final look at the data which results in a sample size of n = 182. Interim analyses are planned with equal spacing after cases have been enrolled/observed for the primary endpoint (Table 2). The Lan-Demets alpha and beta spending approach with O'Brien-Fleming

boundaries[37] are used both for futility and efficacy interim monitoring. Absence of primary endpoint evaluation will be counted as an END event so that all randomized participants will have a determined primary endpoint available for analysis, allowing for an intent-to-treat analysis with no primary endpoint attrition.

Analysis Plan for Aim 1 (primary efficacy endpoint); *Hypothesis*: *Patients with large artery occlusions placed in a 0°-HOB position will experience less early neurological deterioration than those in the 30°-HOB elevation group.* We will apply sequential testing in both patient groups (Table 2). As an example, the first look in the protocol is performed when 46 participants are enrolled/have their primary outcome evaluated; if the z-value associated with the test of equality of proportions in both arms is above 4.333 or below -4.333 the trial will be stopped early for efficacy (one group is clearly superior with respect to the primary outcome), whereas if that z-value is in the interval (-0.007, 0.007), the trial will be stopped early for futility (groups are essentially identical in outcome). If the z-value falls within the intervals (-4.333, -0.007) or (0.007, 4.333), the trial will continue until the next look at the data. Note that for the primary endpoint, absence of evaluation is counted as deterioration and that, consequently, all randomized participants will have a determined primary endpoint available for the analysis; therefore, intent-to-treat principles will support our primary data analysis. We will use unadjusted Cox proportional-hazards modeling to compare and Kaplan-Meier curves to visualize the rate of early neurological deterioration (> 2 NIHSS points change from baseline) in the two groups. The number at risk and censored in each group will be reported in the Kaplan-Meier survival curves every 10 minutes. Overall rates of END and according to group will be reported as frequencies and percentages and compared between groups with Fisher's Exact test. The number needed to harm will be estimated as the inverse of the absolute risk increase (ARI) = 1/ARI.

Table 2: Stopping Boundaries for the Sequential Design.

Look #	Sample Size	Stopping Boundaries (Extended Protocol)			
	MT Subjects	Efficacy Z (lower, upper)		Futility Z (lower, upper)	
1	46	4.333	-4.333	0.007	-0.007
2	92	2.963	-2.963	0.374	-0.374
3	138	2.359	-2.359	1.261	-1.261
4	182	2.014	-2.014	2.014	-2.014

Analysis Plan for Process Evaluation. Most of the process evaluation components (Table 1) naturally render themselves to description and categorization. Noteworthy exceptions are aspects relating to intent-to-treat vs. per-protocol received treatment, and control of head positioning assignment. The data collected will enable generation of statistical quality control charts[38-39] if necessary, to monitor the proportion of non-conforming patients (delivered treatment different from assigned protocol) over time. Should control charting be necessary, they will be assembled by ZODIAC independent biostatisticians and provided to Drs. Brewer and Middleton (the study's protocol fidelity and implementation science experts) as an ongoing monitoring effort. The upper and lower control levels of these charts will allow a ready assessment of whether deviations over time or between hospitals are coincidental or driven by some real difference or shift in hospital procedures.

Analysis Plan for Aim 2 (secondary aim assessing safety). Serious adverse events (SAE) that will be monitored closely in this trial include: 1) Severe neurological deterioration; 2) hospital acquired pneumonia; and, 3) death. Analyses will be descriptive and include rates for SAEs by study cohort.

Severe neurological deterioration (SND) is defined as at least a 4-point increase (≥4) in the NIHSS from the time 0 NIHSS baseline. Our NIHSS serial assessments will allow for early detection of neurologic change in our subjects should it occur. SND is a severe form of deterioration, and consequently, our provision for early trial stopping is a safety measure. Because SND can be due to a number of different factors that may or may not be associated with this protocol, incidents of SND will also be reviewed by the Data Safety Monitoring Board (DSMB)[40] as described below, and adjudicated to the intervention or other factors as indicated by their findings.

Hospital acquired pneumonia (HAP). The American Thoracic Society/Infectious Diseases Society of America (ATS/IDSA) guidelines[41] will be used, defining pneumonia as requiring onset of a new or progressive infiltrate on pulmonary imaging within 72 hours, along with at least two of the following: 1) Fever $\geq 38^{o}$ C; 2) purulent sputum; 3) leukocytosis or leukopenia; and/or, 4) decline in oxygenation. Based on our pilots, we are excluding cases from enrollment that are high risk for pneumonia; because these cases are kept NPO for thrombectomy, this will further reduce aspiration risk. We therefore believe that pneumonias will be rare and because of this, we believe that exposure to unnecessary additional chest imaging would add both increased cost and unnecessary radiation exposure, especially since stroke patients often require repeat neuroimaging. Instead, excellent nursing assessments will serve as triggers to inform the need for additional chest imaging in the case of pulmonary deterioration. Because pneumonia may be due to a number of different unassociated factors, pneumonia events will be DSMB reviewed and adjudicated to the intervention as indicated.

Death. Death occurring during the active protocol phase, throughout hospitalization, or within 3 months from enrollment will be monitored, and incidents adjudicated by the DSMB. Death may be associated with a number of unassociated factors, therefore the DSMB will carefully determine study association.

Other AEs detected will be DSMB reviewed and reported as related or unrelated to study procedures.

All the safety outcome analyses will be executed in the intention-to-treat population. We will use unadjusted Cox proportional-hazards modeling to compare and Kaplan-Meier curves to visualize the rate of severe early neurological deterioration (> 4 NIHSS points change from baseline) in the two groups. The number at risk and censored in each group will be reported in the Kaplan-Meier survival curves every 10 minutes. Overall and enrollment group rates of SND will be reported as frequencies and percentages and compared between groups with Fisher's Exact test. The number needed to harm for SND will be estimated as the inverse of the absolute risk increase (ARI) = 1/ARI. Rates of hospital-acquired pneumonia, discharge, 90-day participant's death, post-discharge stroke, and symptomatic intracerebral hemorrhage post-reperfusion treatment will be reported in frequencies and percentages; these will be compared between groups using Fisher's Exact test together with odds ratios and the corresponding 95% confidence intervals and p-values from unadjusted logistic regression models that include randomization assignment as the predictor.

Pre-Specified Exploratory Endpoints: As discussed, END is the primary endpoint for this clinical trial and is measured proximally, at the termination of the intervention monitoring period. Our rationale for not utilizing a post-thrombectomy 90-day outcome is twofold: First, head positioning should not be deemed a "treatment" for LVO stroke; instead, head positioning is a rescue procedure similar to other medical care that is utilized to stabilize, optimize, and ensure both patient safety and optimal outcomes. The impact of a rescue procedure such as head positioning is best measured during the time it is implemented to determine its efficacy in supporting and optimizing patient condition. Second, thrombectomy is a highly effective definitive treatment for large vessel occlusion stroke patients. The tremendous effect size of thrombectomy may likely override any benefit of other interventions, including

head positioning, however this remains unknown at this time. In summary, we aim to determine the efficacy of head positioning as a simple rescue procedure, making measurement of END at the time of positioning on the catheterization lab table for thrombectomy (or immediately prior to elective induction/intubation in sites using general anesthesia for thrombectomy) our choice as the study primary endpoint.

That said, we have identified several pre-specified exploratory endpoints that can be used to examine whether any continued benefit from head positioning occurs in combination with thrombectomy treatment. Specifically, the ZODIAC study will examine the following exploratory endpoints for between group differences:

- 24-hour NIHSS scores
- 7 day or discharge (whichever occurs first) NIHSS scores
- 7 day or discharge (whichever occurs first) mRS
- 90-day mRS

We will examine improvement rates at 24 hours and again at discharge/7 days (whichever comes first) in the NIHSS and will report this as frequencies and percentages; these will be compared between groups using Fisher's Exact test together with odds ratios and the corresponding 95% confidence intervals and p-values from unadjusted logistic regression models that include randomization assignment as the predictor. Changes from baseline NIHSS for individual subjects will be plotted by means of a linear plot according to their randomization group to visualize the changes in NIHSS after randomization in each group; a repeated measures ANOVA will be employed to compare the serial NIHSS measures between the two groups. The 90-day mRS will be analyzed in 3 ways: 1) Dichotomized as 0-2/3-6 and 0-3/4-6 and modeled as a dependent variable by unadjusted logistic regression with randomization assignment as the predictor. The 90-day mRS will be plotted using a paired horizontal bar graph (aka "Grotta bars") for visualization; 2) Analyzed as separate categories and modeled by unadjusted ordinal logistic regression as a dependent variable and with randomization assignment as the predictor. The proportional odds assumption will be verified by likelihood ratio testing; and 3) Transformed to a utility-weighted mRS (UW-mRS)[42] where each of the mRS categories (from 0 to 6) is assigned a weight (1.00, 0.91, 0.76, 0.65, 0.33, 0.00 and 0.00) and modeled as a dependent variable by unadjusted linear regression, with randomization assignment as the predictor.

Of note, ZODIAC is not powered for these exploratory analyses, so interpretation of any findings from these analyses much take this into account.

Data Safety Monitoring Plan

Monitoring Entity: Data Safety Monitoring Board (DSMB). A DSMB has been established to support safety monitoring for this study and will consist of 5 voting members (including the Chair) who are researchers with expertise in clinical trials, imaging, hemodynamics, neurovascular disease and ethics in relation to human subjects research that are external to and independent from any enrolling study team. The PI will work closely with the DSMB to facilitate their expressed needs for making important decisions about any progressively accumulated ethical and efficacy evidence.

Roles and Responsibilities of the DSMB. Dr. Tsivgoulis will serve as Chairman of the DSMB and will work directly with ZODIAC's independent biostatisticians to facilitate data access and obtain any information that may be required from collected data. Dr. Tsivgoulis has substantial expertise in biostatistics as well as vascular neurology, and has led numerous international studies overseeing data management and analyses, and therefore is well suited to lead our DSMB in its examination of intervention efficacy, futility, and safety. He will assume responsibility for the development and submission of all safety reporting to the PI, Institutional Review Boards (IRB), and the NIH, to ensure transparency of our work. Specifically, Dr. Tsivgoulis will develop each agenda for DSMB meetings, conduct meetings using Robert's Rules of Order, chair open, closed and executive sessions, and ensure that meeting summaries and final minutes are adequately prepared and approved as appropriate. Dr. Tsivgoulis will act as the primary contact person for the DSMB. He will be responsible for setting meeting dates and contacting new and, if indicated, ad hoc members to assess their content expertise and orient them to the DSMB process.

The other members of our DSMB will serve as expert objective reviewers of all findings presented to them, and work with Dr. Tsivgoulis to determine the need for additional data or protocol changes as indicated. Full DSMB membership is listed in Table 3. Ms. Erwin Davison, MBA will serve as an ex-officio member of the DSMB in the role of Executive Secretary; her role includes ensuring ongoing compliance with all DSMB members' Human Subjects training, Conflict of Interest (COI) trainings, and COI form completion, ensuring COI forms are approved, working with Dr. Tsivgoulis to establish meeting dates, and working with Dr. Tsivgoulis to procure and assemble required information for each meeting. Ms. Davison will ensure distribution of meeting materials in advance of each meeting to ensure there is adequate time to review materials. She will take notes during open, closed, and executive session of each meeting or during each conference call so that final draft minutes and written meeting summaries can be prepared. Ms. Davison will forward all meeting summaries for DSMB approval within 5 days of each meeting, and she will maintain all protocol documents, COI forms, data, final minutes and meeting summaries from each DSMB meeting in a locked file cabinet within her locked office.

Table 3: DSMB Membership and Roles

DSMB Member	Role	Expertise
Georgios Tsivgoulis, MD, PhD<br>Vascular Neurologist<br>National & Kapodistrian<br>University of Athens	DSMB Chairman<br>Voting Member	Vascular Neurology<br>Clinical Trials<br>Biostatistics<br>DSMB Expert
Erwin Davison, MBA<br>UTHSC Grants Manager	Executive Secretary<br>Ex-officio Member<br>Non-Voting Member	Clinical Trials<br>Research Ethics<br>Grant Reporting
James Rhudy, PhD, DNP, RN<br>Neurology Research Professor	DSMB Member<br>Voting Member	Human Subjects<br>Research Ethics<br>Clinical Trials
Aristeidis Katsanos, MD<br>Vascular Neurologist<br>McMaster University	DSMB Member<br>Voting Member	General Neurology<br>Vascular Neurology<br>Biostatistics<br>Clinical Trials
Joshua Lennon, MD<br>Private Practice Neurologist<br>Regional One Medical Center	DSMB Member<br>Voting Member	Vascular Neurology<br>Sleep Medicine<br>Clinical Trials
Vijay Sharma, MD<br>Vascular Neurologist<br>National University Hospital of Singapore	DSMB Member<br>Voting Member	DSMB Expert<br>General Neurology<br>Vascular Neurology<br>Clinical Trials

All members of ZODIAC's DSMB are independent of this study and not affiliated with any clinical institution that is enrolling in this clinical trial. Additionally, none of the above DSMB members participated in protocol development for this study, nor do they supervise persons who are involved in this study. All DSMB members have completed a COI form in relation to this proposed clinical trial which documents no conflicts of interest with this study. Overall responsibilities of the DSMB members include, 1) the protection of study participants from exposure to unreasonable or unnecessary research risks by monitoring trial data for effectiveness and safety, 2) review of interim data in the context of the most recent scientific literature with the authority to unmask data as deemed necessary, 3) ensuring clinical studies do not continue beyond the point when the objectives have been met and a clinically meaningful

answer of importance to the scientific community and the public has been obtained, and 4) the monitoring of study progress and conduct. Specifically, responsibilities and functions of the DSMB will include:

- Approval of the study protocol, review plans for data and safety monitoring, the informed consent, reporting templates for data presentations, and other items deemed important to review and approve prior to study commencement.
- Establishing guidelines for safety monitoring, including a list of events that should be reported immediately and the format for cumulative reporting at specific intervals.
- Review interim analyses of outcome data, including allowance of unmasking of blinded data for efficacy and futility assessment at pre-specified intervals.
- Review of “toxicity” data to include serious adverse events (SAE), and adverse events (AE), and making recommendations as indicated on whether the trial should continue as originally designed, be revised, suspended or terminated based on observed beneficial or adverse effects related to study interventions.
- Assess trial performance information including recruitment, retention, resource center performance, follow-up information, and listings of protocol violations.
- Review published reports of related studies submitted by the study investigators or DSMB members to determine whether the study needs to be revised or terminated.
- Review proposed modifications to the study prior to their implementation and make recommendations to the PI and/or IRB.
- Review proposed stopping guidelines as specified in the protocol and, at its discretion, recommend modification to the proposed plan or propose a plan if none has been proposed.
- Provide advice and feedback on data analysis to the study statistician or study monitor.
- Monitoring differences in site performance that may warrant site remediation. The DSMB will examine data by enrolling site to determine important trends in events tied to protocol completion and/or patient outcome; findings will be presented in formal reports when issues arise with any enrolling site that requires remediation. As soon as possible, but within 20 days following each DSMB meeting, a written summary will be provided along with justifications related to any recommendations for continuing, changing or terminating the trial. This will be provided along with a statement concerning the impact on the trial of individually observed or cumulative SAEs and AEs as indicated.
- Ensuring the confidentiality of all participant study data used to determine protocol safety.

<u>Role of the PI and Research Coordinator in Safety Monitoring</u>. The PI will hold the ultimate responsibility to report issues related to protocol safety events, as well as concerns about research protocol and data integrity to the DSMB, local and UTHSC IRBs, and NIH. Specifically, the PI will provide written reports to the DSMB on the current status of the trial, interim analyses, adverse events, and problems encountered. The PI’s report may contain recommendations for consideration by the DSMB concerning clinical site performance, whether to continue accrual and/or follow up, whether to close the trial, and whether the results should be reported. The PI will also take responsibility for amending the protocol in accordance with DSMB recommendations and notifying the clinical site and IRBs as expeditiously as possible. The PI will provide the DSMB with any modifications to the study prior to their implementation, and she will forward DSMB recommendations and meeting minutes as appropriate to the IRB or other clinical research sites involved. The PI will also provide timely de-identified reports to NIH of unanticipated problems or unexpected SAEs, IRB-approved revisions to the study protocol that indicate a change in risk for participants, summarized recommendations made by the DSMB as appropriate along with an action plan for response, and she will also provide notice of any actions taken

by the IRB regarding the research and responses to these actions. The ZODIAC Research Coordinator will function as a site monitor, auditing 100% of enrolled patients' case report forms (CRFs) alongside source documents and examining informed consent procedures.

DSMB Meetings. DSMB meeting frequency will depend on scheduled meetings and also special called meetings that occur because of an SAE, AE, or other safety related event. The DSMB will meet at least once annually should no special called meetings be necessary. Additionally, meetings and/or conference calls may be held at the request of DSMB members, the study leadership, the NIH Program Director or designee, or the local IRB or UTHSC. All meeting materials provided to the DSMB are considered privileged and confidential and will be watermarked as such by the Executive Secretary. Confidentiality of DSMB materials will be maintained at all times to the extent permitted by law.

Meetings of the DSMB will be divided as follows:

1) Open Session – which may include members of the clinical trial team, NIH staff, and ad hoc members at the direction of the DSMB Chair for the purpose of general trial conduct and progress discussions including "toxicity" issues (SAEs, AEs), subject accrual, protocol compliance, site performance, quality control, follow-ups, or other general items. No confidential data will be shared during open sessions of the DSMB, and blinding will be maintained during open sessions.

2) Closed Session – during this session, attendance will be limited to voting members, any ex-officio members invited by the Chair, and the Executive Secretary; NIH staff may be invited to attend this portion of the meeting but will act strictly as observers and will not participate in deliberations or provide additional information that may influence recommendations. Information discussed during closed sessions includes aggregated safety data, efficacy, and futility data – including unmasking of blinded data presented by the statistician if necessary to assure participant safety. NIH staff are not privy to post-randomization data broken down by treatment groups that may be discussed.

3) Executive Session – Only voting members and the Executive Secretary may be present in an executive session. Members will discuss the general conduct of the trial, outcome results including SAEs and AEs and implications. Should the Chair ask the Executive Secretary to excuse herself, he will then assume responsibility for recording minutes and recommendations of this session. The Chair may break the blind if this is deemed necessary to make decisions about efficacy or futility. Recommendations coming out of executive session will include A) study continuation, B) study termination, C) study suspension, and, D) the need for study protocol revisions. At the Chair's discretion, NIH staff may be invited to attend, but may only be observers in the process and are not privy to unmasking of blinded data. The DSMB will vote on recommendations in executive session and efforts will be made to obtain a consensus. If consensus cannot be obtained, a majority vote will carry a recommendation. The Chair will participate in discussions and will vote. Should a minority opinion be present, this will be reported alongside the recommendation. The Executive Secretary (or Chair if the Executive Secretary is excused) will document the discussion and recommendations. The final recommendations must be summarized either as majority or minority positions or as actual vote tallies for the various divergent recommendations.

Written Meeting Summaries of DSMB Recommendations. A written meeting summary that identifies topics discussed by the DSMB describing individual findings, overall safety assessment and recommendations with justification related to continuing, changing, suspending, or terminating the trial and the impact on the trial of individually observed or cumulative adverse events will be signed by the Chair. This report should contain no mention of safety, efficacy or futility data by treatment group. Written meeting summaries must be submitted to NIH and the local IRB within 14 days of meeting occurrence.

In the absence of disagreement, the PI must act to implement the recommendations as expeditiously as possible by amending the protocol. Should the PI disagree with the DSMB, she will be responsible for reaching a mutually acceptable decision about the study with the IRB. Meeting minutes will be prepared, and a final draft version will be signed/approved by the DSMB Chair. Minutes should include: 1) General highlights of the discussion; 2) general recommendations; 3) actionable items; 4) suggested protocol/study changes and rationale for each; and, 5) the date for the next scheduled meeting of the DSMB. Minutes will not contain confidential data.

Release of Outcome Data. Confidential outcome data should not be made available to individuals outside of the DSMB. Any release of outcome data to individuals outside the DSMB must be reviewed and approved by the DSMB, the local IRB, and the PI.

Confidentiality Procedures. No communication, either written or oral, of the deliberations or recommendations of the DSMB will be made outside of the DSMB except as provided for in these guidelines. Outcome results are strictly confidential and must not be divulged to any non-member of the DSMB.

Procedures for Monitoring, Minimizing Risk, and Protecting Confidentiality of Participant Data

Procedures for Monitoring Study Safety. The DSMB will follow a standard monitoring schedule and also hold special called monitoring meetings based on the occurrence of an SAE or other safety, IRB compliance, or intervention performance concerns that may arise. The standard monitoring schedule for the DSMB is determined by enrollment milestones. A total of 4 “looks” at enrollment data will be scheduled with “look 1” occurring after the first 46 patients are enrolled, “look 2” after 92 patients, “look 3” after 138 patients, and “look 4” – the final look – after all 182 patients are enrolled (Table 2).

The PI will hold responsibility for notifying the DSMB Chair and IRB Chair of the occurrence of any SAE (as previously defined) and this will trigger a special called meeting of the DSMB. PI notification to the DSMB Chair, IRB and NIH will occur within 24 hours of SAE occurrence. The DSMB Chair will hold authority for assembling data for review by the full DSMB on all SAE cases. Each SAE case will be adjudicated as either “associated with the intervention” or “unassociated with the intervention” by the DSMB, and this determination will be reported by the DSMB Chair formally in writing to the PI, the IRB, and NIH. The FDA standards for “serious,” “anticipated” and “associated with the treatment” will be employed. The DSMB will follow SAE occurrences adjudicated to the intervention for trends suggestive of harm that may cause the study to be suspended, revised or terminated, and will independently hold the authority to stop the trial based on such a determination.

The DSMB will audit select cases to ensure compliance with IRB requirements. The ZODIAC research coordinator will work as the clinical monitor and will be authorized to audit all CRFs and source documents. All CRFs will be visible to the research coordinator online through the web-based enrollment system, with auto-notification sent from the system for each enrollment. Site visits will be triggered by enrollment numbers, so that for every 3 cases enrolled, the research coordinator will visit the site to further examine:

- Investigators’ compliance with protocol and IRB requirements;
- Sites conformance with informed consent requirements; and,
- Verification of source documents supporting the CRF data documented.

Sites determined to be out of compliance will be reported by the research coordinator to the PI. In turn, the PI will notify the local IRB and UTHSC IRB, as well as the Chair of the DSMB. Depending on the

infraction identified, the PI, the DSMB Chair and the UTHSC IRB Chair will determine the appropriate course of action which may include site termination.

Procedures for Minimizing Research-Associated Risk. The following procedures will be employed within the study protocol and by the DSMB to ensure study safety:

- Study protocol exclusions to ensure safety. Preliminary work supporting this study has examined protocol safety for head positioning interventions in detail to determine patients that should be included, as well as those that should be excluded because of safety risks. Our inclusion and exclusion criteria reflect this information. Specifically, our exclusions include patients who may be at undue risk due to laying flat, as well as those with concurrent diagnoses or clinical findings that may confound our ability to understand protocol safety.

- DSMB procedures to ensure safety. Procedures to ensure safety utilized by the DSMB will include approval of the study protocol and the informed consent, establishing a guideline for safety monitoring that includes a list of events that should be reported immediately, revieweing data from planned interim analyses, reviewing toxicity data that includes SAEs and AEs to determine if these events are intervention and trial related, and deciding on the need for study method revisions, study suspension and study termination based on findings. As mentioned previously, the PI will alert the DSMB of safety incidents warranting further evaluation. The research coordinator will monitor protocol compliance and notify the PI of sites with compliance concerns which the PI will in turn report to the DSMB Chair, the IRB and NIH. The DSMB Chair will convene special called meetings to address safety incidents and protocol compliance. The DSMB will hold planned meetings to review findings from efficacy and futility analyses when enrollment milestones have been met. The DSMB will also assess overall trial performance information including recruitment, retention, resource center performance, follow-up information, and listings of protocol violations. The DSMB will also be charged with reviewing proposed modifications to the study prior to their implementation and make recommendations about proposed changes to the PI and/or IRB as indicated. The DSMB and study investigators will also stay abreast of new findings in practice that may call for a different approach. Collectively, these methods will minimize any undue risk in our enrolled patients. Safety decisions involving the need to suspend or terminate the study will be communicated immediately (within no more than 24 hours) by the DSMB to the PI, the IRB, and NIH.

Procedures for Protecting the Confidentiality of Patient Data. The work of the DSMB will be supported by strict confidentiality. All DSMB members will complete COI forms and be vetted for inclusion on the monitoring board. Confidential outcome data reviewed by the trial will not be made available to individuals outside of the DSMB, and any release of outcome data to individuals outside the DSMB must be reviewed and approved by the DSMB, the local IRB, and the PI. Additionally, no communication, either written or oral form, of the deliberations or recommendations of the DSMB will be made outside of the DSMB except as provided for in these guidelines. Outcome results are strictly confidential and will not be divulged to any non-member of the DSMB without the expressed approval of the PI, the IRB and NIH.

In accordance with UTHSC IRB and NIH requirements, the ZODIAC protocol will utilize methods that ensure the confidentiality of our data. The UTHSC Center for Biomedical Informatics (CBMI) has

provided the web-based customized biomedical clinical-research informatics application for enrollment, randomization, and data entry. Our web-based data system is a fully customizable integrated service for data collection and project alerts linked to an encrypted PostgreSQL database, and is accessed via the Internet though secure web-applications. The UTHSC network and computing facilities are owned and managed by UTHSC-ITS; all CBMI systems are housed in the ITS computer center, which has electric power conditioning, UPS battery backup for short-term outages, and a backup diesel generator for long-term outages. The computer center also has fire suppression, temperature and humidity control, card key controlled access and video monitoring. Network traffic crossing the UTHSC network boundary is examined by a Cisco firewall against its Access Control Lists. Only the research coordinator and ZODIAC's independent biostatisticians are able to review data within the system. Authority for use will be strictly monitored to ensure that system use is appropriate.

Procedures for Identifying, Reviewing, and Reporting Adverse Events and Unanticipated Problems to IRB and NIH

Safety is a secondary aim of this study and will be examined in this larger, more generalizable sample of patients. Specifically, SAEs that will be monitored closely in this trial include: 1) Severe neurological deterioration (SND) defined as a deterioration of $\geq$ 4 points on the NIHSS from the time-0 NIHSS; 2) hospital acquired pneumonia (HAP) as defined by the American Thoracic Society/Infectious Disease Society of America;[41] and, 3) death. As SAEs occur, the DSMB, IRB and NIH will be notified by the PI. The DSMB Chair will convene a special called meeting (as described previously) to determine whether the SAE should be adjudicated to the intervention or other unrelated cause. The DSMB will follow the methods for meeting conduct as described previously in this document, along with the written report methods specified. Beyond individual SAE review, the DSMB will examine analyses as directed by the statistical plan. The DSMB will formally report their findings to the PI, IRB and NIH.

Procedures to Ensure Monitoring Plan Compliance and Reporting Requirements for Study Sites

All regular meetings of the DSMB will include discussion of individual and aggregate site performance, including adherence to all aspects of the study protocol, completeness of CRFs, accuracy of source data in relation to CRF completeness, compliance of informed consent procedures, and trends noted in any of these areas. As described previously, the research coordinator will monitor 100% of CRFs submitted on patients. The web-based CRF system automatically flags required fields so that deficiencies are easily detected. After every 3 cases, the research coordinator will travel to a site to examine source documents in relation to CRFs. All deficiencies noted by the research coordinator will be reported to the PI, and the PI in turn will notify the DSMB when protocol compliance concerns arise. The DSMB will evaluate individual site compliance but also will aggregate site compliance issues as needed, so that they may identify if multiple sites are non-compliant with certain aspects of the protocol, as such findings may indicate an unrealistic approach to study methods that may require protocol revision. The DSMB will notify the PI, IRB and NIH of compliance findings in individual sites and by aggregate, and will recommend actionable measures to improve compliance that may include site remediation, or site termination as indicated by the infraction.

Procedures for Assessment of External Factors or Relevant Information that may Influence Study Participant Safety

All investigators and members of the DSMB hold responsibility for scanning the practice environment, keeping current with standards of care, possessing knowledge of evolving care standards, and reviewing related research findings that may impact the safe enrollment of subjects in this study. In particular, factors that may impact the receipt of timely standard of care reperfusion treatments will be evaluated in

an ongoing manner to ensure that study participants are not disadvantaged by participating in this study protocol.

Plans for Interim Efficacy and Futility Analyses

ZODIAC is a efficacy study (Aim 1) that will determine superiority of one of two head positions in patients with large vessel occlusion hyperacute ischemic stroke prior to commencement of thrombectomy. Because the primary endpoint of clinical stability in and of itself constitutes a safety endpoint, we have developed a plan to examine both protocol efficacy and protocol futility with defined stopping boundaries (Table 2 repeated below). Using sequential testing, the DSMB will take a first look at data when 46 participants are enrolled and have had their primary outcome evaluated. For example, the DSMB will stop the trial for efficacy (with one group clearly superior) if the z-value associated with the test of equality of proportions is above 4.333 or below -4.333, whereas if that z-value is in the interval (-0.007, 0.007), the DSMB will stop the trial early for futility. However, if the z-value falls within the intervals (-4.333, -0.007) or (0.007, 4.333), the DSMB will continue the trial until the next look at the data when 92 participants are available. As described previously under the meetings section of this plan, the DSMB will hold regularly scheduled meetings based on achievement of these Table 2 enrollment milestones, and continue to assess efficacy and futility at each of these meetings until the point of trial completion is determined. The DSMB will formally report these findings to the PI, IRB, and NIH using the written report methods described above.

Table 2: Stopping Boundaries for ZODIAC's Sequential Design.

Look #	Sample Size	Stopping Boundaries			
	MT Subjects	Efficacy Z (lower, upper)		Futility Z (lower, upper)	
1	46	4.333	-4.333	0.007	-0.007
2	92	2.963	-2.963	0.374	-0.374
3	138	2.359	-2.359	1.261	-1.261
4	182	2.014	-2.014	2.014	-2.014

Lastly, the PI will collaborate with the DSMB, the IRB and NIH to modify this DSMP to address additional concerns or issues that may arise. Therefore, this plan may be subject to change as the protocol is implemented; any changes to this plan will be immediately submitted to NIH and our IRBs.

REFERENCES

1. Wojner AW, El-Mitwalli A, Alexandrov AV. Effect of head positioning on intracranial blood flow velocities in acute ischemic stroke: A pilot study. *Critical Care Nursing Quarterly*. 2002;24(4):57-66.
2. Wojner-Alexandrov AW, Garami Z, Chernyshev OY, Alexandrov AV. Heads down: Flat positioning improves blood flow velocity in acute ischemic stroke. *Neurology*. 2005;64(8):1354-1357.
3. Hunter AJ, Snodgrass SJ, Quain D, Parsons MW, Levi CR. HOBOE (Head-of-Bed Optimization of Elevation) Study. *Physical Therapy*. 2011;91(10):1503-1512.
4. Ali LK, Weng JK, Starkman S, Saver JL, Kim D, Ovbiagele B, Buck BH, Sanossian N, Vespa P, Bang OY, Jahan R, Duckwiler GR, Vinuela F, Liebeskind, DS. Heads Up! A novel provocative maneuver to guide acute ischemic stroke management. *Interventional Neurology*. 2017;6(1-2):8-15.
5. Durduran T, Zhou C, Yu G, Edlow B, Choe R, Shah Q, Kasner SE, Cucchiara BL, Yodh AG, Greenberg JH, Detra JA. Bedside monitoring of cerebral blood flow in acute stroke patients during changes in head of bed position. *Stroke*. 2007; 38(2):494.
6. Toole, JF. Effects of change of head, limb and body position on cephalic circulation. *New England Journal of Medicine*. 1968;279(6):307-311.
7. Caplan LR, Sergay S. Positional cerebral ischaemia. *Journal of Neurology, Neurosurgery and Psychiatry*. 1976;39(4):385-391.
8. Hayashida K, Hirose Y, Kaminaga T, Ishida Y, Imakita S, Takamiya M, Yokota I, Nishimura T. Detection of postural cerebral hypoperfusion with technetium-99m-HMPAO brain SPECT in patients with cerebrovascular disease. *Journal of Nuclear Medicine*. 1993;34(11):1931-5.
9. Ouchi Y, Nobezawa S, Yoshikawa E, Futatsubashi M, Kanno T, Okada H, Torizuka T, Nakayama T, Tanaka K. Postural effects on brain hemodynamics in unilateral cerebral artery occlusive disease: A positron emission tomography study. *Journal of Cerebral Blood Flow and Metabolism*. 2001;21(9):1058-1066.
10. Hargroves D, Tallis R, Pomeroy V, Bhalla A. The influence of positioning upon cerebral oxygenation after acute stroke: A pilot study. *Age and Ageing*. 2008;37(5):581-585.
11. Anderson CS, Arima H, Lavados P, Billot L, Hackett, ML, Olavarria VV, Munoz Venturelli P, Brunser A, Peng B, Cui L, Song L, Rogers, K, Middleton S, Lim JY, Forshaw D, Lightbody CE, Woodward M, Pontes-Neto, O, De Silva, HA, Lin R-T, Lee, TH, Pandian JD, Mead GE, Robinson T, Watkins C, for the HeadPoST Investigators and Coordinators. Cluster-randomized, crossover trial of head positioning in acute stroke. *NEJM*. 2017;376:2437-47.
12. Alexandrov AW, Tsivgoulis G, Hill MD, Liebeskind DS, Schellinger P, Ovbiagele B, Arthur A, Caso V, Nogueira R, Hemphill JC, Grotta JC, Hacke W, Alexandrov AV. HeadPoST: Rightly positioned, or flat out wrong? *Neurology.* 2018;90(18):885-889.
13. American Heart Association News. Head position after stroke: Up or down? *American Heart Association*, International Stroke Conference News Stories. Blog, International Stroke Conference 2017, February 22, 2017.
14. Fiore K. Lying flat, sitting up equal for mild stroke recovery. *Medpage Today*. www.medpagetoday.com/MeetingCoverage/ISC/63345?xid=nl_mpt_cardiodaily_2017-02-22&eun=g1084220d0r. Accessed September 15, 2017.
15. Diener H-C. Flawed studies define this year's International Stroke Conference. *Medscape Neurology*. March 7, 2017.
16. Grotta JC, Welch KM, Fagan SC, Lu M, Frankel MR, Brott T, Levine SR, Lyden PD. Clinical deterioration following improvement in the NINDS rt-PA Stroke Trial. *Stroke*. 2001;32(3):661-668.
17. Davalos A, Toni D, Iweins F, Lesaffre E, Bastianello S, Castillo J. Neurological deterioration in acute ischemic stroke: Potential predictors and associated factors in the European Cooperative Acute Stroke Study (ECASS) I. *Stroke*. 1999;30(12):2631-2636.

18. Jauch EC, Saver JL, Adams HP Jr, Bruno A, Connors JJ, Demaerschalk BM, Khatri P, McMullan PW Jr, Qureshi AI, Rosenfield K, Scott PA, Summers DR, Wang DZ, Wintermark M, Yonas H; American Heart Association Stroke Council; Council on Cardiovascular Nursing; Council on Peripheral Vascular Disease; Council on Clinical Cardiology. Guidelines for the early management of patients with acute ischemic stroke: a guideline for healthcare professionals from the American Heart Association/American Stroke Association. *Stroke*. 2013 Mar;44(3):870-947
19. Powers WJ, Derdeyn CP, Biller J, et al. 2015 AHA/ ASA Focused Update of the 2013 Guidelines for the Early Management of Patients With Acute Ischemic Stroke Regarding Endovascular Treatment: a Guideline for Healthcare Professionals From the American Heart Association/American Stroke Association. *Stroke*. 2015;46(10):3020-3035.
20. Summers D, Leonard A, Wentworth D, Saver JL, Simpson J, Spilker JA, Hock N, Miller E, Mitchell PH. American Heart Association Council on Cardiovascular Nursing and Stroke Council. Comprehensive overview of nursing and interdisciplinary care of the acute ischemic stroke patient: A scientific statement from the American Heart Association. *Stroke*. 2009;40(8):2911-2944.
21. *The Joint Commission*. www.jointcommission.org/certification/certification_main.aspx. Accessed September 15, 2017.
22. Berkhemer OA, Fransen PS, Beumer D, van den Berg LA, Lingsma HF, Yoo AJ, Schonewille WJ, Vos JA, Nederkoorn PJ, Wermer MJ, van Walderveen MA, Staals J, Hofmeijer J, van Oostayen JA, Lycklama à Nijeholt GJ, Boiten J, Brouwer PA, Emmer BJ, de Bruijn SF, van Dijk LC, Kappelle LJ, Lo RH, van Dijk EJ, de Vries J, de Kort PL, van Rooij WJ, van den Berg JS, van Hasselt BA, Aerden LA, Dallinga RJ, Visser MC, Bot JC, Vroomen PC, Eshghi O, Schreuder TH, Heijboer RJ, Keizer K, Tielbeek AV, den Hertog HM, Gerrits DG, van den Berg-Vos RM, Karas GB, Steyerberg EW, Flach HZ, Marquering HA, Sprengers ME, Jenniskens SF, Beenen LF, van den Berg R, Koudstaal PJ, van Zwam WH, Roos YB, van der Lugt A, van Oostenbrugge RJ, Majoie CB, Dippel DW; MR CLEAN Investigators. A randomized trial of intraarterial treatment for acute ischemic stroke. *New England Journal of Medicine*. 2015;372:11–20.
23. Goyal M, Demchuk AM, Menon BK, Eesa M, Rempel JL, Thornton J, Roy D, Jovin TG, Willinsky RA, Sapkota BL, Dowlatshahi D, Frei DF, Kamal NR, Montanera WJ, Poppe AY, Ryckborst KJ, Silver FL, Shuaib A, Tampieri D, Williams D, Bang OY, Baxter BW, Burns PA, Choe H, Heo JH, Holmstedt CA, Jankowitz B, Kelly M, Linares G, Mandzia JL, Shankar J, Sohn SI, Swartz RH, Barber PA, Coutts SB, Smith EE, Morrish WF, Weill A, Subramaniam S, Mitha AP, Wong JH, Lowerison MW, Sajobi TT, Hill MD; ESCAPE Trial Investigators. Randomized assessment of rapid endovascular treatment of ischemic stroke. *New England Journal of Medicine*. 2015;372:1019–30.
24. Campbell BC, Mitchell PJ, Kleinig TJ, Dewey HM, Churiloy L, Yassi N, Yan B, Dowling RJ, Parsons MW, Oxley TJ, Wu TY, Brooks M, Simpson MA, Miteff F, Levi CR, Krause M, Harrington TJ, Faulder KC, Steinfort BS, Priglinger M, Ang T, Scroop R, Baraber PA, McGuinness B, Wijeratne T, Phan TG, Chong W, Chandra RV, Bladin CF, Badve M, Rice H, de Villiers L, Ma H, Desmond PM, Donnon GA, Davis SM; EXTEND-IA Investigators. Endovascular therapy for ischemic stroke with perfusion-imaging selection. *New England Journal of Medicine*. 2015;372:1009–18.
25. Saver JL, Goyal M, Bonafe A, Diener HC, Levy EI, Pereira VM, Albers GW, Cognard C, Cohen DJ, Hacke W, Jansen O, Jovin TG, Mattle HP, Nogueira RG, Siddiqui AH, Yavagal DR, Baxter BW, Devlin TG, Lopes DK, Reddy VK, du Mesnil de Rochemont R, Singer OC, Jahan R; SWIFT PRIME Investigators. Stent-retriever thrombectomy after intravenous t-PA vs. t-PA alone in stroke. *New England Journal of Medicine*. 2015;372:2285–95.
26. Jovin TG, Chamorro A, Cobo E, de Miquel MA, Molina CA, Rovira A, San Román L, Serena J, Abilleira S, Ribó M, Millán M, Urra X, Cardona P, López-Cancio E, Tomasello A, Castaño C, Blasco J, Aja L, Dorado L, Quesada H, Rubiera M, Hernandez-Pérez M, Goyal M, Demchuk AM,

von Kummer R, Gallofré M, Dávalos A; REVASCAT Trial Investigators. Thrombectomy within 8 hours after symptom onset in ischemic stroke. *New England Journal of Medicine*. 2015; 372:2296–306.

27. Derdeyn CP, Chimowitz MI, Lynn MJ, Fiorella D, Turan TN, Janis LS, Montgomery J, Nizam A, Lane BF, Lutsep HL, Barnwell SL, Waters MF, Hoh BL, Hourihane JM, Levy EI, Alexandrov, AV, Harrigan MR, Chiu D, Klucznik RP, Clark JM, McDouglall CG, Johnson MD, Pride GL Jr, Lynch JR, Zaidat OO, Rumboldt Z, Cloft HJ; Stenting and Aggressive Medical management for Preventing Recurrent Stroke in Intracranial Stenosis Trial Investigators. Aggressive medical treatment with or without stenting in highrisk patients with intracranial artery stenosis (SAMMPRIS): The final results of a randomised trial. Lancet. 2014;383(9914):333-341.
28. Bartholomew Eldridge LK, Markham CM, Ruiter RAC, Fernandez ME, Kok G, Parcel GS. Chapter 9: Intervention mapping, step 6. In, *Planning Health Promotion Programs: An Intervention Mapping Approach*, 4th ed. 2016: John Willey & Sons. Published by Jossey-Bass.
29. Moore GF, Audrey S, Barker M, Bond L, Bonnell C, Hardeman W, Moore L, O'Cathain A, Tinati T, Wight D, Baird J. Process evaluation of complex interventions: Medical research council guidance. *British Medical Journal*. 2015;350:h1258 1 doi:10.1136/bmjh1258.
30. Campbell M, Fitzpatrick R, Haines A, Kinmonth AL, Sandercock P, Spiegelhalter D, Tyrer P. Framework for design and evaluation of complex interventions to improve health. *British Medical Journal*. 2000;321:694-696.
31. Carroll C, Patterson M, Wood S, Booth A, Rick J, Balain S. A conceptual framework for implementation fidelity. *Implementation Science*. 2007;2:40. Doi:10.1186/1748-5908-2-40.
32. Santacroce SJ, Maccarelli LM, Grey M. Intervention fidelity. *Nursing Research*. 2004:53(1):63-66.
33. Meinert CL, Tonascia S. Clinical Trials: Design, Conduct, and Analysis. New York: Oxford University Press; 1986.
34. Murray DM. *Design and Analysis of Group-Randomized Trials*. New York: Oxford University Press. 1998; p. 352.
35. Donner A, Klar N. Pitfalls of and controversies in cluster randomization trials. Am J Public Health. 2004 Mar;94(3):416-22.
36. Martin DC, Diehr P, Perrin EB, et al. The effect of matching on the power of randomized community intervention studies. *Stat Med.* 1993;12:329–338.
37. Jennison, C., and B. W. Turnbull: Group Sequential Methods with Applications to Clinical Trials. Chapman & Hall/CRC, 2000.
38. Montgomery D. *Introduction to Statistical Quality Control*. Hoboken, New Jersey: John Wiley & Sons, Inc. 2005.
39. Piantadosi S. Clinical Trials - A Methodologic Perspective. Second ed. Hoboken, New Jersey: Wiley; 2005.
40. Herson J. Data and Safety Monitoring Committees in Clinical Trials. Boca Raton, FL: Chapman & Hall/CRC; 2009.
41. American Thoracic Society; Infectious Diseases Society of America. Guidelines for the management of adults with hospital-acquired, ventilator-associated, and healthcare-associated pneumonia. *Am J Respir Crit Care Med*. 2005 Feb 15;171(4):388-416.
42. Chaisinanunkul N, Starkman S, Gornbein J, Hamilton S, Chatfield F, Conwit R, Saver JL. Staged use of ordinal and linear disability scales: a practical approach to granular assessment of acute stroke outcome. Front Neurol. 2023 Jun 28;14:1174686. Erratum in: Front Neurol. 2023 Dec 12;14:1331276.

SUMMARY OF RESEARCH PROTOCOL CHANGES

Research Protocol version 1.0 supported the ZODIAC study from May 10, 2018 through September 15, 2019.

Research Protocol version 2.0 was adopted following new guidelines suggesting that large vessel occlusion acute ischemic stroke patients may benefit significantly from thrombectomy when advanced imaging techniques are utilized for patient selection up to 24 hours from symptom onset. To support this recommendation, one change was made to the original protocol inclusion criteria (in red below):

Original Protocol (v. 1.0)<br><br>May 10, 2018-September 15, 2019	Revised Protocol (v. 2.0)<br><br>September 16, 2019 – November 1, 2023<br><br>(***Note:*** *Study closed to enrollment 11/01/23)*
Inclusion Criteria:<br>• Adults (>18 years)<br>• Ischemic stroke symptoms consistent with large artery occlusion<br>• Baseline standard of care non-contrast head CT (or MRI) negative for hemorrhage or mass-effect<br>• Evidence of arterial occlusion on standard of care CT angiography or MR angiography<br>• Favorable neuroimaging (Alberta Stroke Program Early Computed Tomography Score [ASPECTS] > 6 in anterior circulation stroke; not applicable in posterior circulation stroke)<br>• Anticipated treatment with mechanical thrombectomy<br>• Pre-stroke baseline modified Rankin Score (mRS) < 1<br>• Symptom onset within 6-hours of start time for catheter angiography/ planned thrombectomy procedure	*Inclusion Criteria:*<br>• Adults (>18 years)<br>• Ischemic stroke symptoms consistent with large artery occlusion<br>• Baseline standard of care non-contrast head CT (or MRI) negative for hemorrhage or mass-effect<br>• Evidence of arterial occlusion on standard of care CT angiography or MR angiography<br>• Favorable neuroimaging (Alberta Stroke Program Early Computed Tomography Score [ASPECTS] > 6 in anterior circulation stroke; not applicable in posterior circulation stroke)<br>• Anticipated treatment with mechanical thrombectomy<br>• Pre-stroke baseline modified Rankin Score (mRS) < 1<br>• Favorable imaging with ASPECTS > 6 and/or favorable CT perfusion scan (according to local site standard of care for thrombectomy patient selection) up to 24-hours from time of symptom onset
