# Supplementary material for: Optimal Head-of-Bed Positioning Before Thrombectomy in Large Vessel Occlusion Stroke: A Randomized Clinical Trial
Source: JAMA Neurol. 2025 Jun 4;82(9):905–14. doi: 10.1001/jamaneurol.2025.2253 (PMC12138796; doi:10.1001/jamaneurol.2025.2253)
Supplement: Supplement 2. — Statistical Analysis Plan. [file jamaneurol-e252253-s002.pdf]

ZODIAC STATISTICAL ANALYSIS PLAN

Protocol Title: Zero Degree Head Positioning in Hyperacute Large Artery Ischemic Stroke (ZODIAC)

Trial registration: ClinicalTrials.gov ID - NCT03728738

Sponsor:

University of Tennessee
1R01NR017850-01 (U.S. NIH Grant/Contract)

Summary of Changes:

1. Version 2.0 of the SAP (dated 11/18/2023), added the use of the Log-rank test, Kaplan-Meier curves and Cox proportional hazards modeling to analyze time-to-event variables (i.e., early neurological deterioration (primary outcome)). This modification was added after the second interim analysis. *Section 5B, Analysis Methods,* proposed that the Log-rank test and unadjusted Cox proportional-hazards modeling would be adopted to compare and Kaplan-Meier curves to visualize the rate of early neurological deterioration ($\geq$ 2 NIHSS points change from baseline) in the two groups. Similarly, using the Log-rank test and unadjusted Cox proportional-hazards modeling to compare and Kaplan-Meier curves to visualize the rate of severe early neurological deterioration ($\geq$ 4 NIHSS points change from baseline) in the two groups would be adopted. The number at risk and censored in each group would be reported in the Kaplan-Meier survival curves every 10 minutes.
2. Version 3.0 of SAP (dated 02/26/2024), added ordinal logistic regression and a utility-weighted modified Rankin Score to analyze secondary (exploratory) outcomes. These modifications were made after the second interim analysis. To generate the Utility-weighted Modified Ranking Scale Score at 90 days, the original Modified Rankin Scale (mRS) score is weighted, where each of the mRS categories (from 0 to 6) is assigned a weight (1.00, 0.91, 0.76, 0.65, 0.33, 0.00 and 0.00), and modeled as a dependent variable by unadjusted linear regression, with randomization assignment as the predictor.

Pitchaiah Mandava, MD, PhD, MSEE (Senior Statistician)

Gabriel Torrealba-Acosta, MD, MSc (Co-investigator, Deputy Statistician)

Title Page

Protocol Title: Zero Degree Head Positioning in Hyperacute Large Artery Ischemic Stroke (ZODIAC)

Trial registration: ClinicalTrials.gov ID - NCT03728738

Sponsor:

University of Tennessee
1R01NR017850-01 (U.S. NIH Grant/Contract)

SAP version: Version 1.0

Protocol version: The SAP was written based on the information in the study protocol, version 2.0, dated 09/16/2019.

Roles and Responsibilities of SAP Contributors

Pitchaiah Mandava, MD, PhD, MSEE (Senior Statistician): wrote the SAP

Gabriel Torrealba-Acosta, MD, MSc (Co-investigator, Deputy Statistician): wrote the SAP

Anne Alexandrov, PhD (Principal Investigator): reviewed the SAP

Signature Page

Protocol Title: Zero Degree Head Positioning in Hyperacute Large Artery Ischemic Stroke (ZODIAC)

**Reviewed**	**Signature**	**Date**
Pitchaiah Mandava<br>(Senior Statistician)	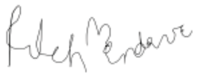	10/20/2019
Gabriel Torrealba-Acosta<br>(Co-Investigator and Deputy Statistician)	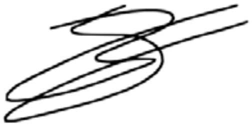	10/20/2019
Anne Alexandrov<br>(Principal Investigator)	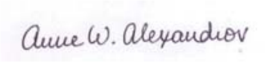	10/22/2019

Table of Contents

TABLE OF CONTENTS **5**
1. INTRODUCTION **7**
1A. BACKGROUND AND RATIONALE 7
1B. OBJECTIVES 7
2. STUDY METHODS **8**
2A. TRIAL DESIGN 8
2B. RANDOMIZATION 8
2C. SAMPLE SIZE CALCULATION 9
2D. STUDY FRAMEWORK 9
2E. STATISTICAL INTERIM ANALYSES AND STOPPING GUIDANCE 9
2F. TIMING OF FINAL ANALYSIS 10
2G. TIMING OF OUTCOME ASSESSMENTS 10
3. STATISTICAL PRINCIPLES **10**
3A. CONFIDENCE INTERVAL AND P-VALUES 10
3B. ADHERENCE AND PROTOCOL DEVIATIONS 11
3C. ANALYSIS POPULATIONS 11
4. TRIAL POPULATION **11**
4A. SCREENING, RECRUITMENT, AND WITHDRAWAL/FOLLOW-UP DATA 11
4B. ELIGIBILITY CRITERIA 13
4C. BASELINE PATIENT CHARACTERISTICS 14
5. ANALYSIS **17**
5A. OUTCOME DEFINITIONS 17
5B. ANALYSIS METHODS 18
5C. MISSING DATA 21
5D. STATISTICAL SOFTWARE 21
6. REFERENCES **22**

List of Abbreviations

AIS: Acute Ischemic Stroke
END: Early Neurologic Deterioration
HOB: head of the bed
ICP: intracranial pressure
LVO: large vessel occlusion
MT: mechanical thrombectomy
NIHSS: National Institute of Health Stroke Scale
PROBE: prospective randomized open-blinded endpoint
SICH: Symptomatic Intracranial Hemorrhage
SND: Severe neurological deterioration

1. Introduction

1a. Background and rationale

Positioning of the patient during hyperacute ischemic stroke (AIS) treatment is an important yet understudied aspect of nursing care that could impact the course of treatment and clinical outcome. Since 1968, clinical symptom worsening in AIS patients has been documented with the head of the bed (HOB) elevated to 30 degrees or higher, while clinical improvement or symptom stability has been noted with zero-degree HOB positioning.[1,2] Mechanisms for zero-degree HOB clinical improvement include favorable gravitational blood flow conditions and recruitment of collateral blood channels.[3-9] In contrast, in the case of treatment with clot-busting medications, increased blood flow may allow more medication to reach occluded arteries, facilitating clot breakdown. Despite this, there is currently a divide within the clinical community about what position is best for patients. However, it has been argued that zero-degree head positioning should be among the first steps taken to improve blood flow to the brain and prevent stroke symptoms from worsening. The investigators have shown that elevated intracranial pressure (ICP) is absent in early AIS and that pneumonia is rare using these piloted methods.[10]

However, no large clinical trial has examined the efficacy and safety of zero-degree HOB positioning within hyperacute large vessel occlusion (LVO) ischemic stroke patients with potentially viable brain tissue, leaving the acute stroke community confused as to what constitutes best practice.

1b. Objectives

The primary objective is to evaluate early neurologic deterioration (END) between the two groups according to head positioning (0° vs. 30°). END is a worsening of two or more points on the NIHSS (a stroke disability severity measure ranging from 0, indicating no disability, to 42, indicating severe disability) during the intervention period. Measurements will be taken every 10 minutes from the start of positioning until the beginning of a thrombectomy or up to 2 hours, whichever occurs first.

The secondary objectives include several assessments. First, severe neurological deterioration (SND) will be monitored, defined as a four or more point worsening on the NIHSS during the positioning intervention period, measured similarly to the primary objective. Additionally, the diagnosis of pneumonia acquired during hospitalization in patients free of pneumonia upon admission will be tracked. This diagnosis will be based on new or progressive pulmonary infiltrates on imaging and symptoms such as fever, purulent sputum, leukocytosis or leukopenia, and/or a decline in oxygen saturation. It will be assessed until hospital discharge or up to 7 days.

Further secondary objectives include monitoring participant deaths within 90 days of the onset of stroke and assessing the NIHSS score at the time of hospital discharge or on day 7, whichever comes first. Functional outcomes will be evaluated using the Modified Rankin Scale (mRS) at hospital discharge or days 7 and 90 days after stroke onset.

2. Study Methods

2a. Trial design

ZODIAC is a prospective randomized open-blinded endpoint (PROBE) clinical trial of head positioning to determine if zero-degree HOB positioning during the early phase of hyperacute LVO ischemic stroke management prevents neurological symptom worsening. Mechanical thrombectomy (MT) eligible patients (n=182) will be randomized to one of two groups (Figure 1): 1) Zero-degree HOB positioning or 2) thirty-degree HOB positioning. The hypothesis is that optimal HOB position can be determined by early neurological symptom worsening during the intervention (Aim 1) before initiation of the thrombectomy procedure, and the investigators propose that real-time deterioration may be a surrogate measure for decreased downstream perfusion, potentially impacting the viability of brain at risk for infarction. Aim 2 will confirm that zero-degree HOB positioning for AIS is safe. This nursing measure holds significant promise as an innovative adjunct method to improve AIS symptoms and ultimately reduce disability.

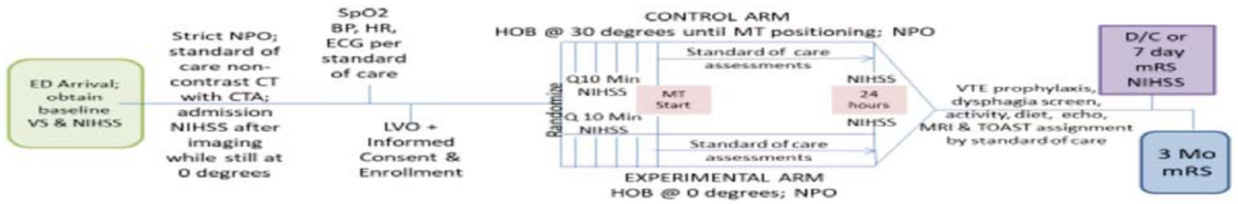

Figure 1. Summary of trial design and study protocol.

2b. Randomization

Our protocol utilizes two groups, 0°-HOB and 30°-HOB, and allows us to offer enrollment to consecutive eligible patients. To assure balance in the treatment group throughout enrollment, we will use block randomization with a block size of 4 and an allocation ratio of 1:1. This scheme has been implemented in the *Unity* (the UTHSC Center for Biomedical

Informatics (CBMI) customized biomedical clinical-research informatics application for enrollment, randomization, and data entry), computerized enrollment procedure that only shows the random assignment for a single given participant to the user. Study personnel cannot predict future assignments because of the block assignments made together with assignments at all sites.

2c. Sample Size Calculation

Our primary outcome is the percentage of patients with early neurologic deterioration (END). END is defined as two or more points worsening in the NIHSS, or required premature abortion of the assigned HOB position due to safety, or any other event that prevents participants from being evaluated (e.g., death). Not deteriorating (being stable) is defined as less than 2-point NIHSS worsening (<2) and tolerating the assigned HOB position. The primary endpoint is determined at the time of thrombectomy positioning, and we expect to see at most 5% of END participants in the 0°-HOB group, compared to 20% of END patients in the 30°-HOB group (15% lower deterioration). Our group-sequential design allows for early stopping due to futility or efficacy. It is based on a two-sample, two-sided proportion test implemented in East 6.0 (Cytel, Cambridge, MA) with type I error alpha=0.05 and 80% power. It incorporates three interim and one final look at the data, which results in a sample size of n = 182.

2d. Study Framework

This trial's primary and secondary objectives are to test the superiority of 0°-HOB positioning over 30°-HOB positioning.

2e. Statistical Interim Analyses and Stopping Guidance

Interim analyses are planned with equal spacing after cases have been enrolled/observed for the primary endpoint (Table 1). The Lan-Demets alpha and beta spending approach with O'Brien-Fleming boundaries is used for futility and efficacy interim monitoring. The absence of primary endpoint evaluation will be counted as an END event so that all randomized participants will have a determined primary endpoint available for analysis, allowing for an intent-to-treat analysis with no primary endpoint attrition. We will apply sequential testing in both patient groups. For example, the first look in the protocol is performed when 46 participants are enrolled/have their primary outcome evaluated; if the z-value associated with the test of equality of proportions in both arms is above 4.333 or below -4.333, the trial will be stopped early for efficacy (one group is superior concerning the primary outcome).
In contrast, if that z-value is in the interval (-0.007, 0.007), the trial will be stopped early for futility (groups are essentially identical in outcome). If the z-value falls within the intervals (-

4.333, -0.007) or (0.007, 4.333), the trial will continue until the following look at the data. We will utilize logistic regression to estimate odds ratios for deterioration between the respective 30 -HOB and 0 -HOB groups. Note that the absence of evaluation is counted as deterioration for the primary endpoint and that, consequently, all randomized participants will have a determined primary endpoint available for the analysis. We will use intent-to-treat principles in our primary data analysis.

Table 1. Stopping boundaries for sequential design.

Look #	Sample Size	Stopping Boundaries (Extended Protocol)			
	MT Subjects	Efficacy Z (lower, upper)		Futility Z (lower, upper)	
1	46	4.333	-4.333	0.007	-0.007
2	92	2.963	-2.963	0.374	-0.374
3	138	2.359	-2.359	1.261	-1.261
4	182	2.014	-2.014	2.014	-2.014

2f. Timing of final analysis

The final analysis will occur after all patients have reached the 3-month follow-up period.

2g. Timing of outcome assessments

The study protocol details the procedure schedule, including the expected visit dates and windows.

3. Statistical Principles

3a. Confidence Interval and P-values

All statistical testing will be 2-sided and performed using a 0.05 significance level. We will not adjust for multiple comparisons for the secondary and exploratory outcomes. Hence, p values and their corresponding confidence intervals cannot be used to infer causality. All secondary outcomes will be considered exploratory, and results will be reported with only effect size estimates and confidence intervals (CIs). All CIs presented will be 95% CI and 2-sided.

3b. Adherence and Protocol Deviations

The Unity system's data capture function with bulk export capability will enable the generation of statistical quality control charts to monitor the proportion of non-conforming patients (delivered treatment different from assigned protocol) over time. Control charts will allow us to quickly assess differences between sites and changes in these proportions as time passes so that improvements can be made swiftly. Control charts will be assembled using Unity's bulk-export function in combination with fully automatically generated reports. These charts' upper and lower control levels allow a ready assessment of whether deviations over time or between hospitals are coincidental or driven by some real difference or shift in-hospital procedures.

3c. Analysis Populations

- **Intention-to-treat (ITT):** The ITT population will include all randomized patients according to the treatment group to which they were assigned at randomization. The primary and secondary outcomes will be analyzed in the ITT population.
- **Safety analysis population:** Serious adverse events (SAE) that will be monitored closely in this trial include 1) Severe neurological deterioration (SND), 2) hospital-acquired pneumonia, and 3) death. Analyses will be descriptive and include rates for SAEs by study cohort. SND is a severe form of deterioration, and consequently, our provision for early trial stopping is also a safety measure. Because SND can be due to several factors not associated with this protocol, incidents of SND will also be reviewed by the Data Safety Monitoring Board (DSMB) and adjudicated to the intervention or control arms as indicated by their findings. Based on our pilots, we exclude cases from enrollment at high risk for pneumonia; these cases are kept NPO for thrombectomy, which will further reduce aspiration risk. Because pneumonia may be due to several different unassociated factors, pneumonia events will be reviewed by DSMB and adjudicated to the intervention as indicated. Death occurring during the active protocol phase, throughout hospitalization, or within 3 months from enrollment will be monitored, and incidents adjudicated by the DSMB. Death may be associated with several unassociated factors. Therefore, the DSMB will carefully determine the association of the study. Other AEs detected will be DSMB reviewed and reported as related or unrelated to study procedures.

4. Trial Population

4a. Screening, recruitment, and withdrawal/follow-up data

The number of screened, randomized patients, the reason for non-randomization, and the time of withdrawal and reasons for withdrawal will be reported for the overall population over the recruitment period (reported in months) as a modified version of the flow diagram (Figure 2) suggested by the CONSORT statement and guidelines.

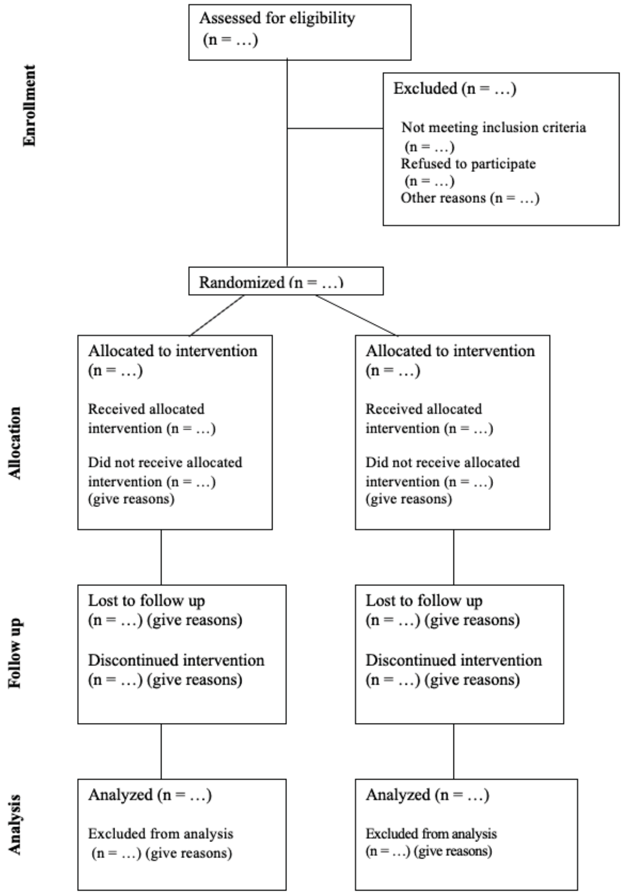

Figure 2. The suggested flow chart is per the CONSORT statement and guidelines[11].

4b. Eligibility Criteria

Table 2: Summary of eligibility criteria.

Inclusion Criteria	Exclusion Criteria
• Adults (>18 years)<br>• Ischemic stroke symptoms consistent with large artery occlusion<br>• Baseline standard of care non-contrast head CT (or MRI) negative for hemorrhage or mass-effect<br>• Evidence of arterial occlusion on standard of care CT angiography or MR angiography<br>• Favorable neuroimaging (Alberta Stroke Program Early Computed Tomography Score [ASPECTS] > 6 in anterior circulation stroke; not applicable in posterior circulation stroke)<br>• Anticipated treatment with mechanical thrombectomy<br>• Pre-stroke baseline modified Rankin Score (mRS) < 1<br>• Favorable imaging with ASPECTS > 6 and/or favorable CT perfusion scan (according to local site standard of care for thrombectomy patient selection) up to 24-hours from time of symptom onset	• Pregnancy or suspicion of pregnancy<br>• Evidence or suspicion of vomiting any time prior to consent which could predispose to aspiration pneumonia and therefore confound determination of protocol safety<br>• Anticipated palliative care referral<br>• Evidence of evolving malignant infarction on admission noncontrast CT (or MRI)<br>• Need for emergent intubation with mechanical ventilation, or non-invasive ventilatory support with either bi-level positive airway pressure (BiPAP) or continuous positive airway pressure (CPAP) due to pending or actual respiratory failure prior to or at the time of emergency department admission. (Note: Elective intubation for the thrombectomy procedure is not an exclusion criterion.)<br>• Inability to tolerate zero-degree positioning due to congestive heart failure, preexisting pneumonia, chronic obstructive pulmonary disease, or other medical condition. (Note: A diagnosis of heart failure or chronic obstructive pulmonary disease does not automatically exclude enrollment; each patient should be assessed individually for positional intolerance.)<br>• Admission chest radiograph positive for pleural effusion, pulmonary edema, pneumonia, or other pulmonary condition that may confound determination of protocol safety. (Note: An admission chest x-ray is not required, but may be obtained in patients with concerning pulmonary findings.)<br>• Abnormal breath sounds on admission assessment that may confound determination of protocol safety<br>• Lack of a telephone and/or permanent address predisposing patients to be lost to follow up<br>• Enrollment in another clinical trial that may affect our primary or secondary endpoints<br>• In the absence of a consenting legal next of kin, any medical, psychological, cognitive, social or legal condition that would interfere with informed consent and/or capacity to comply with all study requirements, including the necessary time commitment<br>• Note: Enrollment of patients receiving systemic thrombolysis more than 15 minutes prior to randomization is discouraged as this may confound ability to understand the impact of head positioning on clinical stability.

4c. Baseline patient characteristics

Baseline characteristics will be presented in a table (see Table 3), categorized by treatment groups and with the overall population values. Quantitative variables will be summarized as mean (± standard deviation) or, for non-normal distributions, as median (interquartile range). The distributions' normality will be evaluated graphically and using the Shapiro-Wilk test. A paired t-test and Wilcoxon signed-rank test will be employed to compare means and medians, respectively. Categorical variables will be shown as frequencies and percentages and will be compared with Fisher's Exact test.
Additionally, when present, the amount of missing data will be reported. P values are reported for each comparison, with a significance set at <0.05. No correction for multiple comparisons will be made.

Table 3. Summary of patients' baseline characteristics.

	0-Degrees	30-Degrees	p-Value	Overall
N				
Age, mean (SD)				
Female, n (%)				
Race, n (%)				
White				
Black				
Asian				
Hawaiian/Pacific Islander				
Native American/Alaskan				
More than one race				
Arrival or Encounter Mode, n (%)				
Ambulance (%)				
Hospital Transfer (%)				
Private Auto (%)				
Mobile Stroke Unit (%)				
Inpatient Stroke Alert (%)				

	0-Degrees	30-Degrees	p-Value	Overall
Unknown (%)				
Head Position When First Encountered by Study Team, n (%)				
0-Degrees				
30-Degrees				
Past Medical History, n (%)				
Hypertension				
TIA or Stroke				
Diabetes				
Coronary Artery Disease				
Heart Failure				
Cardiac Valvulopathy				
Peripheral Vascular Disease				
Carotid Atheroma				
Carotid Revascularization				
Thyroid Disease				
COPD				
Active Smoker				
Cancer				
Sleep Disordered Breathing				
Alcohol Abuse				
SBP, mean (SD)				
DBP, mean (SD)				
Glucose, mean (SD)				
NIHSS Baseline Time-0, median (IQR)				

	0-Degrees	30-Degrees	p-Value	Overall
ASPECTS, Median (IQR)				
Pre-Stroke Baseline Modified Rankin Score (mRS), n (%)				
mRS 0				
mRS 1				
mRS 2				
mRS 3				
Side of Occlusion, n (%)				
Right				
Left				
N/A (Vertebro-Basilar)				
Bilateral Hemispheres				
Stroke Vascular Territory, n (%)				
Middle cerebral artery				
Internal carotid artery				
Anterior cerebral artery				
Basilar				
Multi-territory				
Systemic Thrombolysis, n (%)				
Alteplase				
Tenecteplase				
Any Systemic Thrombolytic Agent				
Stroke Mechanism, n (%)				
Large Artery Atheroma				
Cardioembolism				
Stroke of Unusual Etiology				

	0-Degrees	30-Degrees	p-Value	Overall
Cryptogenic				
Unknown				

5. Analysis

5a. Outcome definitions

Primary Outcome: Early neurologic deterioration (END) on the National Institute of Health Stroke Scale occurring during the positioning intervention.

Two or more points worsening in the National Institute of Health Stroke Scale (stroke disability severity measure, ranging from 0-no disability to 42-severely disabled) score during the positioning intervention period. Measured every 10 minutes from initiation of positioning until thrombectomy commences or 2 hours - whichever comes first.

Safety Outcomes:

- **Severe Neurological Deterioration (SND) on the National Institute of Health Stroke Scale Occurring During the Positioning Intervention:** Four or more point-worsening in the National Institute of Health Stroke Scale (stroke disability severity measure, ranging from 0-no disability to 42-severely disabled) scores during the positioning intervention period. Measured every 10 minutes from initiation of positioning until thrombectomy commences or 2 hours - whichever comes first.
- **Diagnosis of Pneumonia Made During Hospitalization in a Patient Free From Pneumonia at Time of Hospital Admission:** Documented onset of a new or progressive infiltrate on pulmonary imaging along with the presence of at least two of the following[12] - Fever of 38°C/100.4°F; Purulent sputum; Leukocytosis or leukopenia; and/or, Decline in oxygen saturation. It is measured until hospital discharge or day 7 (whichever comes first).
- **Participant Deaths at discharge and within 90 days from Stroke Onset:** All-cause death occurring at discharge and from the time of randomization up until 90 days from stroke onset.
- **Post-discharge stroke:** the occurrence of a recurrent stroke within 90 days from stroke onset.

- **Symptomatic intracranial hemorrhage (SICH)** refers to an intracranial hemorrhage that worsens 4 or more points in the NIHSS within 36 hours of receiving tPA or thrombectomy.

Exploratory Outcomes:

- **Improvement at 24 hours and discharge or 7 days after stroke onset:** rates of patients that improved their total National Institute of Health Stroke Scale (stroke disability severity measure, ranging from 0-no disability to 42-severely disabled) score measured at 24 hours, discharge or 7 days after stroke onset.
- **Modified Rankin Scale Score at Hospital Discharge or Day 7:** Categorical rank on the Modified Rankin Scale score (stroke functional outcome measure ranging from 0-no functional disability, to 6-dead) measured at the time of hospital discharge or on day 7 (whichever comes first).
- **Modified Rankin Scale Score at 90 days from Stroke Onset: Categorical rank on the Modified Rankin Scale score (stroke functional outcome measure ranging from 0-no functional disability** to 6-dead) measured at 90 days from stroke onset.
- **The expanded Thrombolysis in Cerebral Infarction (eTICI) score:** a grading scale used to describe downstream arterial flow (grade 0 = no perfusion noted, grade 1 = reduction in thrombus without any resultant filling of distal arterial branches, grade 2a = reperfusion of 1-49% of the territory, grade 2b50 = reperfusion of 50-66% of the territory, grade 2b67 = reperfusion of 67-89% of the territory, grade 2c = extensive reperfusion of 90-99% of the territory, and grade 3 = complete [100%] reperfusion), will be assessed from catheter angiography taken as the first angiographic run visualizing the lesion and again at conclusion of the thrombectomy procedure. In cases where thrombectomy or other treatment is not planned, the first angiographic run eTICI score will be carried forward as the post-thrombectomy score.

5b. Analysis Methods

- **Primary Outcome:** the primary outcome will be analyzed in the intention-to-treat population. Overall rates of END and according to group will be reported as frequencies and percentages and compared between groups with Fisher's Exact test. The number needed to harm will be estimated as the inverse of the absolute risk increase (ARI) = 1/ARI.
- **Safety Outcomes:** All the safety outcome analyses will be done in the intention-to-treat population. Overall and according to group rates of SND will be reported as frequencies and percentages and compared between groups with Fisher's Exact test. The number needed to harm for SND will be estimated as the inverse of the absolute risk increase (ARI) = 1/ARI. Rates of hospital-acquired pneumonia, discharge, 90-day participant's death, post-discharge stroke, and SICH will be reported in frequencies and percentages. They will be compared between groups using Fisher's Exact test

together with odds ratios and the corresponding 95% confidence intervals and p-values from unadjusted logistic regression models that include randomization assignment as the predictor.

- **Exploratory Outcomes: The improvement rates** at 24 hours, discharge/7 days in the NIHSS will be reported as frequencies and percentages. They will be compared between groups using Fisher's Exact test together with odds ratios and the corresponding 95% confidence intervals and p-values from unadjusted logistic regression models that include randomization assignment as the predictor. Changes from baseline NIHSS for individual subjects will be plotted by means of a linear plot according to their randomization group to visualize the changes in NIHSS after randomization in each group. If needed, jitter will be introduced to the data to separate the lines—a repeat measures ANOVA will be employed to compare the repeat NIHSS measures between the two groups.

  90-day mRS will be analyzed, dichotomized as 0-2/3-6 and 0-3/4-6, and modeled as a dependent variable by unadjusted logistic regression with randomization assignment as the predictor. 90-day mRS will be plotted using a paired horizontal bar graph (aka 'Grotta bars') for visualization purposes.

No adjusting covariates were pre-specified for any of the analyzed outcomes. Except when dealing with missing data, no sensitivity or subgroup analysis was pre-specified for any of the above-described outcomes. We will not adjust for multiple comparisons for the secondary and exploratory outcomes. Hence, p values and their corresponding confidence intervals will not be used to infer causality. Outcomes will be reported in a table (see Table 4).

Table 4: Summary of primary, safety, and secondary (exploratory) outcomes.

Outcome	0-Degrees	30-Degrees	Unadjusted Hazard/Odds Ratio (95% CI)	P Value
**Primary Outcome, n (%)**				
Early neurological deterioration (≥ 2 points from baseline NIHSS)				
**Safety Outcomes, n (%)**				
Worsening of ≥ 4 points from baseline NIHSS				
Hospital Acquired Pneumonia				
Death at Discharge/Day 7				
90 day all-cause mortality				
Post-discharge stroke				
Symptomatic intracerebral hemorrhage (SICH)				
**Pre-specified Exploratory Outcomes, n (%)**				
Improvement at 24h				
Improvement at Discharge/Day 7				
90 day mRS 0-1				
90 day mRS 0-2				
90 day mRS 0-3				

5c. Missing Data

Missing data will be managed using strategies to minimize its impact on the analysis. First, efforts will be made during data collection to ensure completeness, including thorough staff training and robust data monitoring. The extent and patterns of missingness will be assessed if missing data occurs. For variables with sporadic missing values, we will assume data was missing at random and will apply multiple imputation to preserve statistical power and reduce bias. If the data are found to be missing, not at random, sensitivity analyses will be conducted to evaluate how different assumptions about the missing data mechanism may affect the results. Complete case analysis will also be considered for comparison to ensure consistency of findings across different approaches. The number and percentage of missing data will be reported, and any systematic patterns of missingness will be examined and documented.

5d. Statistical Software

All statistical analyses will be performed using R version 3.5.2 (© R Foundation for Statistical Computing, 2018). A fully annotated R Mark Down document and the trial raw dataset will be available upon request for replication and verification purposes.

6. References

1. Toole JF. Effects of change of head, limb and body position on cephalic circulation. N Engl J Med. 1968 Aug 8;279(6):307-11. doi: 10.1056/NEJM196808082790609. PMID: 5660303.
2. Caplan LR, Sergay S. Positional cerebral ischaemia. J Neurol Neurosurg Psychiatry. 1976 Apr;39(4):385-91. doi: 10.1136/jnnp.39.4.385. PMID: 932755; PMCID: PMC492290.
3. Hayashida K, Hirose Y, Kaminaga T, Ishida Y, Imakita S, Takamiya M, Yokota I, Nishimura T. Detection of postural cerebral hypoperfusion with technetium-99m-HMPAO brain SPECT in patients with cerebrovascular disease. J Nucl Med. 1993 Nov;34(11):1931-5. PMID: 8229237.
4. Ouchi Y, Nobezawa S, Yoshikawa E, Futatsubashi M, Kanno T, Okada H, Torizuka T, Nakayama T, Tanaka K. Postural effects on brain hemodynamics in unilateral cerebral artery occlusive disease: a positron emission tomography study. J Cereb Blood Flow Metab. 2001 Sep;21(9):1058-66. doi: 10.1097/00004647-200109000-00003. PMID: 11524610.
5. Wojner AW, El-Mitwalli A, Alexandrov AV. Effect of head positioning on intracranial blood flow velocities in acute ischemic stroke: a pilot study. Crit Care Nurs Q. 2002 Feb;24(4):57-66. doi: 10.1097/00002727-200202000-00007. PMID: 11833629.
6. Wojner-Alexander AW, Garami Z, Chernyshev OY, Alexandrov AV. Heads down: flat positioning improves blood flow velocity in acute ischemic stroke. Neurology. 2005 Apr 26;64(8):1354-7. doi: 10.1212/01.WNL.0000158284.41705.A5. PMID: 15851722.
7. Saqqur M, Sharma VK, Tsivgoulis G, Huy TN, Heliopoulos I, Siddiqui M, Derksen C, Khan K, Alexandrov AV. Real-time hemodynamic assessment of downstream effects of intracranial stenoses in patients with orthostatic hypoperfusion syndrome. Cerebrovasc Dis. 2010;30(4):355-61. doi: 10.1159/000319567. Epub 2010 Aug 5. PMID: 20693790; PMCID: PMC3014861.
8. Hunter AJ, Snodgrass SJ, Quain D, Parsons MW, Levi CR. HOBOE (Head-of-Bed Optimization of Elevation) Study: association of higher angle with reduced cerebral blood flow velocity in acute ischemic stroke. Phys Ther. 2011 Oct;91(10):1503-12. doi: 10.2522/ptj.20100271. Epub 2011 Aug 25. PMID: 21868612.
9. Favilla CG, Mesquita RC, Mullen M, Durduran T, Lu X, Kim MN, Minkoff DL, Kasner SE, Greenberg JH, Yodh AG, Detre JA. Optical bedside monitoring of cerebral blood flow in acute ischemic stroke patients during head-of-bed manipulation. Stroke. 2014 May;45(5):1269-74. doi: 10.1161/STROKEAHA.113.004116. Epub 2014 Mar 20. PMID: 24652308; PMCID: PMC4006296.
10. Ali LK, Weng JK, Starkman S, Saver JL, Kim D, Ovbiagele B, Buck BH, Sanossian N, Vespa P, Bang OY, Jahan R, Duckwiler GR, Viñuela F, Liebeskind DS. Heads Up! A Novel Provocative Maneuver to Guide Acute Ischemic Stroke Management. Interv Neurol. 2017 Mar;6(1-2):8-15. doi: 10.1159/000449322. Epub 2016 Sep 30. PMID: 28611828; PMCID: PMC5465685.

11. Schulz KF, Altman DG, Moher D, et al: CONSORT 2010 Statement: updated guidelines for reporting parallel group randomised trials. BMC Medicine 2010; 8(1):18
12. American Thoracic Society; Infectious Diseases Society of America. Guidelines for the management of adults with hospital-acquired, ventilator-associated, and healthcare-associated pneumonia. Am J Respir Crit Care Med. 2005 Feb 15;171(4):388-416. doi: 10.1164/rccm.200405-644ST. PMID: 15699079.

Title Page

Protocol Title: Zero Degree Head Positioning in Hyperacute Large Artery Ischemic Stroke (ZODIAC)

Trial registration: ClinicalTrials.gov ID - NCT03728738

Sponsor:

University of Tennessee
1R01NR017850-01 (U.S. NIH Grant/Contract)

SAP version: Version 2.0

Protocol version: The original SAP (v. 1.0) was written based on the information in research protocol version 2.0, dated 09/16/2019. SAP version 2.0 was amended and adopted on 11/18/2023.

SAP revision history:

3. Version 1.0 of the SAP was based on research protocol version 2.0 (date 09/16/2019).
4. Version 2.0 of the SAP added the use of Kaplan-Meier curves and Cox proportional hazards modeling to analyze time-to-event variables (i.e., early neurological deterioration (primary outcome)). This modification was added after the second interim analysis. The initial SAP proposed using Fisher's exact test to compare END rates; we considered survival analysis better suited for time-to-event variables. Date: 11/18/2023.

Roles and Responsibilities of SAP Contributors

Pitchaiah Mandava, MD, PhD, MSEE (Senior Statistician): wrote the SAP

Gabriel Torrealba-Acosta, MD, MSc (Co-investigator, Deputy Statistician): wrote the SAP

Anne Alexandrov, PhD (Principal Investigator): reviewed the SAP

Signature Page

Protocol Title: Zero Degree Head Positioning in Hyperacute Large Artery Ischemic Stroke (ZODIAC)

**Reviewed**	**Signature**	**Date**
Pitchaiah Mandava<br>(Senior Statistician)	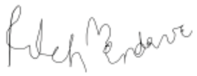	11/18/2023
Gabriel Torrealba-Acosta<br>(Co-Investigator and Deputy Statistician)	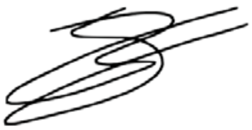	11/18/2023
Anne Alexandrov<br>(Principal Investigator)	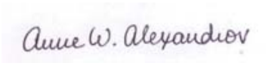	11/18/2023

Table of Contents

TABLE OF CONTENTS **5**
1. INTRODUCTION **7**
1A. BACKGROUND AND RATIONALE 7
1B. OBJECTIVES 7
2. STUDY METHODS **8**
2A. TRIAL DESIGN 8
2B. RANDOMIZATION 8
2C. SAMPLE SIZE CALCULATION 9
2D. STUDY FRAMEWORK 9
2E. STATISTICAL INTERIM ANALYSES AND STOPPING GUIDANCE 9
2F. TIMING OF FINAL ANALYSIS 10
2G. TIMING OF OUTCOME ASSESSMENTS 10
3. STATISTICAL PRINCIPLES **10**
3A. CONFIDENCE INTERVAL AND P-VALUES 10
3B. ADHERENCE AND PROTOCOL DEVIATIONS 11
3C. ANALYSIS POPULATIONS 11
4. TRIAL POPULATION **11**
4A. SCREENING, RECRUITMENT, AND WITHDRAWAL/FOLLOW-UP DATA 11
4B. ELIGIBILITY CRITERIA 13
4C. BASELINE PATIENT CHARACTERISTICS 14
5. ANALYSIS **17**
5A. OUTCOME DEFINITIONS 17
5B. ANALYSIS METHODS 18
5C. MISSING DATA 21
5D. STATISTICAL SOFTWARE 21
6. REFERENCES **22**

List of Abbreviations

AIS: Acute Ischemic Stroke
END: Early Neurologic Deterioration
HOB: head of the bed
ICP: intracranial pressure
LVO: large vessel occlusion
MT: mechanical thrombectomy
NIHSS: National Institute of Health Stroke Scale
PROBE: prospective randomized open-blinded endpoint
SICH: Symptomatic Intracranial Hemorrhage
SND: Severe neurological deterioration

1. Introduction

1a. Background and rationale

Positioning of the patient during hyperacute ischemic stroke (AIS) treatment is an important yet understudied aspect of nursing care that could impact the course of treatment and clinical outcome. Since 1968, clinical symptom worsening in AIS patients has been documented with the head of the bed (HOB) elevated to 30 degrees or higher, while clinical improvement or symptom stability has been noted with zero-degree HOB positioning.[1,2] Mechanisms for zero-degree HOB clinical improvement include favorable gravitational blood flow conditions and recruitment of collateral blood channels.[3-9] In contrast, in the case of treatment with clot-busting medications, increased blood flow may allow more medication to reach occluded arteries, facilitating clot breakdown. Despite this, there is currently a divide within the clinical community about what position is best for patients. However, it has been argued that zero-degree head positioning should be among the first steps taken to improve blood flow to the brain and prevent stroke symptoms from worsening. The investigators have shown that elevated intracranial pressure (ICP) is absent in early AIS and that pneumonia is rare using these piloted methods.[10]

However, no large clinical trial has examined the efficacy and safety of zero-degree HOB positioning within hyperacute large vessel occlusion (LVO) ischemic stroke patients with potentially viable brain tissue, leaving the acute stroke community confused as to what constitutes best practice.

1b. Objectives

The primary objective is to evaluate early neurologic deterioration (END) between the two groups according to head positioning (0° vs. 30°). END is a worsening of two or more points on the NIHSS (a stroke disability severity measure ranging from 0, indicating no disability, to 42, indicating severe disability) during the intervention period. Measurements will be taken every 10 minutes from the start of positioning until the beginning of a thrombectomy or up to 2 hours, whichever occurs first.

The secondary objectives include several assessments. First, severe neurological deterioration (SND) will be monitored, defined as a four or more point worsening on the NIHSS during the positioning intervention period, measured similarly to the primary objective. Additionally, the diagnosis of pneumonia acquired during hospitalization in patients free of pneumonia upon admission will be tracked. This diagnosis will be based on new or progressive pulmonary infiltrates on imaging and symptoms such as fever, purulent sputum, leukocytosis or leukopenia, and/or a decline in oxygen saturation. It will be assessed until hospital discharge or up to 7 days.

Further secondary objectives include monitoring participant deaths within 90 days of the onset of stroke and assessing the NIHSS score at the time of hospital discharge or on day 7, whichever comes first. Functional outcomes will be evaluated using the Modified Rankin Scale (mRS) at hospital discharge or days 7 and 90 days after stroke onset.

2. Study Methods

2a. Trial design

ZODIAC is a prospective randomized open-blinded endpoint (PROBE) clinical trial of head positioning to determine if zero-degree HOB positioning during the early phase of hyperacute LVO ischemic stroke management prevents neurological symptom worsening. Mechanical thrombectomy (MT) eligible patients (n=182) will be randomized to one of two groups (Figure 1): 1) Zero-degree HOB positioning or 2) thirty-degree HOB positioning. The hypothesis is that optimal HOB position can be determined by early neurological symptom worsening during the intervention (Aim 1) before initiation of the thrombectomy procedure, and the investigators propose that real-time deterioration may be a surrogate measure for decreased downstream perfusion, potentially impacting the viability of brain at risk for infarction. Aim 2 will confirm that zero-degree HOB positioning for AIS is safe. This nursing measure holds significant promise as an innovative adjunct method to improve AIS symptoms and ultimately reduce disability.

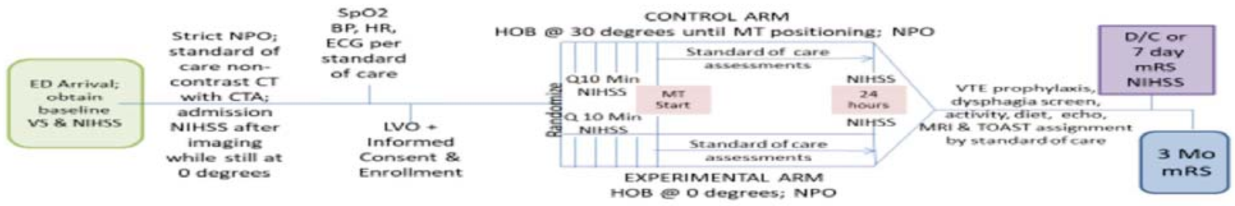

Figure 1. Summary of trial design and study protocol.

2b. Randomization

Our protocol utilizes two groups, 0°-HOB and 30°-HOB, and allows us to offer enrollment to consecutive eligible patients. To assure balance in the treatment group throughout

enrollment, we will use block randomization with a block size of 4 and an allocation ratio of 1:1. This scheme has been implemented in the *Unity* (the UTHSC Center for Biomedical Informatics (CBMI) customized biomedical clinical-research informatics application for enrollment, randomization, and data entry), computerized enrollment procedure that only shows the random assignment for a single given participant to the user. Study personnel cannot predict future assignments because of the block assignments made together with assignments at all sites.

2c. Sample Size Calculation

Our primary outcome is the percentage of patients with early neurologic deterioration (END). END is defined as two or more points worsening in the NIHSS, or required premature abortion of the assigned HOB position due to safety, or any other event that prevents participants from being evaluated (e.g., death). Not deteriorating (being stable) is defined as less than 2-point NIHSS worsening (<2) and tolerating the assigned HOB position. The primary endpoint is determined at the time of thrombectomy positioning, and we expect to see at most 5% of END participants in the 0°-HOB group, compared to 20% of END patients in the 30°-HOB group (15% lower deterioration). Our group-sequential design allows for early stopping due to futility or efficacy. It is based on a two-sample, two-sided proportion test implemented in East 6.0 (Cytel, Cambridge, MA) with type I error alpha=0.05 and 80% power. It incorporates three interim and one final look at the data, which results in a sample size of n = 182.

2d. Study Framework

This trial's primary and secondary objectives are to test the superiority of 0°-HOB positioning over 30°-HOB positioning.

2e. Statistical Interim Analyses and Stopping Guidance

Interim analyses are planned with equal spacing after cases have been enrolled/observed for the primary endpoint (Table 1). The Lan-Demets alpha and beta spending approach with O'Brien-Fleming boundaries is used for futility and efficacy interim monitoring. The absence of primary endpoint evaluation will be counted as an END event so that all randomized participants will have a determined primary endpoint available for analysis, allowing for an intent-to-treat analysis with no primary endpoint attrition. We will apply sequential testing in both patient groups. For example, the first look in the protocol is performed when 46 participants are enrolled/have their primary outcome evaluated; if the z-value associated with the test of equality of proportions in both arms is above 4.333 or below -4.333, the trial will be stopped early for efficacy (one group is superior concerning the primary outcome).

In contrast, if that z-value is in the interval (-0.007, 0.007), the trial will be stopped early for futility (groups are essentially identical in outcome). If the z-value falls within the intervals (-4.333, -0.007) or (0.007, 4.333), the trial will continue until the following look at the data. We will utilize logistic regression to estimate odds ratios for deterioration between the respective 30 -HOB and 0 -HOB groups. Note that the absence of evaluation is counted as deterioration for the primary endpoint and that, consequently, all randomized participants will have a determined primary endpoint available for the analysis. We will use intent-to-treat principles in our primary data analysis.

Table 1. Stopping boundaries for sequential design.

Look #	Sample Size	Stopping Boundaries (Extended Protocol)			
	MT Subjects	Efficacy Z (lower, upper)		Futility Z (lower, upper)	
1	46	4.333	-4.333	0.007	-0.007
2	92	2.963	-2.963	0.374	-0.374
3	138	2.359	-2.359	1.261	-1.261
4	182	2.014	-2.014	2.014	-2.014

2f. Timing of final analysis

The final analysis will occur after all patients have reached the 3-month follow-up period.

2g. Timing of outcome assessments

The study protocol details the procedure schedule, including the expected visit dates and windows.

3. Statistical Principles

3a. Confidence Interval and P-values

All statistical testing will be 2-sided and performed using a 0.05 significance level. We will not adjust for multiple comparisons for the secondary and exploratory outcomes. Hence, p values and their corresponding confidence intervals cannot be used to infer causality. All secondary outcomes will be considered exploratory, and results will be reported with only effect size estimates and confidence intervals (CIs). All CIs presented will be 95% CI and 2-sided.

3b. Adherence and Protocol Deviations

The Unity system's data capture function with bulk export capability will enable the generation of statistical quality control charts to monitor the proportion of non-conforming patients (delivered treatment different from assigned protocol) over time. Control charts will allow us to quickly assess differences between sites and changes in these proportions as time passes so that improvements can be made swiftly. Control charts will be assembled using Unity's bulk-export function in combination with fully automatically generated reports. These charts' upper and lower control levels allow a ready assessment of whether deviations over time or between hospitals are coincidental or driven by some real difference or shift in-hospital procedures.

3c. Analysis Populations

- **Intention-to-treat (ITT):** The ITT population will include all randomized patients according to the treatment group to which they were assigned at randomization. The primary and secondary outcomes will be analyzed in the ITT population.
- **Safety analysis population:** Serious adverse events (SAE) that will be monitored closely in this trial include 1) Severe neurological deterioration (SND), 2) hospital-acquired pneumonia, and 3) death. Analyses will be descriptive and include rates for SAEs by study cohort. SND is a severe form of deterioration, and consequently, our provision for early trial stopping is also a safety measure. Because SND can be due to several factors not associated with this protocol, incidents of SND will also be reviewed by the Data Safety Monitoring Board (DSMB) and adjudicated to the intervention or control arms as indicated by their findings. Based on our pilots, we exclude cases from enrollment at high risk for pneumonia; these cases are kept NPO for thrombectomy, which will further reduce aspiration risk. Because pneumonia may be due to several different unassociated factors, pneumonia events will be reviewed by DSMB and adjudicated to the intervention as indicated. Death occurring during the active protocol phase, throughout hospitalization, or within 3 months from enrollment will be monitored, and incidents adjudicated by the DSMB. Death may be associated with several unassociated factors. Therefore, the DSMB will carefully determine the association of the study. Other AEs detected will be DSMB reviewed and reported as related or unrelated to study procedures.

4. Trial Population

4a. Screening, recruitment, and withdrawal/follow-up data

The number of screened, randomized patients, the reason for non-randomization, and the time of withdrawal and reasons for withdrawal will be reported for the overall population over the recruitment period (reported in months) as a modified version of the flow diagram (Figure 2) suggested by the CONSORT statement and guidelines.

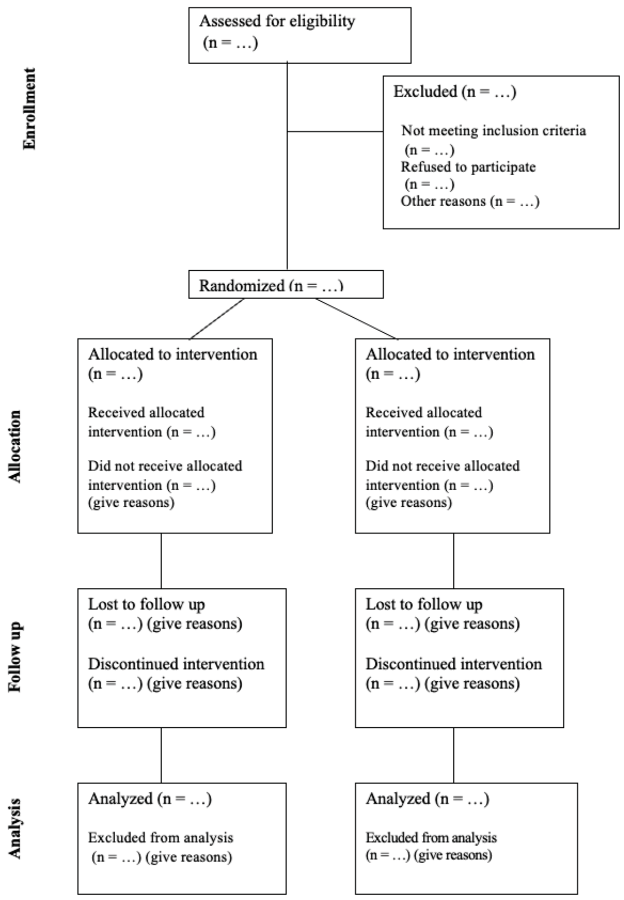

Figure 2. The suggested flow chart is per the CONSORT statement and guidelines[11].

4b. Eligibility Criteria

Table 2: Summary of eligibility criteria.

Inclusion Criteria	Exclusion Criteria
• Adults (>18 years)<br>• Ischemic stroke symptoms consistent with large artery occlusion<br>• Baseline standard of care non-contrast head CT (or MRI) negative for hemorrhage or mass-effect<br>• Evidence of arterial occlusion on standard of care CT angiography or MR angiography<br>• Favorable neuroimaging (Alberta Stroke Program Early Computed Tomography Score<br>• [ASPECTS] > 6 in anterior circulation stroke; not applicable in posterior circulation stroke)<br>• Anticipated treatment with mechanical thrombectomy<br>• Pre-stroke baseline modified Rankin Score (mRS) < 1<br>• Favorable imaging with ASPECTS > 6 and/or favorable CT perfusion scan (according to local site standard of care for thrombectomy patient selection) up to 24-hours from time of symptom onset	• Pregnancy or suspicion of pregnancy<br>• Evidence or suspicion of vomiting any time prior to consent which could predispose to aspiration pneumonia and therefore confound determination of protocol safety<br>• Anticipated palliative care referral<br>• Evidence of evolving malignant infarction on admission noncontrast CT (or MRI)<br>• Need for emergent intubation with mechanical ventilation, or non-invasive ventilatory support with either bi-level positive airway pressure (BiPAP) or continuous positive airway pressure (CPAP) due to pending or actual respiratory failure prior to or at the time of emergency department admission. (Note: Elective intubation for the thrombectomy procedure is not an exclusion criterion.)<br>• Inability to tolerate zero-degree positioning due to congestive heart failure, preexisting pneumonia, chronic obstructive pulmonary disease, or other medical condition. (Note: A diagnosis of heart failure or chronic obstructive pulmonary disease does not automatically exclude enrollment; each patient should be assessed individually for positional intolerance.)<br>• Admission chest radiograph positive for pleural effusion, pulmonary edema, pneumonia, or other pulmonary condition that may confound determination of protocol safety. (Note: An admission chest x-ray is not required, but may be obtained in patients with concerning pulmonary findings.)<br>• Abnormal breath sounds on admission assessment that may confound determination of protocol safety<br>• Lack of a telephone and/or permanent address predisposing patients to be lost to follow up<br>• Enrollment in another clinical trial that may affect our primary or secondary endpoints<br>• In the absence of a consenting legal next of kin, any medical, psychological, cognitive, social or legal condition that would interfere with informed consent and/or capacity to comply with all study requirements, including the necessary time commitment<br>• Note: Enrollment of patients receiving systemic thrombolysis more than 15 minutes prior to randomization is discouraged as this may confound ability to understand the impact of head positioning on clinical stability.

4c. Baseline patient characteristics

Baseline characteristics will be presented in a table (see Table 3), categorized by treatment groups and with the overall population values. Quantitative variables will be summarized as mean (± standard deviation) or, for non-normal distributions, as median (interquartile range). The distributions' normality will be evaluated graphically and using the Shapiro-Wilk test. A paired t-test and Wilcoxon signed-rank test will be employed to compare means and medians, respectively. Categorical variables will be shown as frequencies and percentages and will be compared with Fisher's Exact test.

Additionally, when present, the amount of missing data will be reported. P values are reported for each comparison, with a significance set at <0.05. No correction for multiple comparisons will be made.

Table 3. Summary of patients' baseline characteristics.

	0-Degrees	30-Degrees	p-Value	Overall
N				
Age, mean (SD)				
Female, n (%)				
Race, n (%)				
White				
Black				
Asian				
Hawaiian/Pacific Islander				
Native American/Alaskan				
More than one race				
Arrival or Encounter Mode, n (%)				
Ambulance (%)				
Hospital Transfer (%)				
Private Auto (%)				
Mobile Stroke Unit (%)				
Inpatient Stroke Alert (%)				

	0-Degrees	30-Degrees	p-Value	Overall
Unknown (%)				
Head Position When First Encountered by Study Team, n (%)				
0-Degrees				
30-Degrees				
Past Medical History, n (%)				
Hypertension				
TIA or Stroke				
Diabetes				
Coronary Artery Disease				
Heart Failure				
Cardiac Valvulopathy				
Peripheral Vascular Disease				
Carotid Atheroma				
Carotid Revascularization				
Thyroid Disease				
COPD				
Active Smoker				
Cancer				
Sleep Disordered Breathing				
Alcohol Abuse				
SBP, mean (SD)				
DBP, mean (SD)				
Glucose, mean (SD)				
NIHSS Baseline Time-0, median (IQR)				

	0-Degrees	30-Degrees	p-Value	Overall
ASPECTS, Median (IQR)				
Pre-Stroke Baseline Modified Rankin Score (mRS), n (%)				
mRS 0				
mRS 1				
mRS 2				
mRS 3				
Side of Occlusion, n (%)				
Right				
Left				
N/A (Vertebro-Basilar)				
Bilateral Hemispheres				
Stroke Vascular Territory, n (%)				
Middle cerebral artery				
Internal carotid artery				
Anterior cerebral artery				
Basilar				
Multi-territory				
Systemic Thrombolysis, n (%)				
Alteplase				
Tenecteplase				
Any Systemic Thrombolytic Agent				
Stroke Mechanism, n (%)				
Large Artery Atheroma				
Cardioembolism				
Stroke of Unusual Etiology				

	0-Degrees	30-Degrees	p-Value	Overall
Cryptogenic				
Unknown				
Positive COVID Diagnosis, n (%)				

5. Analysis

5a. Outcome definitions

Primary Outcome: Early neurologic deterioration (END) on the National Institute of Health Stroke Scale occurring during the positioning intervention.

Two or more points worsening in the National Institute of Health Stroke Scale (stroke disability severity measure, ranging from 0-no disability to 42-severely disabled) score during the positioning intervention period. Measured every 10 minutes from initiation of positioning until thrombectomy commences or 2 hours - whichever comes first.

Safety Outcomes:

- **Severe Neurological Deterioration (SND) on the National Institute of Health Stroke Scale Occurring During the Positioning Intervention:** Four or more point-worsening in the National Institute of Health Stroke Scale (stroke disability severity measure, ranging from 0-no disability to 42-severely disabled) scores during the positioning intervention period. Measured every 10 minutes from initiation of positioning until thrombectomy commences or 2 hours - whichever comes first.
- **Diagnosis of Pneumonia Made During Hospitalization in a Patient Free From Pneumonia at Time of Hospital Admission:** Documented onset of a new or progressive infiltrate on pulmonary imaging along with the presence of at least two of the following[12] - Fever of 38°C/100.4°F; Purulent sputum; Leukocytosis or leukopenia; and/or, Decline in oxygen saturation. It is measured until hospital discharge or day 7 (whichever comes first).
- **Participant Deaths at discharge and within 90 days from Stroke Onset:** All-cause death occurring at discharge and from the time of randomization up until 90 days from stroke onset.
- **Post-discharge stroke:** the occurrence of a recurrent stroke within 90 days from stroke onset.

- **Symptomatic intracranial hemorrhage (SICH)** refers to an intracranial hemorrhage that worsens 4 or more points in the NIHSS within 36 hours of receiving tPA or thrombectomy.

Exploratory Outcomes:

- **Improvement at 24 hours and discharge or 7 days after stroke onset:** rates of patients that improved their total National Institute of Health Stroke Scale (stroke disability severity measure, ranging from 0-no disability to 42-severely disabled) score measured at 24 hours, discharge or 7 days after stroke onset.
- **Modified Rankin Scale Score at Hospital Discharge or Day 7:** Categorical rank on the Modified Rankin Scale score (stroke functional outcome measure ranging from 0-no functional disability, to 6-dead) measured at the time of hospital discharge or on day 7 (whichever comes first).
- **Modified Rankin Scale Score at 90 days from Stroke Onset: Categorical rank on the Modified Rankin Scale score (stroke functional outcome measure ranging from 0-no functional disability** to 6-dead) measured at 90 days from stroke onset.
- **The expanded Thrombolysis in Cerebral Infarction (eTICI) score:** a grading scale used to describe downstream arterial flow (grade 0 = no perfusion noted, grade 1 = reduction in thrombus without any resultant filling of distal arterial branches, grade 2a = reperfusion of 1-49% of the territory, grade 2b50 = reperfusion of 50-66% of the territory, grade 2b67 = reperfusion of 67-89% of the territory, grade 2c = extensive reperfusion of 90-99% of the territory, and grade 3 = complete [100%] reperfusion), will be assessed from catheter angiography taken as the first angiographic run visualizing the lesion and again at conclusion of the thrombectomy procedure. In cases where thrombectomy or other treatment is not planned, the first angiographic run eTICI score will be carried forward as the post-thrombectomy score.

5b. Analysis Methods

- **Primary Outcome:** the primary outcome will be analyzed in the intention-to-treat population. We will use the Log-rank test, unadjusted Cox proportional-hazards modeling to compare and Kaplan-Meier curves to visualize the rate of early neurological deterioration ($\geq$ 2 NIHSS points change from baseline) in the two groups. The number at risk and censored in each group will be reported in the Kaplan-Meier survival curves every 10 minutes. The number needed to harm will be estimated as the inverse of the absolute risk increase (ARI) = 1/ARI.
- **Safety Outcomes:** All the safety outcome analyses will be done in the intention-to-treat population. We will the use Log-rank test, unadjusted Cox proportional-hazards modeling to compare and Kaplan-Meier curves to visualize the rate of severe early neurological deterioration ($\geq$ 4 NIHSS points change from baseline) in the two groups. The number at risk and censored in each group will be reported in the Kaplan-Meier

survival curves every 10 minutes. The number needed to harm for SND will be estimated as the inverse of the absolute risk increase (ARI) = 1/ARI. Rates of hospital-acquired pneumonia, discharge, 90-day participant's death, post-discharge stroke, and SICH will be reported in frequencies and percentages. They will be compared between groups using Fisher's Exact test together with odds ratios and the corresponding 95% confidence intervals and p-values from unadjusted logistic regression models that include randomization assignment as the predictor.

- **Exploratory Outcomes: The improvement rates** at 24 hours, discharge/7 days in the NIHSS will be reported as frequencies and percentages. They will be compared between groups using Fisher's Exact test together with odds ratios and the corresponding 95% confidence intervals and p-values from unadjusted logistic regression models that include randomization assignment as the predictor. Changes from baseline NIHSS for individual subjects will be plotted by means of a linear plot according to their randomization group to visualize the changes in NIHSS after randomization in each group. If needed, jitter will be introduced to the data to separate the lines—a repeat measures ANOVA will be employed to compare the repeat NIHSS measures between the two groups.

  90-day mRS will be analyzed, dichotomized as 0-2/3-6 and 0-3/4-6, and modeled as a dependent variable by unadjusted logistic regression with randomization assignment as the predictor. 90-day mRS will be plotted using a paired horizontal bar graph (aka 'Grotta bars') for visualization purposes.

No adjusting covariates were pre-specified for any of the analyzed outcomes. Except when dealing with missing data, no sensitivity or subgroup analysis was pre-specified for any of the above-described outcomes. We will not adjust for multiple comparisons for the secondary and exploratory outcomes. Hence, p values and their corresponding confidence intervals will not be used to infer causality. Outcomes will be reported in a table (see Table 4).

Table 4: Summary of primary, safety, and secondary (exploratory) outcomes.

Outcome	0-Degrees	30-Degrees	Unadjusted Hazard/Odds Ratio (95% CI)	P Value
**Primary Outcome, n (%)**				
Early neurological deterioration (≥ 2 points from baseline NIHSS)				
**Safety Outcomes, n (%)**				
Worsening of ≥ 4 points from baseline NIHSS				
Hospital Acquired Pneumonia				
Death at Discharge/Day 7				
90 day all-cause mortality				
Post-discharge stroke				
Symptomatic intracerebral hemorrhage (SICH)				
**Pre-specified Exploratory Outcomes, n (%)**				
Improvement at 24h				
Improvement at Discharge/Day 7				
90 day mRS 0-1				
90 day mRS 0-2				
90 day mRS 0-3				

5c. Missing Data

Missing data will be managed using strategies to minimize its impact on the analysis. First, efforts will be made during data collection to ensure completeness, including thorough staff training and robust data monitoring. The extent and patterns of missingness will be assessed if missing data occurs. For variables with sporadic missing values, we will assume data was missing at random and will apply multiple imputation to preserve statistical power and reduce bias. If the data are found to be missing, not at random, sensitivity analyses will be conducted to evaluate how different assumptions about the missing data mechanism may affect the results. Complete case analysis will also be considered for comparison to ensure consistency of findings across different approaches. The number and percentage of missing data will be reported, and any systematic patterns of missingness will be examined and documented.

5d. Statistical Software

All statistical analyses will be performed using R version 4.3.2 (© R Foundation for Statistical Computing, 2023). A fully annotated R Mark Down document and the trial raw dataset will be available upon request for replication and verification purposes.

6. References

13. Toole JF. Effects of change of head, limb and body position on cephalic circulation. N Engl J Med. 1968 Aug 8;279(6):307-11. doi: 10.1056/NEJM196808082790609. PMID: 5660303.
14. Caplan LR, Sergay S. Positional cerebral ischaemia. J Neurol Neurosurg Psychiatry. 1976 Apr;39(4):385-91. doi: 10.1136/jnnp.39.4.385. PMID: 932755; PMCID: PMC492290.
15. Hayashida K, Hirose Y, Kaminaga T, Ishida Y, Imakita S, Takamiya M, Yokota I, Nishimura T. Detection of postural cerebral hypoperfusion with technetium-99m-HMPAO brain SPECT in patients with cerebrovascular disease. J Nucl Med. 1993 Nov;34(11):1931-5. PMID: 8229237.
16. Ouchi Y, Nobezawa S, Yoshikawa E, Futatsubashi M, Kanno T, Okada H, Torizuka T, Nakayama T, Tanaka K. Postural effects on brain hemodynamics in unilateral cerebral artery occlusive disease: a positron emission tomography study. J Cereb Blood Flow Metab. 2001 Sep;21(9):1058-66. doi: 10.1097/00004647-200109000-00003. PMID: 11524610.
17. Wojner AW, El-Mitwalli A, Alexandrov AV. Effect of head positioning on intracranial blood flow velocities in acute ischemic stroke: a pilot study. Crit Care Nurs Q. 2002 Feb;24(4):57-66. doi: 10.1097/00002727-200202000-00007. PMID: 11833629.
18. Wojner-Alexander AW, Garami Z, Chernyshev OY, Alexandrov AV. Heads down: flat positioning improves blood flow velocity in acute ischemic stroke. Neurology. 2005 Apr 26;64(8):1354-7. doi: 10.1212/01.WNL.0000158284.41705.A5. PMID: 15851722.
19. Saqqur M, Sharma VK, Tsivgoulis G, Huy TN, Heliopoulos I, Siddiqui M, Derksen C, Khan K, Alexandrov AV. Real-time hemodynamic assessment of downstream effects of intracranial stenoses in patients with orthostatic hypoperfusion syndrome. Cerebrovasc Dis. 2010;30(4):355-61. doi: 10.1159/000319567. Epub 2010 Aug 5. PMID: 20693790; PMCID: PMC3014861.
20. Hunter AJ, Snodgrass SJ, Quain D, Parsons MW, Levi CR. HOBOE (Head-of-Bed Optimization of Elevation) Study: association of higher angle with reduced cerebral blood flow velocity in acute ischemic stroke. Phys Ther. 2011 Oct;91(10):1503-12. doi: 10.2522/ptj.20100271. Epub 2011 Aug 25. PMID: 21868612.
21. Favilla CG, Mesquita RC, Mullen M, Durduran T, Lu X, Kim MN, Minkoff DL, Kasner SE, Greenberg JH, Yodh AG, Detre JA. Optical bedside monitoring of cerebral blood flow in acute ischemic stroke patients during head-of-bed manipulation. Stroke. 2014 May;45(5):1269-74. doi: 10.1161/STROKEAHA.113.004116. Epub 2014 Mar 20. PMID: 24652308; PMCID: PMC4006296.
22. Ali LK, Weng JK, Starkman S, Saver JL, Kim D, Ovbiagele B, Buck BH, Sanossian N, Vespa P, Bang OY, Jahan R, Duckwiler GR, Viñuela F, Liebeskind DS. Heads Up! A Novel Provocative Maneuver to Guide Acute Ischemic Stroke Management. Interv Neurol. 2017 Mar;6(1-2):8-15. doi: 10.1159/000449322. Epub 2016 Sep 30. PMID: 28611828; PMCID: PMC5465685.

23. Schulz KF, Altman DG, Moher D, et al: CONSORT 2010 Statement: updated guidelines for reporting parallel group randomised trials. BMC Medicine 2010; 8(1):18
24. American Thoracic Society; Infectious Diseases Society of America. Guidelines for the management of adults with hospital-acquired, ventilator-associated, and healthcare-associated pneumonia. Am J Respir Crit Care Med. 2005 Feb 15;171(4):388-416. doi: 10.1164/rccm.200405-644ST. PMID: 15699079.

Title Page

Protocol Title: Zero Degree Head Positioning in Hyperacute Large Artery Ischemic Stroke (ZODIAC)

Trial registration: ClinicalTrials.gov ID - NCT03728738

Sponsor:

University of Tennessee
1R01NR017850-01 (U.S. NIH Grant/Contract)

SAP version: Version 3.0. Date: 02/26/2024.

Protocol version: The original SAP was written based on the information in the study protocol, version 2.0, dated 09/16/2019. SAP version 2.0 was amended and adopted on 11/18/2023. SAP version 3.0 was amended and adopted on 2/26/2024.

SAP revision history:

5. Version 1.0 of the SAP was based on research protocol 2.0 (date 09/16/2019).
6. Version 2.0 of the SAP added the use of Kaplan-Meier curves, the Log-rank test, and Cox proportional hazards modeling to analyze time-to-event variables (i.e., early neurological deterioration (primary outcome)). This modification was added after the second interim analysis. The initial SAP proposed using Fisher's exact test to compare END rates; we considered survival analysis better suited for time-to-event variables. Date: 11/18/2023.
7. Version 3.0 of SAP added ordinal logistic regression and a utility-weighted modified Rankin Score to analyze secondary (exploratory) outcomes (dated 02/26/2024).

Roles and Responsibilities of SAP Contributors

Pitchaiah Mandava, MD, PhD, MSEE (Senior Statistician): wrote the SAP

Gabriel Torrealba-Acosta, MD, MSc (Co-investigator, Deputy Statistician): wrote the SAP

Anne Alexandrov, PhD (Principal Investigator): reviewed the SAP

Signature Page

Protocol Title: Zero Degree Head Positioning in Hyperacute Large Artery Ischemic Stroke (ZODIAC)

Reviewed	Signature	Date
Pitchaiah Mandava<br>(Senior Statistician)	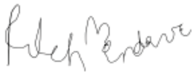	02/28/2024
Gabriel Torrealba-Acosta<br>(Co-investigator and Deputy Statistician)	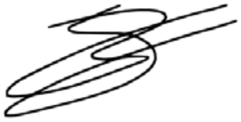	02/28/2024
Anne Alexandrov<br>(Principal Investigator)	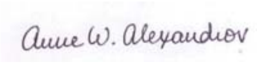	02/28/2024

Table of Contents

TABLE OF CONTENTS **47**
1. INTRODUCTION **49**
1A. BACKGROUND AND RATIONALE 49
1B. OBJECTIVES 49
2. STUDY METHODS **50**
2A. TRIAL DESIGN 50
2B. RANDOMIZATION 50
2C. SAMPLE SIZE CALCULATION 51
2D. STUDY FRAMEWORK 51
2E. STATISTICAL INTERIM ANALYSES AND STOPPING GUIDANCE 51
2F. TIMING OF FINAL ANALYSIS 52
2G. TIMING OF OUTCOME ASSESSMENTS 52
3. STATISTICAL PRINCIPLES **52**
3A. CONFIDENCE INTERVAL AND P-VALUES 52
3B. ADHERENCE AND PROTOCOL DEVIATIONS 53
3C. ANALYSIS POPULATIONS 53
4. TRIAL POPULATION **54**
4A. SCREENING, RECRUITMENT, AND WITHDRAWAL/FOLLOW-UP DATA 54
4B. ELIGIBILITY CRITERIA 54
4C. BASELINE PATIENT CHARACTERISTICS 56
5. ANALYSIS **59**
5A. OUTCOME DEFINITIONS 59
5B. ANALYSIS METHODS 60
5C. MISSING DATA 63
5D. STATISTICAL SOFTWARE 63
6. REFERENCES **64**

List of Abbreviations

AIS: Acute Ischemic Stroke
END: Early Neurologic Deterioration
HOB: head of the bed
ICP: intracranial pressure
LVO: large vessel occlusion
MT: mechanical thrombectomy
NIHSS: National Institute of Health Stroke Scale
PROBE: prospective randomized open-blinded endpoint
SICH: Symptomatic Intracranial Hemorrhage
SND: Severe neurological deterioration

1. Introduction

1a. Background and rationale

Positioning of the patient during hyperacute ischemic stroke (AIS) treatment is an important yet understudied aspect of nursing care that could impact the course of treatment and clinical outcome. Since 1968, clinical symptom worsening in AIS patients has been documented with the head of the bed (HOB) elevated to 30 degrees or higher, while clinical improvement or symptom stability has been noted with zero-degree HOB positioning.[1,2] Mechanisms for zero-degree HOB clinical improvement include favorable gravitational blood flow conditions and recruitment of collateral blood channels.[3-9] In contrast, in the case of treatment with clot-busting medications, increased blood flow may allow more medication to reach occluded arteries, facilitating clot breakdown. Despite this, there is currently a divide within the clinical community about what position is best for patients. However, it has been argued that zero-degree head positioning should be among the first steps taken to improve blood flow to the brain and prevent stroke symptoms from worsening. The investigators have shown that elevated intracranial pressure (ICP) is absent in early AIS and that pneumonia is rare using these piloted methods.[10-11]

However, no large clinical trial has examined the efficacy and safety of zero-degree HOB positioning within hyperacute large vessel occlusion (LVO) ischemic stroke patients with potentially viable brain tissue, leaving the acute stroke community confused as to what constitutes best practice.

1b. Objectives

The primary objective is to evaluate early neurologic deterioration (END) between the two groups according to head positioning (0° vs. 30°). END is a worsening of two or more points on the NIHSS (a stroke disability severity measure ranging from 0, indicating no disability, to 42, indicating severe disability) during the intervention period. Measurements will be taken every 10 minutes from the start of positioning until the beginning of a thrombectomy or up to 2 hours, whichever occurs first.

The secondary objectives include several assessments. First, severe neurological deterioration (SND) will be monitored, defined as a four or more point worsening on the NIHSS during the positioning intervention period, measured similarly to the primary objective. Additionally, the diagnosis of pneumonia acquired during hospitalization in patients free of pneumonia upon admission will be tracked. This diagnosis will be based on new or progressive pulmonary infiltrates on imaging and symptoms such as fever, purulent sputum, leukocytosis or leukopenia, and/or a decline in oxygen saturation. It will be assessed until hospital discharge or up to 7 days.

Further secondary objectives include monitoring participant deaths within 90 days of the onset of stroke and assessing the NIHSS score at the time of hospital discharge or on day 7, whichever comes first. Functional outcomes will be evaluated using the Modified Rankin Scale (mRS) at hospital discharge or days 7 and 90 days after stroke onset. The mRS scores will also be utility-weighted, with assigned weights for each category ranging from 0 (no functional disability) to 6 (dead) and compared between intervention groups.

2. Study Methods

2a. Trial design

ZODIAC is a prospective randomized open-blinded endpoint (PROBE) clinical trial of head positioning to determine if zero-degree HOB positioning during the early phase of hyperacute LVO ischemic stroke management prevents neurological symptom worsening. Mechanical thrombectomy (MT) eligible patients (n=182) will be randomized to one of two groups (Figure 1): 1) Zero-degree HOB positioning or 2) thirty-degree HOB positioning. The hypothesis is that optimal HOB position can be determined by early neurological symptom worsening during the intervention (Aim 1) before initiation of the thrombectomy procedure, and the investigators propose that real-time deterioration may be a surrogate measure for decreased downstream perfusion, potentially impacting the viability of brain at risk for infarction. Aim 2 will confirm that zero-degree HOB positioning for AIS is safe. This nursing measure holds significant promise as an innovative adjunct method to improve AIS symptoms and ultimately reduce disability.

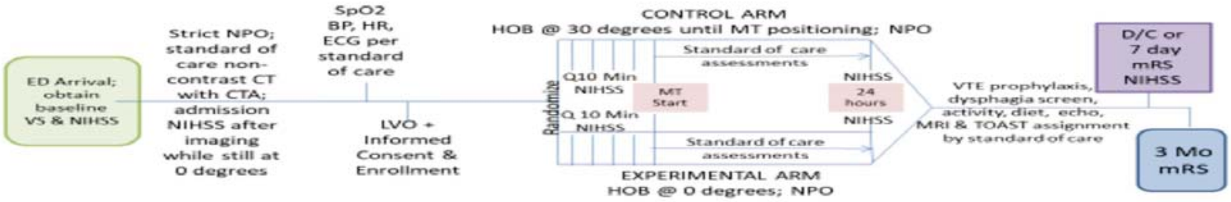

Figure 1. Summary of trial design and study protocol.

2b. Randomization

Our protocol utilizes two groups, 0°-HOB and 30°-HOB, and allows us to offer enrollment to consecutive eligible patients. To assure balance in the treatment group throughout enrollment, we will use block randomization with a block size of 4 and an allocation ratio of 1:1. This scheme has been implemented in the *Unity* (the UTHSC Center for Biomedical Informatics (CBMI) customized biomedical clinical-research informatics application for enrollment, randomization, and data entry*)*, computerized enrollment procedure that only shows the random assignment for a single given participant to the user. Study personnel cannot predict future assignments because of the block assignments made together with assignments at all sites.

2c. Sample Size Calculation

Our primary outcome is the percentage of patients with early neurologic deterioration (END). END is defined as two or more points worsening in the NIHSS, or required premature abortion of the assigned HOB position due to safety, or any other event that prevents participants from being evaluated (e.g., death). Not deteriorating (being stable) is defined as less than 2-point NIHSS worsening (<2) and tolerating the assigned HOB position. The primary endpoint is determined at the time of thrombectomy positioning, and we expect to see at most 5% of END participants in the 0°-HOB group, compared to 20% of END patients in the 30°-HOB group (15% lower deterioration). Our group-sequential design allows for early stopping due to futility or efficacy. It is based on a two-sample, two-sided proportion test implemented in East 6.0 (Cytel, Cambridge, MA) with type I error alpha=0.05 and 80% power. It incorporates three interim and one final look at the data, which results in a sample size of n = 182.

2d. Study Framework

This trial's primary and secondary objectives are to test the superiority of 0°-HOB positioning over 30°-HOB positioning.

2e. Statistical Interim Analyses and Stopping Guidance

Interim analyses are planned with equal spacing after cases have been enrolled/observed for the primary endpoint (Table 1). The Lan-Demets alpha and beta spending approach with O’Brien-Fleming boundaries is used for futility and efficacy interim monitoring. The absence of primary endpoint evaluation will be counted as an END event so that all randomized participants will have a determined primary endpoint available for analysis, allowing for an intent-to-treat analysis with no primary endpoint attrition. We will apply sequential testing in both patient groups. For example, the first look in the protocol is performed when 46 participants are enrolled/have their primary outcome evaluated; if the z-value associated

with the test of equality of proportions in both arms is above 4.333 or below -4.333, the trial will be stopped early for efficacy (one group is superior concerning the primary outcome). In contrast, if that z-value is in the interval (-0.007, 0.007), the trial will be stopped early for futility (groups are essentially identical in outcome). If the z-value falls within the intervals (-4.333, -0.007) or (0.007, 4.333), the trial will continue until the following look at the data. We will utilize logistic regression to estimate odds ratios for deterioration between the respective 30 -HOB and 0 -HOB groups. Note that the absence of evaluation is counted as deterioration for the primary endpoint and that, consequently, all randomized participants will have a determined primary endpoint available for the analysis. We will use intent-to-treat principles in our primary data analysis.

Table 1. Stopping boundaries for sequential design.

Look #	Sample Size MT Subjects	Stopping Boundaries (Extended Protocol) Efficacy Z (lower, upper)		Futility Z (lower, upper)	
1	46	4.333	-4.333	0.007	-0.007
2	92	2.963	-2.963	0.374	-0.374
3	138	2.359	-2.359	1.261	-1.261
4	182	2.014	-2.014	2.014	-2.014

2f. Timing of final analysis

The final analysis will occur after all patients have reached the 3-month follow-up period.

2g. Timing of outcome assessments

The study protocol details the procedure schedule, including the expected visit dates and windows.

3. Statistical Principles

3a. Confidence Interval and P-values

All statistical testing will be 2-sided and performed using a 0.05 significance level. We will not adjust for multiple comparisons for the secondary and exploratory outcomes. Hence, p values and their corresponding confidence intervals cannot be used to infer causality. All secondary outcomes will be considered exploratory, and results will be reported with only

effect size estimates and confidence intervals (CIs). All CIs presented will be 95% CI and 2-sided.

3b. Adherence and Protocol Deviations

The Unity system's data capture function with bulk export capability will enable the generation of statistical quality control charts to monitor the proportion of non-conforming patients (delivered treatment different from assigned protocol) over time. Control charts will allow us to quickly assess differences between sites and changes in these proportions as time passes so that improvements can be made swiftly. Control charts will be assembled using Unity's bulk-export function in combination with fully automatically generated reports. These charts' upper and lower control levels allow a ready assessment of whether deviations over time or between hospitals are coincidental or driven by some real difference or shift in-hospital procedures.

3c. Analysis Populations

- **Intention-to-treat (ITT):** The ITT population will include all randomized patients according to the treatment group to which they were assigned at randomization. The primary and secondary outcomes will be analyzed in the ITT population.
- **Safety analysis population:** Serious adverse events (SAE) that will be monitored closely in this trial include 1) Severe neurological deterioration (SND), 2) hospital-acquired pneumonia, and 3) death. Analyses will be descriptive and include rates for SAEs by study cohort. SND is a severe form of deterioration, and consequently, our provision for early trial stopping is also a safety measure. Because SND can be due to several factors not associated with this protocol, incidents of SND will also be reviewed by the Data Safety Monitoring Board (DSMB) and adjudicated to the intervention or control arms as indicated by their findings. Based on our pilots, we exclude cases from enrollment at high risk for pneumonia; these cases are kept NPO for thrombectomy, which will further reduce aspiration risk. Because pneumonia may be due to several different unassociated factors, pneumonia events will be reviewed by DSMB and adjudicated to the intervention as indicated. Death occurring during the active protocol phase, throughout hospitalization, or within 3 months from enrollment will be monitored, and incidents adjudicated by the DSMB. Death may be associated with several unassociated factors. Therefore, the DSMB will carefully determine the association of the study. Other AEs detected will be DSMB reviewed and reported as related or unrelated to study procedures.

4. Trial Population

4a. Screening, recruitment, and withdrawal/follow-up data

The number of screened, randomized patients, the reason for non-randomization, and the time of withdrawal and reasons for withdrawal will be reported for the overall population over the recruitment period (reported in months) as a modified version of the flow diagram (Figure 2) suggested by the CONSORT statement and guidelines.

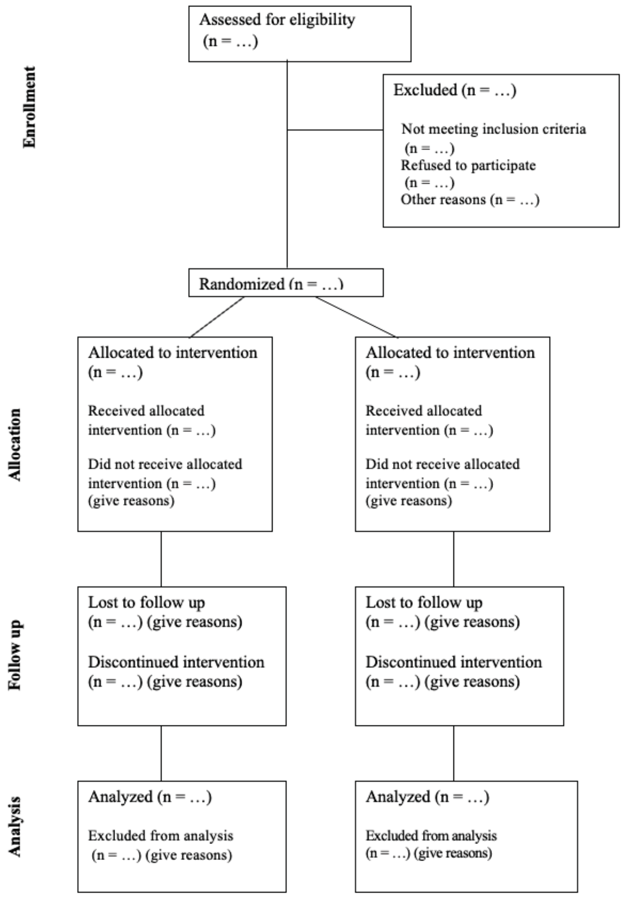

Figure 2. The suggested flow chart is per the CONSORT statement and guidelines[12].

4b. Eligibility Criteria

Table 2: Summary of eligibility criteria.

Inclusion Criteria	Exclusion Criteria
• Adults (>18 years)<br>• Ischemic stroke symptoms consistent with large artery occlusion<br>• Baseline standard of care non-contrast head CT (or MRI) negative for hemorrhage or mass-effect<br>• Evidence of arterial occlusion on standard of care CT angiography or MR angiography<br>• Favorable neuroimaging (Alberta Stroke Program Early Computed Tomography Score<br>• [ASPECTS] > 6 in anterior circulation stroke; not applicable in posterior circulation stroke)<br>• Anticipated treatment with mechanical thrombectomy<br>• Pre-stroke baseline modified Rankin Score (mRS) < 1<br>• Favorable imaging with ASPECTS > 6 and/or favorable CT perfusion scan (according to local site standard of care for thrombectomy patient selection) up to 24-hours from time of symptom onset	• Pregnancy or suspicion of pregnancy<br>• Evidence or suspicion of vomiting any time prior to consent which could predispose to aspiration pneumonia and therefore confound determination of protocol safety<br>• Anticipated palliative care referral<br>• Evidence of evolving malignant infarction on admission noncontrast CT (or MRI)<br>• Need for emergent intubation with mechanical ventilation, or non-invasive ventilatory support with either bi-level positive airway pressure (BiPAP) or continuous positive airway pressure (CPAP) due to pending or actual respiratory failure prior to or at the time of emergency department admission. (Note: Elective intubation for the thrombectomy procedure is not an exclusion criterion.)<br>• Inability to tolerate zero-degree positioning due to congestive heart failure, preexisting pneumonia, chronic obstructive pulmonary disease, or other medical condition. (Note: A diagnosis of heart failure or chronic obstructive pulmonary disease does not automatically exclude enrollment; each patient should be assessed individually for positional intolerance.)<br>• Admission chest radiograph positive for pleural effusion, pulmonary edema, pneumonia, or other pulmonary condition that may confound determination of protocol safety. (Note: An admission chest x-ray is not required, but may be obtained in patients with concerning pulmonary findings.)<br>• Abnormal breath sounds on admission assessment that may confound determination of protocol safety<br>• Lack of a telephone and/or permanent address predisposing patients to be lost to follow up<br>• Enrollment in another clinical trial that may affect our primary or secondary endpoints<br>• In the absence of a consenting legal next of kin, any medical, psychological, cognitive, social or legal condition that would interfere with informed consent and/or capacity to comply with all study requirements, including the necessary time commitment<br>• Note: Enrollment of patients receiving systemic thrombolysis more than 15 minutes prior to randomization is discouraged as this may confound ability to understand the impact of head positioning on clinical stability.

4c. Baseline patient characteristics

Baseline characteristics will be presented in a table (see Table 3), categorized by treatment groups and with the overall population values. Quantitative variables will be summarized as mean (± standard deviation) or, for non-normal distributions, as median (interquartile range). The distributions' normality will be evaluated graphically and using the Shapiro-Wilk test. A paired t-test and Wilcoxon signed-rank test will be employed to compare means and medians, respectively. Categorical variables will be shown as frequencies and percentages and will be compared with Fisher's Exact test.
Additionally, when present, the amount of missing data will be reported. P values are reported for each comparison, with a significance set at <0.05. No correction for multiple comparisons will be made.

Table 3. Summary of patients' baseline characteristics.

	0-Degrees	30-Degrees	p-Value	Overall
N				
Age, mean (SD)				
Female, n (%)				
Race, n (%)				
White				
Black				
Asian				
Hawaiian/Pacific Islander				
Native American/Alaskan				
More than one race				
Arrival or Encounter Mode, n (%)				
Ambulance (%)				
Hospital Transfer (%)				
Private Auto (%)				
Mobile Stroke Unit (%)				
Inpatient Stroke Alert (%)				

	0-Degrees	30-Degrees	p-Value	Overall
Unknown (%)				
Head Position When First Encountered by Study Team, n (%)				
0-Degrees				
30-Degrees				
Past Medical History, n (%)				
Hypertension				
TIA or Stroke				
Diabetes				
Coronary Artery Disease				
Heart Failure				
Cardiac Valvulopathy				
Peripheral Vascular Disease				
Carotid Atheroma				
Carotid Revascularization				
Thyroid Disease				
COPD				
Active Smoker				
Cancer				
Sleep Disordered Breathing				
Alcohol Abuse				
SBP, mean (SD)				
DBP, mean (SD)				
Glucose, mean (SD)				
NIHSS Baseline Time-0, median (IQR)				

	0-Degrees	30-Degrees	p-Value	Overall
ASPECTS, Median (IQR)				
Pre-Stroke Baseline Modified Rankin Score (mRS), n (%)				
mRS 0				
mRS 1				
mRS 2				
mRS 3				
Side of Occlusion, n (%)				
Right				
Left				
N/A (Vertebro-Basilar)				
Bilateral Hemispheres				
Stroke Vascular Territory, n (%)				
Middle cerebral artery				
Internal carotid artery				
Anterior cerebral artery				
Basilar				
Multi-territory				
Systemic Thrombolysis, n (%)				
Alteplase				
Tenecteplase				
Any Systemic Thrombolysis Agent				
Stroke Mechanism, n (%)				
Large Artery Atheroma				
Cardioembolism				
Stroke of Unusual Etiology				

	0-Degrees	30-Degrees	p-Value	Overall
Cryptogenic				
Unknown				
Positive COVID Diagnosis, n (%)				

5. Analysis

5a. Outcome definitions

Primary Outcome: Early neurologic deterioration (END) on the National Institute of Health Stroke Scale occurring during the positioning intervention.

Two or more points worsening in the National Institute of Health Stroke Scale (stroke disability severity measure, ranging from 0-no disability to 42-severely disabled) score during the positioning intervention period. Measured every 10 minutes from initiation of positioning until thrombectomy commences or 2 hours - whichever comes first.

Safety Outcomes:

- **Severe Neurological Deterioration (SND) on the National Institute of Health Stroke Scale Occurring During the Positioning Intervention:** Four or more point-worsening in the National Institute of Health Stroke Scale (stroke disability severity measure, ranging from 0-no disability to 42-severely disabled) scores during the positioning intervention period. Measured every 10 minutes from initiation of positioning until thrombectomy commences or 2 hours - whichever comes first.
- **Diagnosis of Pneumonia Made During Hospitalization in a Patient Free From Pneumonia at Time of Hospital Admission:** Documented onset of a new or progressive infiltrate on pulmonary imaging along with the presence of at least two of the following[13] - Fever of 38°C/100.4°F; Purulent sputum; Leukocytosis or leukopenia; and/or, Decline in oxygen saturation. It is measured until hospital discharge or day 7 (whichever comes first).
- **Participant Deaths at discharge and within 90 days from Stroke Onset:** All-cause death occurring at discharge and from the time of randomization up until 90 days from stroke onset.
- **Post-discharge stroke:** the occurrence of a recurrent stroke within 90 days from stroke onset.

- **Symptomatic intracranial hemorrhage (SICH)** refers to an intracranial hemorrhage that worsens 4 or more points in the NIHSS within 36 hours of receiving tPA or thrombectomy.

Exploratory Outcomes:

- **Improvement at 24 hours and discharge or 7 days after stroke onset:** rates of patients that improved their total National Institute of Health Stroke Scale (stroke disability severity measure, ranging from 0-no disability to 42-severely disabled) score measured at 24 hours, discharge or 7 days after stroke onset.
- **Modified Rankin Scale Score at Hospital Discharge or Day 7:** Categorical rank on the Modified Rankin Scale score (stroke functional outcome measure ranging from 0-no functional disability, to 6-dead) measured at the time of hospital discharge or on day 7 (whichever comes first).
- **Modified Rankin Scale Score at 90 days from Stroke Onset: Categorical rank on the Modified Rankin Scale score (stroke functional outcome measure ranging from 0-no functional disability** to 6-dead) measured at 90 days from stroke onset.
- **Utility-weighted Modified Ranking Scale Score at 90 days from Stroke Onset:** Weighted scores of the original Modified Rankin Scale score where each of the mRS categories (from 0 to 6) is assigned a weight[14] (1.00, 0.91, 0.76, 0.65, 0.33, 0.00 and 0.00).
- **The expanded Thrombolysis in Cerebral Infarction (eTICI) score:** a grading scale used to describe downstream arterial flow (grade 0 = no perfusion noted, grade 1 = reduction in thrombus without any resultant filling of distal arterial branches, grade 2a = reperfusion of 1-49% of the territory, grade 2b50 = reperfusion of 50-66% of the territory, grade 2b67 = reperfusion of 67-89% of the territory, grade 2c = extensive reperfusion of 90-99% of the territory, and grade 3 = complete [100%] reperfusion), will be assessed from catheter angiography taken as the first angiographic run visualizing the lesion and again at conclusion of the thrombectomy procedure. In cases where thrombectomy or other treatment is not planned, the first angiographic run eTICI score will be carried forward as the post-thrombectomy score.

5b. Analysis Methods

- **Primary Outcome:** the primary outcome will be analyzed in the intention-to-treat population. We will use the Log-rank test and unadjusted Cox proportional-hazards modeling to compare and Kaplan-Meier curves to visualize the rate of early neurological deterioration ($\geq$ 2 NIHSS points change from baseline) in the two groups. The number at risk and censored in each group will be reported in the Kaplan-Meier survival curves every 10 minutes. The number needed to harm will be estimated as the inverse of the absolute risk increase (ARI) = 1/ARI.

- **Safety Outcomes:** All the safety outcome analyses will be done in the intention-to-treat population. We will use the Log-rank test, unadjusted Cox proportional-hazards modeling to compare and Kaplan-Meier curves to visualize the rate of severe early neurological deterioration ($\geq$ 4 NIHSS points change from baseline) in the two groups. The number at risk and censored in each group will be reported in the Kaplan-Meier survival curves every 10 minutes. The number needed to harm for SND will be estimated as the inverse of the absolute risk increase (ARI) = 1/ARI. Rates of hospital-acquired pneumonia, discharge, 90-day participant's death, post-discharge stroke, and SICH will be reported in frequencies and percentages. They will be compared between groups using Fisher's Exact test together with odds ratios and the corresponding 95% confidence intervals and p-values from unadjusted logistic regression models that include randomization assignment as the predictor.
- **Exploratory Outcomes: The improvement rates** at 24 hours, discharge/7 days in the NIHSS will be reported as frequencies and percentages. They will be compared between groups using Fisher's Exact test together with odds ratios and the corresponding 95% confidence intervals and p-values from unadjusted logistic regression models that include randomization assignment as the predictor. Changes from baseline NIHSS for individual subjects will be plotted by means of a linear plot according to their randomization group to visualize the changes in NIHSS after randomization in each group. If needed, jitter will be introduced to the data to separate the lines—a repeat measures ANOVA will be employed to compare the repeat NIHSS measures between the two groups.

  90-day mRS will be analyzed in three ways:

1. Dichotomized as 0-2/3-6 and 0-3/4-6 and modeled as a dependent variable by unadjusted logistic regression with randomization assignment as the predictor. 90-day mRS will be plotted using a paired horizontal bar graph (aka 'Grotta bars') for visualization purposes.
2. Analyzed as separate categories and modeled by unadjusted ordinal logistic regression as a dependent variable and with randomization assignment as the predictor. Here, the proportional odds assumption will be verified by likelihood ratio testing.
3. Transformed to a utility-weighted mRS (UW-mRS) where each of the mRS categories (from 0 to 6) is assigned a weight[14] (1.00, 0.91, 0.76, 0.65, 0.33, 0.00 and 0.00) and modeled as a dependent variable by unadjusted linear regression, with randomization assignment as the predictor.

No adjusting covariates were pre-specified for any of the analyzed outcomes. Except when dealing with missing data, no sensitivity or subgroup analysis was pre-specified for any of the above-described outcomes. We will not adjust for multiple comparisons for the secondary and exploratory outcomes. Hence, p values and their corresponding confidence intervals will not be used to infer causality. Outcomes will be reported in a table (see Table 4).

Table 4: Summary of primary, safety, and secondary (exploratory) outcomes.

Outcome	0-Degrees	30-Degrees	Unadjusted Hazard/Odds Ratio (95% CI)	P Value
**Primary Outcome, n (%)**				
Early neurological deterioration (≥ 2 points from baseline NIHSS)				
**Safety Outcomes, n (%)**				
Worsening of ≥ 4 points from baseline NIHSS				
Hospital Acquired Pneumonia				
Death at Discharge/Day 7				
90 day all-cause mortality				
Post-discharge stroke				
Symptomatic intracerebral hemorrhage (SICH)				
**Pre-specified Exploratory Outcomes, n (%)**				
Improvement at 24h				
Improvement at Discharge/Day 7				
90 day mRS 0-1				
90 day mRS 0-2				
90 day mRS 0-3				
90 day mRS, median (IQR)				
UW-mRS, mean (SD)				

5c. Missing Data

Missing data will be managed using strategies to minimize its impact on the analysis. First, efforts will be made during data collection to ensure completeness, including thorough staff training and robust data monitoring. The extent and patterns of missingness will be assessed if missing data occurs. For variables with sporadic missing values, we will assume data was missing at random and will apply multiple imputation to preserve statistical power and reduce bias. If the data are found to be missing, not at random, sensitivity analyses will be conducted to evaluate how different assumptions about the missing data mechanism may affect the results. Complete case analysis will also be considered for comparison to ensure consistency of findings across different approaches. The number and percentage of missing data will be reported, and any systematic patterns of missingness will be examined and documented.

5d. Statistical Software

All statistical analyses will be performed using R version 4.3.1 (© R Foundation for Statistical Computing, 2023). A fully annotated R Mark Down document and the trial raw dataset will be available upon request for replication and verification purposes.

6. References

25. Toole JF. Effects of change of head, limb and body position on cephalic circulation. N Engl J Med. 1968 Aug 8;279(6):307-11. doi: 10.1056/NEJM196808082790609. PMID: 5660303.
26. Caplan LR, Sergay S. Positional cerebral ischaemia. J Neurol Neurosurg Psychiatry. 1976 Apr;39(4):385-91. doi: 10.1136/jnnp.39.4.385. PMID: 932755; PMCID: PMC492290.
27. Hayashida K, Hirose Y, Kaminaga T, Ishida Y, Imakita S, Takamiya M, Yokota I, Nishimura T. Detection of postural cerebral hypoperfusion with technetium-99m-HMPAO brain SPECT in patients with cerebrovascular disease. J Nucl Med. 1993 Nov;34(11):1931-5. PMID: 8229237.
28. Ouchi Y, Nobezawa S, Yoshikawa E, Futatsubashi M, Kanno T, Okada H, Torizuka T, Nakayama T, Tanaka K. Postural effects on brain hemodynamics in unilateral cerebral artery occlusive disease: a positron emission tomography study. J Cereb Blood Flow Metab. 2001 Sep;21(9):1058-66. doi: 10.1097/00004647-200109000-00003. PMID: 11524610.
29. Wojner AW, El-Mitwalli A, Alexandrov AV. Effect of head positioning on intracranial blood flow velocities in acute ischemic stroke: a pilot study. Crit Care Nurs Q. 2002 Feb;24(4):57-66. doi: 10.1097/00002727-200202000-00007. PMID: 11833629.
30. Wojner-Alexander AW, Garami Z, Chernyshev OY, Alexandrov AV. Heads down: flat positioning improves blood flow velocity in acute ischemic stroke. Neurology. 2005 Apr 26;64(8):1354-7. doi: 10.1212/01.WNL.0000158284.41705.A5. PMID: 15851722.
31. Saqqur M, Sharma VK, Tsivgoulis G, Huy TN, Heliopoulos I, Siddiqui M, Derksen C, Khan K, Alexandrov AV. Real-time hemodynamic assessment of downstream effects of intracranial stenoses in patients with orthostatic hypoperfusion syndrome. Cerebrovasc Dis. 2010;30(4):355-61. doi: 10.1159/000319567. Epub 2010 Aug 5. PMID: 20693790; PMCID: PMC3014861.
32. Hunter AJ, Snodgrass SJ, Quain D, Parsons MW, Levi CR. HOBOE (Head-of-Bed Optimization of Elevation) Study: association of higher angle with reduced cerebral blood flow velocity in acute ischemic stroke. Phys Ther. 2011 Oct;91(10):1503-12. doi: 10.2522/ptj.20100271. Epub 2011 Aug 25. PMID: 21868612.
33. Favilla CG, Mesquita RC, Mullen M, Durduran T, Lu X, Kim MN, Minkoff DL, Kasner SE, Greenberg JH, Yodh AG, Detre JA. Optical bedside monitoring of cerebral blood flow in acute ischemic stroke patients during head-of-bed manipulation. Stroke. 2014 May;45(5):1269-74. doi: 10.1161/STROKEAHA.113.004116. Epub 2014 Mar 20. PMID: 24652308; PMCID: PMC4006296.
34. Ali LK, Weng JK, Starkman S, Saver JL, Kim D, Ovbiagele B, Buck BH, Sanossian N, Vespa P, Bang OY, Jahan R, Duckwiler GR, Viñuela F, Liebeskind DS. Heads Up! A Novel Provocative Maneuver to Guide Acute Ischemic Stroke Management. Interv Neurol. 2017 Mar;6(1-2):8-15. doi: 10.1159/000449322. Epub 2016 Sep 30. PMID: 28611828; PMCID: PMC5465685.

35. Chen HS, Zhang NN, Cui Y, Li XQ, Zhou CS, Ma YT, Zhang H, Jiang CH, Li RH, Wan LS, Jiao Z, Xiao HB, Li Z, Yan TG, Wang DL, Nguyen TN. A randomized trial of Trendelenburg position for acute moderate ischemic stroke. Nat Commun. 2023 May 5;14(1):2592. doi: 10.1038/s41467-023-38313-y. PMID: 37147320; PMCID: PMC10163013.
36. Schulz KF, Altman DG, Moher D, et al: CONSORT 2010 Statement: updated guidelines for reporting parallel group randomised trials. BMC Medicine 2010; 8(1):18
37. American Thoracic Society; Infectious Diseases Society of America. Guidelines for the management of adults with hospital-acquired, ventilator-associated, and healthcare-associated pneumonia. Am J Respir Crit Care Med. 2005 Feb 15;171(4):388-416. doi: 10.1164/rccm.200405-644ST. PMID: 15699079.
38. Chaisinanunkul N, Starkman S, Gornbein J, Hamilton S, Chatfield F, Conwit R, Saver JL. Staged use of ordinal and linear disability scales: a practical approach to granular assessment of acute stroke outcome. Front Neurol. 2023 Jun 28;14:1174686. doi: 10.3389/fneur.2023.1174686. Erratum in: Front Neurol. 2023 Dec 12;14:1331276. doi: 10.3389/fneur.2023.1331276. PMID: 37456628; PMCID: PMC10344771.
