# Supplementary material for: Optimal Head-of-Bed Positioning Before Thrombectomy in Large Vessel Occlusion Stroke: A Randomized Clinical Trial
Source: JAMA Neurol. 2025 Jun 4;82(9):905–14. doi: 10.1001/jamaneurol.2025.2253 (PMC12138796; doi:10.1001/jamaneurol.2025.2253)
Supplement: Supplement 3. — eTable 1. Protocol Amendment eTable 2. Adjusted Hazard Ratios for the Primary Outcome >2 Points NIHSS Worsening (END) and the Safety End Point >4 Points NIHSS Worsening (SND) eTable 3. Thrombectomy Treatment Characteristics eFigure. Exploratory 90-Day Modified Rankin Score (mRS) Analyses [file jamaneurol-e252253-s003.pdf]

# Supplementary Online Content

Alexandrov AW, Shearin AJ, Mandava P, et al; ZODIAC Investigators. Optimal head-of-bed positioning before thrombectomy in large vessel occlusion stroke: a randomized clinical trial. *JAMA Neurol*. Published online June 4, 2025.
doi:10.1001/jamaneurol.2025.2253

**eTable 1.** Protocol Amendment

**eTable 2.** Adjusted Hazard Ratios for the Primary Outcome ≥2 Points NIHSS Worsening (END) and the Safety End Point ≥4 Points NIHSS Worsening (SND)

**eTable 3.** Thrombectomy Treatment Characteristics

**eFigure.** Exploratory 90-Day Modified Rankin Score (mRS) Analyses

This supplementary material has been provided by the authors to give readers additional information about their work.

© 2025 Alexandrov AW et al. *JAMA Neurology*.

eTable 1: Protocol Amendment

| Number | Date Implemented | Protocol Component Changed | Original Process | Amended Process |
|---|---|---|---|---|
| 1 | 09/16/2019 | Inclusion Criterion | Symptom onset within 6-hours of start time for catheter angiography/ planned thrombectomy procedure | Favorable imaging with ASPECTS $\geq$ 6 and/or favorable CT perfusion scan (according to local site standard of care for thrombectomy patient selection) up to 24-hours from time of symptom onset |

© 2025 Alexandrov AW et al. *JAMA Neurology*.

eTable 2. Adjusted hazard ratios for the primary outcome ≥2 points NIHSS worsening (END) and the safety endpoint ≥4 points NIHSS worsening (SND).

| Models | Adjusted Hazards Ratio (95% CI) | P Value |
|---|---|---|
| END ~ Randomization Group + Time 0 NIHSS + Enrollment Site | 38.3 (5.13 – 286) | <0.001 |
| END ~ Randomization Group + Age + Sex + Time 0 NIHSS + Time of stroke onset to arrival | 67.1 (4.80 – 937) | 0.002 |
| END ~ Randomization Group + Hemisphere of stroke | 30.7 (4.14 – 229) | <0.001 |
| END ~ Randomization Group + Vertebrobasilar strokes | 34.4 (4.66 – 255) | <0.001 |
| END ~ Randomization Group + Time of stroke onset to thrombectomy | 52.7 (3.88 – 716) | 0.003 |
| SND ~ Randomization Group + Time 0 NIHSS + Enrollment Site | 25.7 (3.40–194) | 0.002 |

Note: Adjusted hazards ratio (HR) models for the primary endpoint (early neurological deterioration [END] ≥ 2 points worsening on the National Institutes of Health Stroke Scale [NIHSS]) and the safety endpoint (severe neurological deterioration [SND] ≥ 4 points NIHSS worsening) were not pre-specified in the ZODIAC statistical analysis plan and were not indicated due to balanced patient characteristics. However, we chose to include adjustments for items that readers may question in this supplemental table. Row one adjusts the primary endpoint HR for the "time 0" NIHSS score and enrollment site. Row two adjusts the primary endpoint HR for age, sex, time 0 NIHSS and time of stroke onset to arrival. Row three adjusts the primary endpoint HR for hemisphere of stroke. Row four adjusts the primary endpoint HR for vertebrobasilar strokes. Row five adjusts the primary endpoint HR for time of stroke onset to thrombectomy. Row six adjusts the HR for the safety endpoint SND by time 0 NIHSS and enrollment site.

© 2025 Alexandrov AW et al. *JAMA Neurology*.

eTable 3: Thrombectomy Treatment Characteristics

| | 0-Degrees | 30-Degrees | p-Value | Overall |
|---|---|---|---|---|
| Patient Not Taken to Catheterization Lab at Direction of Neurointerventionalist, n (%) | 4 (8.89) | 2 (4.26) | 0.43 | 6 (6.52) |
| Baseline Starting eTICI Score, n (%) | N = 40 | N = 42 | 0.600 | N = 82 |
| eTICI 0 | 34 (85.00) | 38 (90.48) | | 72 (87.80) |
| eTICI 1 | 0 | 0 | | 0 |
| eTICI 2a | 3 (7.50) | 1 (2.38) | | 4 (4.88) |
| eTICI 2b50 | 0 | 1 (2.38) | | 1 (1.22) |
| eTICI 2b67 | 0 | 0 | | 0 |
| eTICI 2c | 2 (5.00) | 2 (4.76) | | 4 (4.88) |
| eTICI 3 | 1 (2.50) | 0 | | 1 (1.22) |
| Baseline eTICI score unavailable, n (%) | 1 (2.22) | 3 (6.38) | | 4 (4.35) |
| All Baseline Starting 2b-3 eTICI Scores, n (%) | 3 (7.50) | 3 (7.14) | 1.00 | 6 (7.32) |
| Thrombectomy Procedure Not Performed After Angiographic First Run, n (%) | 10 (22.22) | 4 (8.51) | 0.09 | 14 (15.22) |
| Total Patients Undergoing Mechanical Thrombectomy, n (%) | 30 (66.67) | 39 (82.98) | 0.09 | 69 (75) |
| Final eTICI Scores in Patients Undergoing Thrombectomy, n (%) | N = 30 | N = 39 | 0.52 | N = 69 |
| eTICI 0 | 0 | 2 (5.13) | | 2 (2.89) |
| eTICI 1 | 0 | 0 | | 0 |

© 2025 Alexandrov AW et al. *JAMA Neurology*.

| | | | | |
|---|---|---|---|---|
| eTICI 2a | 0 | 3 (7.69) | | 3 (4.35) |
| eTICI 2b50 | 1 (3.33) | 2 (5.13) | | 3 (4.35) |
| eTICI 2b67 | 9 (30.00) | 10 (25.64) | | 19 (27.54) |
| eTICI 2c | 15 (50.00) | 14 (35.90) | | 29 (42.03) |
| eTICI 3 | 5 (16.67) | 8 (20.51) | | 13 (18.84) |
| All Final Thrombectomy 2b50-3 eTICI Scores, n (%) | 30 (100.00) | 34 (87.18) | 0.06 | 64 (92.75) |
| Final eTICI Scores After Thrombectomy, Including Carryover of Baseline eTICI Scores for Patients Not Undergoing Thrombectomy, n (%) | N = 40 | N = 43 | 0.70 | N = 83 |
| eTICI 0 | 6 (15.00) | 3 (6.98) | | 9 (10.84) |
| eTICI 1 | 0 | 0 | | 0 |
| eTICI 2a | 1 (2.50) | 3 (6.98) | | 4 (4.82) |
| eTICI 2b50 | 1 (2.50) | 3 (6.98) | | 4 (4.82) |
| eTICI 2b67 | 9 (22.50) | 10 (23.26) | | 19 (22.89) |
| eTICI 2c | 17 (42.50) | 16 (37.20) | | 33 (39.76) |
| eTICI 3 | 6 (15) | 8 (18.60) | | 14 (16.87) |
| All Final eTICI 2b50-3 Scores Including Carryover for Patients Not Undergoing Thrombectomy, n (%) | 33 (82.50) | 37 (86.05) | 0.77 | 70 (84.34) |
| Head Positioning Ordered at 24 Hours from Randomization, n (%) | | | 0.32 | |
| 0-Degree | 30 (66.67) | 24 (51.06) | | 54 (58.70) |
| 30-Degree | 10 (22.22) | 11 (23.40) | | 21 (22.82) |
| Out of Bed to Chair | 3 (6.67) | 6 (12.77) | | 9 (9.78) |
| | 2 (4.44) | 6 (12.77) | | 8 (8.70) |

© 2025 Alexandrov AW et al. *JAMA Neurology*.

| | | | | |
|---|---|---|---|---|
| No Specific Head Position or Mobilization Activity Ordered | | | | |

Note: eTICI = expanded Thrombolysis in Cerebral Infarction score.

© 2025 Alexandrov AW et al. *JAMA Neurology*.

eFigure. Exploratory 90-day modified Rankin Score (mRS) analyses.

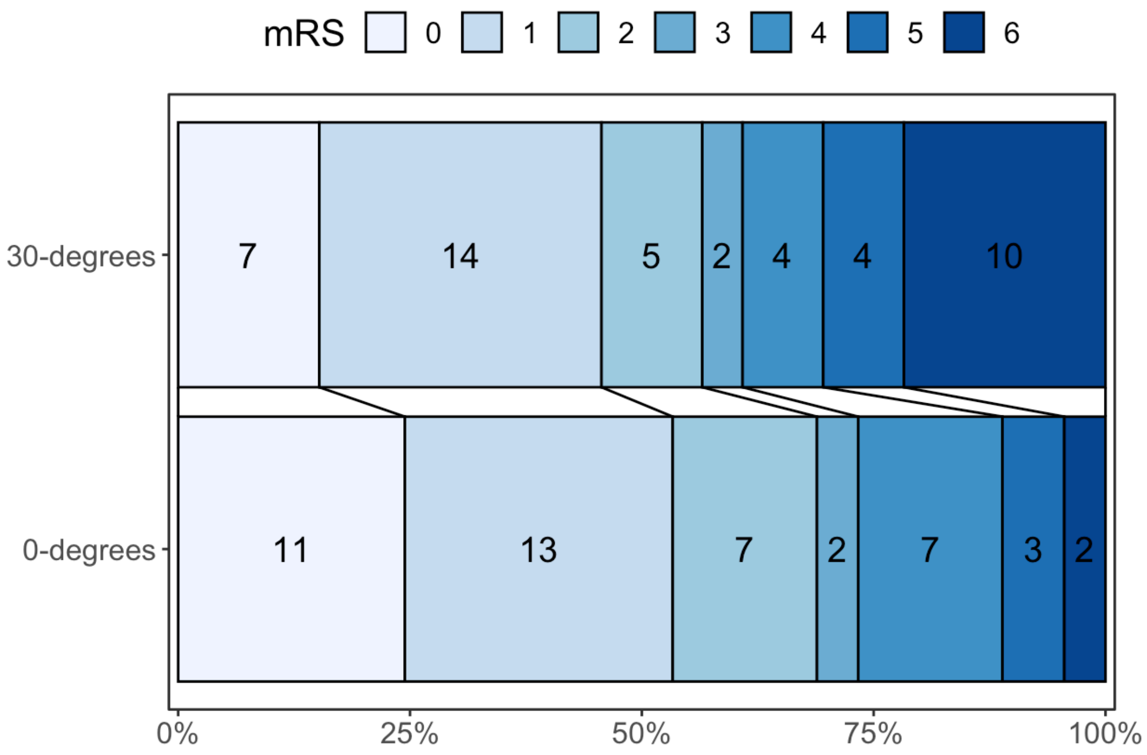


© 2025 Alexandrov AW et al. *JAMA Neurology*.
