# Supplementary material for: Optimal Head-of-Bed Positioning Before Thrombectomy in Large Vessel Occlusion Stroke: A Randomized Clinical Trial
Source: JAMA Neurol. 2025 Jun 4;82(9):905–14. doi: 10.1001/jamaneurol.2025.2253 (PMC12138796; doi:10.1001/jamaneurol.2025.2253)
Supplement: Supplement 4. — Nonauthor Collaborators. ZODIAC Investigators. [file jamaneurol-e252253-s004.pdf]

Supplemental Online Content: Nonauthor Collaborators
*First name, last name, and suffix (if applicable) are required and will appear in PubMed.

*Group Name(s): ZODIAC Investigators

***First Name and Middle Initial(s)**	***Last Name**	***Suffix (eg, Jr, III)**	Academic Degrees	Institution	Location (city, state/province, country)	Role or Contribution, eg, chair, principal investigator	Group (if more than 1 Group listed in the byline) and/or Subgroup (eg, Steering Committee)
Erin	Cekovich		MSN	Penn State University Medical Cente	Hershey, PA	data collection and ent	ZODIAC Investigators
Summer	Culbert		DNP	Johnson City Medical Center	Johnson City, TN	data collection and ent	ZODIAC Investigators
Felicia	Dillard		DNP	Baptist Memorial Medical Center	Memphis, TN	data collection and ent	ZODIAC Investigators
Abbigayle	Doerr		DNP	Northwestern Medicine Central DuPa	Winfield, IL	data collection	ZODIAC Investigators
James W.	Jaffe		MD	Doctors Medical Center of Modesto,	Modesto, CA	data collection	ZODIAC Investigators
Ann	Jerde		BSN	University of Louisville Medical Cente	Louisville, KY	data collection and ent	ZODIAC Investigators
Marc D.	Malkoff		MD	University of Arizona, Phoenix	Phoenix, AZ	design and editing	ZODIAC Investigators
E. Jeffrey	Metter		MD	University of Tennessee Health Scier	Memphis, TN	design and editing	ZODIAC Investigators
Jacqueline	Owens		MHA	Mobile Infirmary	Mobile, TN	data collection and ent	ZODIAC Investigators
Stacie	Stevens		PhD	Virginia Commonwealth University M	Richmond, VA	data collection and ent	ZODIAC Investigators
Elizabeth	Wise		MSN	University of Louisville Medical Cente	Louisville, KY	data collection	ZODIAC Investigators
Cesar	Velasco		BSN	Penn State University Medical Cente	Hershey, PA	data collection and ent	ZODIAC Investigators
Xifeng (Spring)	Xu		MSN	El Camino Health	Mountainview, CA	data collection and ent	ZODIAC Investigators
