# Supplementary material for: Optimal Head-of-Bed Positioning Before Thrombectomy in Large Vessel Occlusion Stroke: A Randomized Clinical Trial
Source: JAMA Neurol. 2025 Jun 4;82(9):905–14. doi: 10.1001/jamaneurol.2025.2253 (PMC12138796; doi:10.1001/jamaneurol.2025.2253)
Supplement: Supplement 5. — Data Sharing Statement. [file jamaneurol-e252253-s005.pdf]

Data Sharing Statement

Alexandrov. Optimal Head-of-Bed Positioning Before Thrombectomy in Large Vessel Occlusion Stroke. *JAMA Neurol*. Published June 04, 2025. doi:10.1001/jamaneurol.2025.2253

Data
Additional Information: Clinical Trials.gov: NCT03728738; https://clinicaltrials.gov/study/NCT03728738?locStr=Memphis,%20TN&country=United%20States&state=Tennessee&city=Memphis&cond=Stroke&term=head%20position&rank=1
Data available: Yes
Data types: Deidentified participant data, Data dictionary
How to access data: Interested parties should contact the PI at aalexa33@uthsc.edu.
When available: beginning date: 06-30-2026

Supporting Documents
Document types: Statistical/analytic code, Informed consent form
How to access documents: Interested parties should contact the PI at aalexa33@uthsc.edu.
When available: beginning date: 06-30-2026, end date: 06-30-2027

Additional Information
Who can access the data: Interested parties should contact the PI at aalexa33@uthsc.edu.
Types of analyses: Meta-analyses
Mechanisms of data availability: With investigator support and after approval of a proposal and signed data access agreement.
